# Supplementary material for: The characteristics of bacteremia among patients with acute febrile illness requiring hospitalization in Indonesia
Source: PLoS One. 2022 Sep 8;17(9):e0273414. doi: 10.1371/journal.pone.0273414 (PMC9455855; doi:10.1371/journal.pone.0273414)
Supplement: Attachment — Submitted filename: PONE-D-22-07517_R2.pdf [file pone.0273414.s003.pdf]

PLOS ONE

The Characteristics of Bacteremia among Patients with Acute Febrile Illness Requiring Hospitalization in Indonesia

--Manuscript Draft--

**Manuscript Number:**	PONE-D-22-07517R1
**Article Type:**	Research Article
**Full Title:**	The Characteristics of Bacteremia among Patients with Acute Febrile Illness Requiring Hospitalization in Indonesia
**Short Title:**	Bacteremia among Patients with Acute Febrile Illness Requiring Hospitalization in Indonesia
**Corresponding Author:**	Herman Kosasih, Ph.D<br>INA-Respond<br>Jakarta, DKI Jakarta INDONESIA
**Keywords:**	bacteremia; Bloodstream infection; Acute febrile illness; hospitalization; Indonesia
**Abstract:**	Blood culturing remains the “gold standard” for bloodstream infection (BSI) diagnosis, but the method is inaccessible to many developing countries due to high costs and insufficient resources. To better understand the utility of blood cultures among patients in Indonesia, a country where blood cultures are not routinely performed, we evaluated data from a previous cohort study that included blood cultures for all participants. An acute febrile illness study was conducted from July 2013 to June 2016 at eight major hospitals in seven provincial capitals in Indonesia. All participants presented with a fever, and two-sided aerobic blood cultures were performed within 48 hours of hospital admission. Positive cultures were further assessed for antimicrobial resistance (AMR) patterns. Specimens from participants with negative culture results were screened by advanced molecular and serological methods for evidence of causal pathogens. Blood cultures were performed for 1,459 of 1,464 participants, and the 1,030 (70.6%) participants that were negative by dengue NS1 antigen test were included in further analysis. Bacteremia was observed in 92 (8.9%) participants, with the most frequent pathogens being Salmonella spp. (51), Escherichia coli (14), and Staphylococcus aureus (10). Two Salmonella spp. cases had evidence of AMR, and several E. coli cases were multidrug resistant (6/14, 42.9%) or monoresistant (2/14, 14.3%). Culture contamination was observed in 37 (3.6%) cases. Advanced laboratory assays identified ~~culturable pathogens~~ in participants having negative cultures, with 23.1% to 90% of cases being missed by blood cultures. Blood cultures are a valuable diagnostic tool for hospitalized patients presenting with fever. In Indonesia, pre-screening patients for the most common viral infections, such as dengue, influenza, and chikungunya viruses, would maximize the benefit to the patient while also conserving resources. Blood cultures should also be supplemented with advanced laboratory tests when available.
**Order of Authors:**	Pratiwi Soedarmono
	Aly Diana
	Patricia Tauran
	Dewi Lokida
	Abu Tholib Aman
	Bachti Alisjahbana
	Dona Arlinda
	Emiliana Tjitra
	Herman Kosasih, Ph.D
	Ketut Tuti Parwati Merati
	Mansyur Arif

etiological agent

mention serovar name was it Typhi?

mention which advanced techniques

Powered by Editorial Manager® and ProduXion Manager® from Aries Systems Corporation

	Muhammad Hussein Gasem
	Nugroho Harry Susanto
	Nurhayati Lukman
	Retna Indah Sugiyono
	Usman Hadi
	Vivi Lisdawati
	Karine G Fouth Tchos
	Aaron Neal
	Muhammad Karyana
**Opposed Reviewers:**	
**Response to Reviewers:**	Dear Dwij Raj Bhatta, PhD<br>Academic Editor<br>PLOS ONE<br><br>Thank you very much for the constructive comments and suggestions provided by the reviewers. We have carefully revised the manuscript following the suggestions. Please see the response to each comment/suggestion below.<br><br>Reviewer #1: This compilation of data from different centers over many years is commendable. This highlights the issues faced in diagnostic microbiology in developing countries. It is an interesting paper with important observations and discussions. Some spellings need review and correction. Recommend to submit after corrections.<br><br>Response: Thank you very much for your comments, we really appreciate it.<br>We have corrected the spelling errors.<br><br>Reviewer #2: The Characteristics of Bacteremia among Patients with Acute Febrile Illness Requiring Hospitalization in Indonesia.<br><br>Evaluation. This report addresses an important subject in Bacteriemia and Acute Febrile illness; i.e., the worrying trend of antimicrobial resistance in bacterial pathogens (Salmonella and Non Salmonella spp) . It reports the frequency and distribution of bacterial pathogens in blood culture and its susceptibility pattern isolated from various specimens from a seven medical center in Indonesia, from which similar reports are scarce. Though it is better attempt by Soedarmono et al., to know information on bacteremia and other causative agent of Acute Febrile illeness in Indonesia.<br><br>Response: Thank you very much for your comments, we really appreciate it.<br><br>Comments<br><br>1.Give rationale of the study? Why is NS1 antigen screening only performed? What about other viral agents related AFI?<br><br>Response: We have added more information regarding this issue in the Methods.<br><br>Lines 135-146 now read: During the baseline visit, blood was collected for cultures, clinically relevant rapid diagnostic tests when available, and dengue virus rapid diagnostic tests. Dengue virus infection remains a significant burden across Indonesia [28,29], with disease incidence increasing in recent years [30]. Though other viral agents are present in Indonesia, none are as prevalent as dengue virus [24,31], and most are challenging to diagnose due to limitations with available rapid diagnostic tests [32,33]. Given the widespread prevalence of dengue virus infection, and the very high specificity (almost 100%) and good sensitivity (70-87%) of NS1 antigen rapid diagnostic tests [34], we employed universal dengue virus screening to rapidly resolve the unknown etiologies of study participants. Participants with negative NS1 antigen tests were further considered for BSIs through blood culture tests and other etiologies,

as determined through advanced testing at the INA-RESPOND reference laboratory.

2.Why you performed Blood culture 0f 1459 Cases? You have mentioned 1464 were enrolled? What about 5??

Response: We only performed blood culture for 1459 patients, as the remaining 5 subjects did not have enough blood for blood culture test.

Lines 207-210 now read: The remaining 5 participants had insufficient blood specimens for following reasons: 1 adult was in a severe condition (decreased of consciousness), 2 participants (1 child and 1 adult) self-discharged against medical advice, and the guardians of 2 children refused to allow more blood to be drawn.
3.At the end of introduction, please give some update of Acute Febrile illness and their epidemiology in Indonesia.

Response: Thank you very much for the suggestion. We have added some update of acute febrile illness and their epidemiology in Indonesia.

Lines 97-111 now read: The epidemiology of pathogens associated with fever in Indonesia is not well understood, as public health surveillance data is limited and only a few local studies have been conducted [19,21–26]. Among published studies, dengue virus, chikungunya virus, influenza virus, Salmonella Typhi, Rickettsia spp., and Leptospira spp. are consistently the most common causes of acute febrile illness hospitalizations. A study in Papua from November 1997 to February 2000 enrolled 226 hospitalized patients that were negative for malaria, the majority of whom were determined to have typhoid fever (18%), leptospirosis (12%), rickettsioses (8%), and dengue fever (7%) [23]. An observational fever study in Bandung identified dengue virus in 12.4% of fever episodes, followed by S. Typhi (7.4%), and chikungunya virus (7.1%) [24,26,27]. A 2005-2006 study in Semarang found rickettsioses and leptospirosis in 7% and 10%, respectively, of 137 acute undifferentiated fever cases [21]. The parent study of the research presented here found the most prevalent pathogens among participants at eight hospitals in 7 major cities in Indonesia to be dengue virus (27-52%), Rickettsia spp. (2-12%), S. Typhi (0.9-13%), influenza virus (2-6%), Leptospira spp. (0-5%), and chikungunya virus (0-4%) [19].

4.Which are the hospitals included in the study, please mentions the name of hospitals.

Response: We have included the name of hospitals in the Methods.

Lines 121-127 now read: A prospective observational study enrolling febrile patients who required hospitalization was conducted by the Indonesia Research Partnership on Infectious Disease (INA-RESPOND) from July 2013 to June 2016 at eight major hospitals in seven provincial capitals in Indonesia: Dr. Cipto Mangunkusumo Hospital in Jakarta, Sulianti Saroso Infectious Disease Hospital in Jakarta, Dr. Wahidin Sudirohusodo Hospital in Makassar, Dr. Sardjito Hospital in Yogyakarta, Dr. Hasan Sadikin Hospital in Bandung, Sanglah General Hospital in Denpasar, Dr. Soetomo Hospital in Surabaya, and Dr. Kariadi Hospital, in Semarang.

5.How do you calculate sample size? Is it sufficient to draw conclusion regarding bacteremia (causative bacterial pathogens) in Indonesia?

Response: As this study was an observational study to find etiologies of acute febrile illness during a certain period of time (2013-2016), we did not specifically calculate the sample size for drawing the conclusion regarding bacteremia in Indonesia. Since we performed the analysis of blood culture results from almost all participants (>99% participants, approximately 100 adults and 100 children from each hospital), though cannot be generalizable to the Indonesian population at-large, we expected that the data will provide better understanding of the bacteremia in hospitalized population with fever and hopefully will lead to a reduction in mortality from BSIs.

6.What is your inclusion and exclusion criteria? Please mention Clearly.

Response: We have added the inclusion and exclusion criteria.

Powered by Editorial Manager® and ProduXion Manager® from Aries Systems Corporation

Lines 128-131 now read: Briefly, inclusion criteria consisted of axillary body temperature 38˚C, 1 year of age, and hospitalization within the past 24 hours. Patients were excluded from the study if they had subjective fever for 14 days or were hospitalized in the last 3 months.

7.Please give the ethical approval committee name and approval number and date.

Response: The name of the ethical approval committee and approval number had already provided under the “Ethical Clearance” (lines 197-203); and we have added the date.

Ethical approvals for the AFIRE study were granted by the Institutional Review Boards of the National Institute of Health Research and Development (NIHRD), Indonesia Ministry of Health (KE.01.05/EC/407/2012) dated 23 May 2012, the Faculty of Medicine at the University of Indonesia and RSUPN Dr. Cipto Mangunkusumo Hospital (451/PT02.FK/ETIK/2012) dated 23 July 2012, and RSUD Dr. Soetomo Hospital (192/Panke.KKE/VIII/2012) dated 13 August 2012.

8.How do assure the Quality controls and quality check of your results, either BD 135 Phoenix (Becton Dickinson) or VITEK 2 (bioMérieux, Inc., Durham, North Carolina), System?

Response: Blood culture tests were performed at the hospital's accredited clinical laboratory, which provides patient diagnostic services. All instruments and standards were calibrated appropriately according to manufacturer guidelines. Every site’s laboratory performed quality control (QC) to ensure proper performance and sent the QC report to protocol team to be reviewed. All tests were run alongside appropriate positive and negative control to ensure the integrity and accuracy of the results. For example, QC for VITEK 2 system; each new lot number of ID cards is tested with stock culture organisms. Susceptibility cards are tested weekly against stock culture organisms.

The QC organisms uses as follows:

Weekly:
AST-GP 67 cards
Enterococcus faecalis ATCC 29212
AST-GN 66 cards
E. coli ATCC 25922 non fermenter
PSA ATCC 27853 fermenter
E. coli ATCC 35218 non fermenter
ID-NH cards
Elkenella corrodens ATCC BAA-1152
New Lots:
ID-GP cards
Enterococcus casseliflavis ATCC 700327
ID-GN cards
Enterobacter hormechei (E.cloacae) ATCC 700323

Lines 163-171 now read: Blood cultures were performed and analyzed at the hospitals’ nationally accredited clinical laboratories by trained, certified staff. All instruments and standards were calibrated appropriately according to manufacturer guidelines, and all tests were run alongside appropriate positive and negative control to ensure the integrity and accuracy of the results. Organism identification was considered acceptable when the confidence level in the automated growth identification system was ≥95% probability [34]. Quality control tests were performed weekly at all site laboratories, and each new lot of ID cards was tested using validated stocks of culture organisms.

9.What is the volume of blood sample collected and used in culture from children and adults?

Response: This is already stated in the text. Blood volumes of approximately 5-8 mL for adults and 1-3 mL for children were collected from each arm, whenever possible,

Powered by Editorial Manager® and ProduXion Manager® from Aries Systems Corporation

	directly into separate aerobic blood culture bottles (lines 150-152).<br><br>10.It is better to give numerator value after percentage values.<br><br>Response: We have changed the presentation throughout the manuscript.<br><br>11.Please give the full name of bacteria initially such as Staphylococcus aureus and then short form S. aureus and other bacteria throughout the manuscript.<br><br>Response: We have followed your suggestion.<br><br>12.Please mention the more information on infections with dengue virus and bacteremia in Indonesia.<br><br>Response: We found no dengue virus and bacteremia co-infection in our study, as mentioned in the Discussion. We have added more informations about dengue virus and bacteremia.<br><br>Lines 355-368 now read: Data on co-infections with dengue virus and bacteremia is limited. A literature review of published case reports and studies from January 1943 to March 2016 found 3 studies in Singapore and Taiwan reporting concurrent bacteremia in 0.18-7% of dengue fever cases [40–42]. A concurrent dengue virus and S. Typhi case was also reported from Bandung, Indonesia [43]. In all of these studies, blood was collected for bacterial culture because patients did not improve clinically a few days to a week after dengue fever was diagnosed. Furthermore, in the majority of cases, dengue virus infection was confirmed by serology only (IgM detected or four-fold IgG increase). These reports support our finding that simultaneous infection with bacteria and dengue virus is rare. In our study, bacterial growth observed in 14 participants with positive dengue NS1 antigen tests were considered false positive blood cultures (5 Staphylococcus hominis, 4 Staphylococcus epidermidis, 1 Kocuria rosea, 1 Micrococcus aureus, 1 Staphylococcus arlettae, 1 coagulase-negative Staphylococcus spp., and 1 Staphylococcus waneri).<br><br>13.Please corelate conclusion with your findings.<br><br>Response: Thank you very much, we have correlated our conclusion with our findings.<br><br>Lines 522-541 now read: We presented aerobic blood culture findings from a multi-centre study of patients with acute febrile illness admitted to eight major hospitals across Indonesia. Our universal use of aerobic blood cultures is unique in Indonesia, the results of which help clarify the epidemiology and burden of BSI, rates of contamination among CAI, and common AMR patterns in Indonesia. Bacteremia was observed in 8.9% participants, with the most frequent pathogens being Salmonella spp., E. coli, and S. aureus. Two Salmonella spp. cases had evidence of AMR, and several E. coli cases were multidrug resistant (42.9%) or monoresistant (14.3%). Culture contamination was observed in 3.6% cases. Our data suggest that blood cultures should be included as a routine diagnostic test, and pre-screening patients for the most common viral infections, such as dengue, influenza and chikungunya viruses, would conserve scarce resources without negatively impacting patient benefit. The routine practice of AMR susceptibility testing on positive blood cultures in Indonesia is encouraging and should be continued to inform clinical decisions on patient treatment in real-time. The country could benefit from clear guidance at the national level, particularly regarding the timing of blood collection prior to antibiotic administration, the prioritization of patients with comorbidities, blood collection practices to reduce environmental contamination, and the supplementation of blood cultures with molecular assays to combat false-negative results. Additionally, Indonesia could greatly benefit from a nationwide program for the systematic collection and dissemination of blood culture and AMR results.
**Additional Information:**	
**Question**	**Response**
**Financial Disclosure**	This project has been funded in whole or in part with MOH Indonesia and Federal funds from the National Institute of Allergy and Infectious Diseases, National Institutes

Enter a financial disclosure statement that describes the sources of funding for the work included in this submission. Review the submission guidelines for detailed requirements. View published research articles from *PLOS ONE* for specific examples.<br><br>This statement is required for submission and **will appear in the published article** if the submission is accepted. Please make sure it is accurate.<br><br>**Unfunded studies**<br>Enter: *The author(s) received no specific funding for this work.*<br><br>**Funded studies**<br>Enter a statement with the following details:<br>• Initials of the authors who received each award<br>• Grant numbers awarded to each author<br>• The full name of each funder<br>• URL of each funder website<br>• Did the sponsors or funders play any role in the study design, data collection and analysis, decision to publish, or preparation of the manuscript?<br>• **NO** - Include this sentence at the end of your statement: *The funders had no role in study design, data collection and analysis, decision to publish, or preparation of the manuscript.*<br>• **YES** - Specify the role(s) played.<br><br>* typeset	of Health, under contract Nos. HHSN261200800001E and HHSN261201500003I. The content of this publication does not necessarily reflect the views or policies of the Department of Health and Human Services, nor does mention of trade names, commercial products, or organizations imply endorsement by the U.S. Government.
**Competing Interests**<br><br>Use the instructions below to enter a competing interest statement for this submission. On behalf of all authors, disclose any competing interests that could be perceived to bias this work—acknowledging all financial support and any other relevant financial or non-financial competing interests.<br><br>This statement is required for submission and **will appear in the published article** if the submission is accepted. Please make	The authors have declared that no competing interests exist.

sure it is accurate and that any funding sources listed in your Funding Information later in the submission form are also declared in your Financial Disclosure statement.<br><br>View published research articles from *PLOS ONE* for specific examples.<br><br>**NO authors have competing interests**<br><br>Enter: *The authors have declared that no competing interests exist.*<br><br>**Authors with competing interests**<br><br>Enter competing interest details beginning with this statement:<br><br>*I have read the journal's policy and the authors of this manuscript have the following competing interests: [insert competing interests here]*<br><br>* typeset	
**Ethics Statement**<br><br>Enter an ethics statement for this submission. This statement is required if the study involved:<br><br>• Human participants<br>• Human specimens or tissue<br>• Vertebrate animals or cephalopods<br>• Vertebrate embryos or tissues<br>• Field research<br><br>Write "N/A" if the submission does not require an ethics statement.<br><br>General guidance is provided below. Consult the submission guidelines for detailed instructions. **Make sure that all information entered here is included in the Methods section of the manuscript.**	Ethical approvals for the AFIRE study were granted by the Institutional Review Boards of the National Institute of Health Research and Development (NIHRD), Indonesia Ministry of Health (KE.01.05/EC/407/2012), the Faculty of Medicine at the University of Indonesia and RSUPN Dr. Cipto Mangunkusumo Hospital (451/PT02.FK/ETIK/2012), and RSUD Dr. Soetomo Hospital (192/Panke.KKE/VIII/2012). All eligible patients who agreed to participate in the study provided written informed consent before enrollment.

**Format for specific study types**<br><br>**Human Subject Research (involving human participants and/or tissue)**<br>• Give the name of the institutional review board or ethics committee that approved the study<br>• Include the approval number and/or a statement indicating approval of this research<br>• Indicate the form of consent obtained (written/oral) or the reason that consent was not obtained (e.g. the data were analyzed anonymously)<br><br>**Animal Research (involving vertebrate animals, embryos or tissues)**<br>• Provide the name of the Institutional Animal Care and Use Committee (IACUC) or other relevant ethics board that reviewed the study protocol, and indicate whether they approved this research or granted a formal waiver of ethical approval<br>• Include an approval number if one was obtained<br>• If the study involved *non-human primates*, add *additional details* about animal welfare and steps taken to ameliorate suffering<br>• If anesthesia, euthanasia, or any kind of animal sacrifice is part of the study, include briefly which substances and/or methods were applied<br><br>**Field Research**<br><br>Include the following details if this study involves the collection of plant, animal, or other materials from a natural setting:<br>• Field permit number<br>• Name of the institution or relevant body that granted permission	
**Data Availability**<br><br>Authors are required to make all data underlying the findings described fully available, without restriction, and from the time of publication. PLOS allows rare exceptions to address legal and ethical concerns. See the PLOS Data Policy and FAQ for detailed information.	Yes - all data are fully available without restriction

A Data Availability Statement describing where the data can be found is required at submission. Your answers to this question constitute the Data Availability Statement and **will be published in the article**, if accepted.<br><br>**Important:** Stating 'data available on request from the author' is not sufficient. If your data are only available upon request, select 'No' for the first question and explain your exceptional situation in the text box.<br><br>Do the authors confirm that all data underlying the findings described in their manuscript are fully available without restriction?	
**Describe where the data may be found in full sentences. If you are copying our sample text, replace any instances of XXX with the appropriate details.**<br><br>• If the data are **held or will be held in a public repository**, include URLs, accession numbers or DOIs. If this information will only be available after acceptance, indicate this by ticking the box below. For example: *All XXX files are available from the XXX database (accession number(s) XXX, XXX.).*<br>• If the data are all contained **within the manuscript and/or Supporting Information files**, enter the following: *All relevant data are within the manuscript and its Supporting Information files.*<br>• If neither of these applies but you are able to provide **details of access elsewhere**, with or without limitations, please do so. For example:<br><br>*Data cannot be shared publicly because of [XXX]. Data are available from the XXX Institutional Data Access / Ethics Committee (contact via XXX) for researchers who meet the criteria for access to confidential data.*<br><br>*The data underlying the results presented in the study are available from (include the name of the third party*	All relevant data are within the manuscript and its Supporting Information files.

and contact information or URL).<br>• This text is appropriate if the data are owned by a third party and authors do not have permission to share the data.<br><br>* typeset	
Additional data availability information:	

Jakarta, 14th July 2022

Editor PLOS ONE,

Please find enclosed the revised manuscript entitled “The Characteristics of Bacteremia Among Patients with Acute Febrile Illness Requiring Hospitalization in Indonesia” for publication in PLOS ONE.

We sincerely thank the Reviewers for reviewing our manuscript and for the suggestions that we received. We have made a concerted effort to adequately respond to each suggestion received from the Reviewers. We firmly believe that the Reviewers’ comments and suggestions have significantly improved this manuscript.

Thank you very much.

Best regards,
Herman Kosasih

Manuscript
Click here to access/download;Manuscript;Blood Culture Manuscript_PLOS One_rev - clean.docx

The Characteristics of Bacteremia among Patients with Acute Febrile Illness Requiring Hospitalization in Indonesia

Pratiwi Soedarmono[1], Aly Diana[2,3], Patricia Tauran[4], Dewi Lokida[5], Abu Tholib Aman[6], Bachti Alisjahbana[7], Dona Arlinda[8], Emiliana Tjitra[8], Herman Kosasih[2*], Ketut Tuti Parwati Merati[9], Mansyur Arif[4], Muhammad Hussein Gasem[10], Nugroho Harry Susanto[2], Nurhayati Lukman[2], Retna Indah Sugiyono[2], Usman Hadi[11], Vivi Lisdawati[12], Karine G Fouth Tchos[13], Aaron Neal[13], Muhammad Karyana[2,8]

[1] Faculty of Medicine, Universitas Indonesia/ Dr. Cipto Mangunkusumo Hospital, Jakarta, Indonesia

[2] Indonesia Research Partnership on Infectious Disease (INA-RESPOND), Jakarta, Indonesia

[3] Department of Public Health, Faculty of Medicine, Universitas Padjadjaran, Sumedang, Indonesia

[4] Faculty of Medicine, Universitas Hasanuddin/ Dr. Wahidin Sudirohusodo Hospital, Makassar, Indonesia

[5] Tangerang District Hospital, Tangerang, Banten, Indonesia

[6] Faculty of Medicine, Public Heath, and Nursing, Universitas Gadjah Mada/ Dr. Sardjito Hospital, Yogyakarta, Indonesia

[7] Department of Internal Medicine, Faculty of Medicine, Universitas Padjadjaran/ Dr Hasan Sadikin Hospital, Bandung, Indonesia

[8] National Institute of Health Research and Development (NIHRD), Ministry of Health Republic of Indonesia, Jakarta, Indonesia.

[9] Faculty of Medicine, Udayana University, Sanglah General Hospital, Denpasar, Bali, Indonesia

[10] Faculty of Medicine, Diponegoro University/ Dr. Kariadi Hospital, Semarang, Indonesia

[11] Faculty of Medicine, Universitas Airlangga/ Dr. Soetomo Hospital, Surabaya, Indonesia

[12] Sulianti Saroso Infectious Disease Hospital, Jakarta, Indonesia

[13] National Institute of Allergy and Infectious Disease (NIAID), National Institutes of Health, Bethesda, Maryland, United States of America

* Corresponding author

E-mail: hkosasih@ina-respond.net (HK)

Abstract

Blood culturing remains the "gold standard" for bloodstream infection (BSI) diagnosis, but the method is inaccessible to many developing countries due to high costs and insufficient resources. To better understand the utility of blood cultures among patients in Indonesia, a country where blood cultures are not routinely performed, we evaluated data from a previous cohort study that included blood cultures for all participants. An acute febrile illness study was conducted from July 2013 to June 2016 at eight major hospitals in seven provincial capitals in Indonesia. All participants presented with a fever, and two-sided aerobic blood cultures were performed within 48 hours of hospital admission. Positive cultures were further assessed for antimicrobial resistance (AMR) patterns. Specimens from participants with negative culture results were screened by advanced molecular and serological methods for evidence of causal pathogens. Blood cultures were performed for 1,459 of 1,464 participants, and the 70.6% (1,030) participants that were negative by dengue NS1 antigen test were included in further analysis. Bacteremia was observed in 8.9% (92) participants, with the most frequent pathogens being *Salmonella spp.* (51), *Escherichia coli* (14), and *Staphylococcus aureus* (10). Two *Salmonella spp.* cases had ev[illegible] and several *E. coli* cases were multidrug resistant (42.9%, 6/14) or monoresistant (14.3%, 2/14). Culture contamination was observed in 3.6% (37) cases. Advanced laboratory assays identified ~~culturable pathogens~~ in participants having negative cultures, with 23.1% to 90% of cases being missed by blood cultures. Blood cultures are a valuable diagnostic tool for hospitalized patients presenting with fever. In Indonesia, pre-screening patients for the most common viral infections, such as dengue, influenza, and chikungunya viruses, would

which serovar was it S.Typhi? mention

etiological agent

mention names of test or assays

maximize the benefit to the patient while also conserving resources. Blood cultures should also be supplemented with advanced laboratory tests when available.

Introduction

Bloodstream infections (BSI) [1] are a significant cause of morbidity and mortality in both developing and developed countries [2–4]. The "gold standard" method for BSI diagnosis remains blood culturing [5–7], a straightforward laboratory technique that is inaccessible to many developing countries due to high costs and insufficient resources. Blood cultures provide both definitive microbiological evidence of infection and serve as a crucial tool to monitor the serious global health threat of antimicrobial resistance (AMR) [8]. The threat of AMR further exacerbates the burden felt in countries without routine access to this diagnostic method, including in Indonesia, and allows AMR to continue threatening populations worldwide. The early and accurate identification of causative microorganisms and their susceptibility to antibiotics is essential to improve patient survival and prevent emerging AMR pathogens.

Even with access to routine blood cultures, the interpretation of results can be challenging and should align with clinical observations. Bacterial growth is a consequence of the initial quantity of bacteria in the specimen, the quality of the specimen, the timing of specimen collection with clinical treatment, and the biological nature of the bacteria. Negative blood cultures alone are not definitive for diagnosis, as advanced laboratory methods often detect missed culturable organisms from the same specimen types [9,10]. Routine analysis of specimens can be impacted by contamination from the environment of

the patient [11,12]. In most settings, only 5 to 13% of blood cultures will become positive, and of those, 20–56% result from contamination [7,13–16].

In Indonesia, acute febrile illness resulting from BSIs remains a common cause of hospitalization, morbidity, and mortality. Although infectious diseases are the primary cause of hospitalization in the country, clinicians do not routinely perform blood cultures as part of standard clinical care [17]. When clinicians perform blood cultures, generally in severely ill patients referred to tertiary care, they do not consistently use best laboratory practices [18]. Data on blood culture use, performance, and contamination rates in Indonesia remain very limited [17,19,20]. Consequently, data on the emergence and spread of AMR pathogens in the country is unreliable and incomplete, complicating antibiotic stewardship efforts in the region.

The epidemiology of pathogens associated with fever in Indonesia is not well understood, as public health surveillance data is limited and only a few local studies have been conducted [19,21–26]. Among published studies, dengue virus, chikungunya virus, influenza virus, *Salmonella* Typhi, *Rickettsia spp.*, and *Leptospira spp.* are consistently the most common causes of acute febrile illness hospitalizations. A study in Papua from November 1997 to February 2000 enrolled 226 hospitalized patients that were negative for malaria, the majority of whom were determined to have typhoid fever (18%), leptospirosis (12%), rickettsioses (8%), and dengue fever (7%) [23]. An observational fever study in Bandung identified dengue virus in 12.4% of fever episodes, followed by *S.* Typhi (7.4%), and chikungunya virus (7.1%) [24,26,27]. A 2005-2006 study in Semarang found rickettsioses and leptospirosis in 7% and 10%, respectively, of 137 acute undifferentiated fever cases [21]. The parent study of the research presented here found the most prevalent pathogens among participants at eight hospitals in 7 major cities in Indonesia to be dengue virus (27-

52%), *Rickettsia spp.* (2-12%), *S.* Typhi (0.9-13%), influenza virus (2-6%), *Leptospira spp.* (0-5%), and chikungunya virus (0-4%) [19].

To better understand the utility of blood cultures among patients with acute febrile illness in Indonesia, we evaluated data from a previously published multicenter observational prospective cohort study conducted across the country [19]. Gaining insight into pathogens commonly identified by blood culture, contamination rates, AMR patterns, and disease outcomes will provide actionable evidence to support decision making for Indonesia's national blood culture testing policy.

Methods

Study design and sample collection

A prospective observational study enrolling febrile patients who required hospitalization was conducted by the Indonesia Research Partnership on Infectious Disease (INA-RESPOND) from July 2013 to June 2016 at eight major hospitals in seven provincial capitals in Indonesia: Dr. Cipto Mangunkusumo Hospital in Jakarta, Sulianti Saroso Infectious Disease Hospital in Jakarta, Dr. Wahidin Sudirohusodo Hospital in Makassar, Dr. Sardjito Hospital in Yogyakarta, Dr. Hasan Sadikin Hospital in Bandung, Sanglah General Hospital in Denpasar, Dr. Soetomo Hospital in Surabaya, and Dr. Kariadi Hospital, in Semarang. The full details of this study, known as AFIRE, were published previously [19]. Briefly, inclusion criteria consisted of axillary body temperature ≥38˚C, ≥1 year of age, and hospitalization within the past 24 hours. Patients were excluded from the study if they had subjective fever for ≥14 days or were hospitalized in the last 3 months. Demographic, clinical, and laboratory data, including hematology results, were collected at baseline, once during days 14–28, and

three months after enrollment. Blood and other biological specimens were collected at each study visit.

During the baseline visit, blood was collected for cultures, clinically relevant rapid diagnostic tests when available, and dengue virus rapid diagnostic tests. Dengue virus infection remains a significant burden across Indonesia [28,29], with disease incidence increasing in recent years [30]. Though other viral agents are present in Indonesia, none are as prevalent as dengue virus [24,31], and most are challenging to diagnose due to limitations with available rapid diagnostic tests [32,33]. Given the widespread prevalence of dengue virus infection, and the very high specificity (almost 100%) and good sensitivity (70-87%) of NS1 antigen rapid diagnostic tests [34], we employed universal dengue virus screening to rapidly resolve the unknown etiologies of study participants. Participants with negative NS1 antigen tests were further considered for BSIs through blood culture tests and other etiologies, as determined through advanced testing at the INA-RESPOND reference laboratory.

Laboratory tests

Aerobic blood cultures were performed within 48 hours of a participant being admitted to the emergency department of a study site. Blood volumes of approximately 5-8 mL for adults and 1-3 mL for pediatrics were collected from each arm, whenever possible, directly into separate aerobic blood culture bottles. If blood could not be collected from each arm due to clinical reasons, blood was collected from a single arm for a single aerobic blood culture bottle. Study physicians were advised to delay the administration of IV antibiotics until blood specimens were collected, provided that there were no immediate

risks to the participant. Each hospital performed a complete blood count (CBC) as part of standard-of-care procedures during enrollment.

Inoculated aerobic blood culture bottles were incubated using a continuous-monitoring blood culture system, either BACTEC (Becton-Dickinson, Sparks, Maryland) or BacT/Alert (bioMérieux, Inc., Durham, North Carolina) [35]. Manufacturer guidelines were followed for all bacterial cultures, and automated growth identification systems, either BD Phoenix (Becton Dickinson) or VITEK 2 (bioMérieux, Inc., Durham, North Carolina), were used for bacterial identification and antibiotic susceptibility testing. Blood cultures were performed and analyzed at the hospitals' nationally accredited clinical laboratories by trained, certified staff. All instruments and standards were calibrated appropriately according to manufacturer guidelines, and all tests were run alongside appropriate positive and negative control to ensure the integrity and accuracy of the results. Organism identification was considered acceptable when the confidence level in the automated growth identification system was ≥95% probability [36]. Quality control tests were performed weekly at all site laboratories, and each new lot of ID cards was tested using validated stocks of culture organisms.

Growth observed in blood cultures was classified as either "true positive" or "false positive." True positives included pathogenic bacterial species, particularly those identified as priority pathogens by the World Health Organization Global Antimicrobial Resistance and Use Surveillance System (WHO GLASS) [37], observed in at least one blood culture. Additionally, non-WHO GLASS pathogens found in either one or both cultures and being consistent with clinical manifestations were also considered to be true positives. False positives included growth of bacteria and fungi which were not clinically relevant and growth of known culture contaminants. Bacterial culture contamination was defined as any

culture growing viridans group streptococci, *Corynebacterium spp.*, *Bacillus spp.*, *Diphtheroid spp.*, *Micrococcus spp.*, *Propionibacterium spp.*, and coagulase-negative staphylococci [12].

At the INA-RESPOND reference laboratory, specimens from all participants were screened for dengue using NS1 antigen ELISA, dengue RT-PCR, and dengue IgM and IgG. Molecular tests in acute specimens and serological tests in acute and convalescent specimens were performed to detect bacterial infections such as *S.* Typhi, *S*. Paratyphi, *Leptospira spp.*, and *Rickettsia typhi*, and viruses such as influenza, chikungunya, and measles. Details of diagnostic assays for this study were previously described [19].

Statistical analysis

Data were collected in OpenClinica (OpenClinica LLC, MA, USA) and analyzed using STATA v.15.1 (StataCorp LLC, TX, USA). Proportions were compared between categorical variables using Pearson's chi-squared test. The student's t-test was used to assess continuous variables. All p-values were two-sided with a significance level set to $p<0.05$.

Ethical clearance

Ethical approvals for the AFIRE study were granted by the Institutional Review Boards of the National Institute of Health Research and Development (NIHRD), Indonesia Ministry of Health (KE.01.05/EC/407/2012, dated 23 May 2012), the Faculty of Medicine at the University of Indonesia and RSUPN Dr. Cipto Mangunkusumo Hospital (451/PT02.FK/ETIK/2012, dated 23 July 2012), and RSUD Dr. Soetomo Hospital

(192/Panke.KKE/VIII/2012, dated 13 August 2012). All eligible patients who agreed to participate in the study provided written informed consent before enrollment.

Results

A total of 1,464 participants were enrolled in the AFIRE study, and aerobic blood cultures were performed for 1,459 participants (Fig 1). The remaining 5 participants had insufficient blood specimens for following reasons: 1 adult was in a severe condition (decreased of consciousness), 2 participants (1 child and 1 adult) self-discharged against medical advice, and the guardians of 2 children refused to allow more blood to be drawn. Bacterial growth was observed for 10.3% (150) participants, including 56.0% (84) with WHO GLASS pathogens, 5.3% (8) with other non-WHO GLASS pathogens, and 38.7% (58) with false positives. No growth was observed for 89.7% (1,309) participants. All participants were screened for dengue virus by NS1 antigen and dengue IgM/IgG antibody tests, resulting in 29.4% (429) positive results, 415 from “No Growth” and 14 from the “False Positive” group. The remaining 70.6% (1,030) dengue-negative participants were included in this analysis.

Fig 1. General blood culture results observed among study participants. Participants provided blood from either one or both arms for aerobic blood cultures, and bacterial growth was observed from either one or both sides. All participants providing blood underwent screening for dengue virus infection by NS1 antigen test.

Results of blood cultures: community-acquired infection (CAI)

Bacteremia was observed in 8.9% (92) of the 1,030 dengue-negative participants, with the most frequent pathogens being *Salmonella spp.* in 51 participants, *Escherichia coli* in 14 participants, and *Staphylococcus aureus* in 10 participants (Table 1). Dengue-negative false positive results were observed in 4.3% (44) participants, with the most frequent microorganism being contaminating coagulase-negative *Staphylococcus spp.* in 32 participants. From the 136 dengue-negative participants with any microbial growth, 97.8% (133) had blood collected from two sides of the body (Fig 1). Growth from both sides was observed in 58.7% of participants with true positive results and 25.0% of participants with false positive results.

Table 1. Specific blood culture results among dengue-negative study participants.

	Pathogen	Positive Results	Percent of Positive Results Within Group
WHO GLASS Priority Pathogens (N = 84)	*Salmonella spp.*	51	60.7
	Escherichia coli	14	16.7
	Staphylococcus aureus	10	11.9
	Klebsiella pneumoniae	5	6.0
	Acinetobacter spp.	2	2.4
	Streptococcus pneumoniae	2	2.4
Non-WHO GLASS Pathogens (N = 8)	*Pseudomonas aeruginosa*	2	25.0
	Staphylococcus hominis ssp. hominis	1	12.5
	Enterobacter aerogenes	1	12.5
	Enterococcus faecalis	1	12.5
	Pseudomonas cepacea	1	12.5

	Pseudomonas spp.	1	12.5
	Streptococcus pyogenes	1	12.5
Clinically Irrelevant Growth (N = 7)	*Pantoea spp.*	2	28.6
	Sphingomonas paucimobilis	2	28.6
	Alcaligenes faecalis	1	14.3
	Candida pelliculosa	1	14.3
	Rhizobium radiobacter	1	14.3
Contaminants (N = 37)	*Coagulase-Negative Staphylococcus*	32	86.5
	Bacillus spp.	2	5.4
	Micrococcus luteus	1	2.7
	Kocuria spp.	1	2.7
	Streptococcus viridans	1	2.7
No Growth (N = 894)	*None*	0	0.0

Since *Salmonella spp.* were found in over half (55.4%) of true positives (Table 1), participants with true positive results were analyzed in either *Salmonella spp.* or non-*Salmonella spp.* groups (Table 2). Participant demographics revealed nearly equal numbers of male and female participants in the study, with equal numbers of true positive cases in the two groups. Participants in the *Salmonella spp.* group were significantly younger, with a median age of 14 years old, compared to non-*Salmonella spp.* and false positive groups, with median ages of 44 years old and 24.6 years old, respectively. Over 62.7% of *Salmonella spp.* cases were in participants ≤18 years old, while only 26.8% of non-*Salmonella spp.* cases

were in this same age range. There were no significant differences between all groups in the days of onset before hospitalization or the length of hospitalization.

Table 2. Participant characteristics, hematology results, and mortality.

	**True Positive (92)**		**False Positive and No Growth (938)**	**Total (1,030)**
	***Salmonella spp.* (51)**	**Non-*Salmonella spp.* (41)**		
Male, N (%)	29 (56.9)	17 (41.5)	502 (53.9)	553 (53.7)
Median age, years (range, IQR)	14 (2.5-54, 14.7)	44 (1-84, 40.0)	24.6 (1-92, 36.5)	24 (1-92, 36.2)
Mean age, years (SD)	16.2 (11.1)[D,E]	39.6 (24.0)[D,F]	28.6 (21.4)[E,F]	28.5 (21.4)
Distribution of cases by age group, N (%)				
1-5 years	4 (7.8)	5 (12.2)	154 (16.4)	163 (15.8)
>5-18 years	28 (54.9)[D,E]	6 (14.6)[D]	184 (19.6)[E]	218 (21.2)
>18-45 years	18 (35.3)	11 (26.8)	365 (38.9)	394 (38.3)
>45-65 years	1 (2.0)[D,E]	13 (31.7)[C,D]	179 (19.1)[C,E]	193 (18.7)
>65 years	0 (0.0)[B,D]	6 (14.6)[C,D]	56 (6.0)[B,C]	62 (6.0)
Days of onset before hospitalization, median (range, IQR)	7 (1-13, 4)	4 (1-15, 4)	4 (1-15, 4)	4 (1-15, 4)
Length of hospitalization, median (range, IQR)	7 (2-38, 4)	8 (2-40, 7)	6 (1-55, 3.3)	6 (1-55, 4)
Received intravenous antibiotics prior to blood collection, N (%)	9 (17.6)[A,E]	16 (39.0)[A]	389 (41.5)[E]	414 (40.2)
Received any antibiotics following blood collection, N (%)	31/31 (100)[E]	18/18 (100)[A,C]	199/269 (74.0)[A,C,E]	248/318 (77.9)
Hematology at enrollment, N (%)				
Leukopenia	13/51 (25.5)[E]	5/41 (12.2)	120/937 (12.8)[E]	138/1029 (13.4)

Normal Leukocyte	35/51 (68.6)[A,E]	19/41 (46.3)[A]	462/937 (49.3)[E]	516/1029 (50.1)
Leukocytosis	3/51 (5.9)[D,E]	17/41 (41.5)[D]	355/937 (37.9)[E]	375/1029 (36.4)
Lymphopenia	16/44 (36.4)[B,D]	26/38 (68.4)[D]	442/810 (54.6)[B]	484/892 (54.3)
Normal Lymphocyte	17/44 (38.6)[A]	7/38 (18.4)[A,C]	285/810 (35.2)[C]	309/892 (34.6)
Lymphocytosis	11/44 (25.0)[E]	5/38 (13.2)	83/810 (10.2)[E]	99/892 (11.1)

Outcome, N (%)				
Died	3 (5.9)[D]	11 (26.8)[D,F]	69 (7.4)[F]	83 (8.1)

Study participants with true positive culture results were sub-categorized into *Salmonella spp.* and non-*Salmonella spp.* groups to better resolve analyses. Comparisons for significance occur across column groups only.
A,B,C indicates p-value <0.05
D,E,F indicates p-value <0.01

Intravenous antibiotics were administered prior to blood collection significantly less frequently in the *Salmonella spp*. group (17.6%, 9/51) compared to other groups (Table 2). All participants with true positive results were administered antibiotics following blood collection, and 74% of participants with false positive results received antibiotics. Hematology profiles at enrollment differed significantly between the *Salmonella spp.* and non-*Salmonella spp.* groups. Leukopenia and normal leukocyte counts were observed in 94.1% (48) of *Salmonella spp.* cases compared to 58.5% (24) of non-*Salmonella spp.* cases and 62.0% (582) of false positive and no growth cases. Similarly, leukocytosis was significantly lower in the *Salmonella spp.* group compared to the other groups. Lymphopenia was observed in 36.4% (16) of the *Salmonella spp.* cases, which is significantly lower than the 68.4% (26) non-*Salmonella spp.* cases and the 54.6% (442) false positive and no growth cases. Mortality was significantly higher in the non-*Salmonella spp.* group compared to the other groups.

Cases of true positives were distributed across age groups and study sites (Table 3). While *Salmonella spp.* were most frequently found in pediatrics (62.7% of cases), *E. coli*, *S. aureus*, and *K. pneumoniae* were most frequently found in adults (85.7%, 80.0%, and 80.0% of cases, respectively). Most *Salmonella spp.* cases were seen in Bandung (BDG, 41.2%), Semarang (SMG, 23.5%), and Surabaya (SUB, 21.6%). This differed significantly from cases seen in Makassar (MKS, 9.8%), Yogyakarta (YOG, 2.0%), Denpasar (DPS, 2.0%), and Jakarta (JKT, 0.0%). Other than *Salmonella spp.*, there were no significant differences in the distribution of pathogens across study sites, likely due to the low numbers of cases.

Table 3. Positive blood culture pathogens by participant age group and study location.

Pathogen Identified	Age group (years old)					Location							Total
	≥1-5	>5-18	>18-45	>45-65	>65	Bdg	Sby	Smr	Dps	Mks	Yog	Jkt	
Salmonella spp.	4	28 (1[†])	18 (1[†])	1 (1[†])	0	21	11	12	1	5	1	0	51
Escherichia coli	1	1	3 (1[†])	5 (1[†])	4	3	3	0	4	0	3	1	14
Staphylococcus aureus	0	2	4 (1[†])	4 (1[†])	0	1	1	3	2	1	0	2	10
Klebsiella pneumoniae	0	1	0	3 (2[†])	1	0	1	0	2	2	0	0	5
Acinetobacter spp.	0	1	1	0	0	0	0	0	1	0	0	1	2
Enterobacter aerogenes	0	0	1 (1[†])	0	0	0	0	0	1	0	0	0	1
Enterococcus faecalis	1	0	0	0	0	0	0	0	0	0	0	1	1
Pseudomonas aeruginosa	1	0	1 (1[†])	0	0	0	0	0	0	0	2	0	2
Pseudomonas cepacea	0	0	0	0	1	1	0	0	0	0	0	0	1
Pseudomonas species	0	0	1	0	0	0	1	0	0	0	0	0	1

Streptococcus pneumoniae	1 (1†)	1 (1†)	0	0	0	1	0	0	0	1	0	0	2
Streptococcus pyogenes	0	0	0	1	0	0	0	0	1	0	0	0	1
Staphylococcus hominis ssp hominis	1 (1†)	0	0	0	0	0	0	0	0	0	1	0	1
**Total**	9 (2†)	34 (2†)	29 (5†)	14 (5†)	6	27	17	15	12	9	7	5	92

† Indicates study participants who died

Bdg: Bandung; Sby: Surabaya; Smr: Semarang; Dps: Denpasar; Mks: Makassar; Yog: Yogyakarta; Jkt: Jakarta

The 938 participants in the false positive and no growth groups had specimens screened by other laboratory methods to determine potential etiologies (Table 4). PCR on blood specimens identified etiologies in 168 participants, serology identified etiologies in 220 participants, and other methods identified etiologies in 94 participants. Among the culturable bacterial pathogens identified in these groups were the WHO GLASS pathogens *S.* Typhi (51), *S. pneumoniae* (18), *K. pneumoniae* (8), *A. baumanii* (7), *E. coli* (7), and *S. aureus* (3). When combined with the culture results from the WHO GLASS priority pathogens group in Table 1, 50% of *S.* Typhi cases, 33.3% of *E. coli* cases, 23.1% of *S. aureus* cases, 61.5% of *K. pneumoniae* cases, 77.8% of *Acinetobacter spp.* cases, and 90% of *S. pneumoniae* cases in the AFIRE study [19] were not identified by blood cultures.

Table 4. Pathogens detected by molecular, serological, or other laboratory methods from participants with false positive and no growth blood cultures.

False Positive and No Growth (N=938)		Confirmatory Methods		
**Pathogen**	**N**	**Blood PCR**	**Serology**	**Other Methods**

Rickettsia typhi	101	65	36	
Influenza	66	0	59	7: Sputum PCR
Salmonella Typhi	51	3	48	
Leptospira spp.	44	31	13	
Chikungunya	38	30	8	
Dengue	35	0	35	
Mycobacterium tuberculosis	20	0	0	20: Sputum Microscopy
Streptococcus pneumoniae	18	10	0	8: Sputum PCR
Measles	14	9	5	
Amoeba	11	0	0	11: Feces Microscopy
RSV	11	0	9	2: Swab PCR
HHV-6	9	9	0	
Klebsiella pneumoniae	8	1	0	5: Sputum Culture<br>2: Swab Culture
Acinetobacter baumanii	7	1	0	4: Sputum PCR<br>1: Swab PCR<br>1: Urine PCR
Escherichia coli	7	1	0	4: Urine Culture<br>2: Pus Culture
Hepatitis A	6	0	6	
Pseudomonas aeruginosa	6	0	0	4: Sputum Culture<br>2: Urine Culture
Enterococcus faecalis	3	0	0	2: Pus Culture<br>1: Urine Culture
Staphylococcus aureus	3	0	0	3: Pus Culture
Mycobacterium leprae	2	0	0	2: Skin Microscopy
Plasmodium spp.	2	0	0	2: Rapid Antigen Test
Seoul virus	2	2	0	
Adenovirus	1	1	0	
Ascaris lumbricoides	1	0	0	1: Feces Microscopy
Ascaris lumbricoides and *Trichuris Trichiura*	1	0	0	1: Feces Microscopy
Bordetella pertussis and *Streptococcus pneumoniae*	1	0	0	1: Sputum PCR
HCoV-OC43	1	1	0	
Enterobacter aerogenes	1	0	0	1: Sputum Culture

Enterobacter cloacae	1	0	0	1: Sputum Culture and PCR
Enterococcus avium	1	0	0	1: Pus Culture
Enterovirus	1	1	0	
EPEC	1	0	0	1: Feces Culture
HIV	1	1	0	
Metapneumovirus	1	0	0	1: Swab PCR
Moraxella catarrhalis and Influenza B	1	0	0	1: Sputum Culture and PCR
Mycoplasma pneumoniae	1	0	0	1: Sputum PCR
Norovirus II	1	1	0	
Rickettsia felis	1	1	0	
Rubella	1	0	1	
Streptococcus faecalis	1	0	0	1: Urine Culture
Unknown	456	0	0	
**Total**	**938**	**168**	**220**	**94**

Plasma, serum, and clinically relevant specimens were collected from all study participants and tested in a central lab for culturable and non-culturable pathogens based on a standard study algorithm and clinical suspicion.

Antimicrobial resistance patterns

Antimicrobial resistance patterns were observed in several participants with blood cultures positive for WHO GLASS priority pathogens (Fig 2). Among the 51 *Salmonella spp.* cases, evidence of multidrug resistance was observed in one participant and monoresistance in one participant. In contrast, *E. coli* cases were mostly multidrug resistant (42.9%, 6/14) or monoresistant (14.3%, 2/14), with observed resistances to ampicillin (87.5%, 7/8), co-trimoxazole (60.0%, 3/5), ceftriaxone (45.4%, 5/11), ceftazidime (41.6%, 5/12), cefotaxime (37.5%, 3/8), cefepime (33.3%, 2/6), ciprofloxacin (30.0%, 3/10), and levofloxacin (25.0%, 2/8). Two participants (JOG-A and DPS-A) receiving ceftriaxone died before their antimicrobial resistance test results, and one participant (JOG-B) survived when switched from ceftazidime to ciprofloxacin based on their test results.

Figure 2. Antimicrobial resistance patterns observed in WHO GLASS priority pathogens from true positive blood cultures. Participants with resistant (R) infections are identified by study location, and participants with sensitive (S) infections or infections with no testing data (ND) are grouped into Other or No Data categories.

Methicillin-resistant *S. aureus* (MRSA) was observed in one participant based on oxacillin susceptibility testing, and two participants with oxacillin-sensitive *S. aureus* infections died. Both participants with *S. pneumoniae* bacteremia died, though antimicrobial resistance was only observed in one of the participants. All cases of *Acinetobacter spp.* and *K. pneumoniae* that underwent drug sensitivity testing were sensitive to antibiotics.

Disease outcomes

Characteristics and laboratory findings of participants who died during hospitalization are shown in Table 5. A total of 83 participants in this analysis died during hospitalization. Among these, 16.9% (14) had true positive blood cultures (Table 5A), resulting in 15.2% mortality in the true positive group. This mortality rate is twofold higher than the 7.4% mortality observed in the false positive and no growth groups. Overall mortality in the *Salmonella* spp. group (5.9%) was significantly lower than the non-*Salmonella spp.* group (26.8%). Among deceased participants, there were no significant differences in demographics between the true positive group and false positive and no growth groups. Most deceased participants had comorbidities including diabetes mellitus (4), hepatitis B (3), HIV (2), tuberculosis (2), brain tumor (1), TRALI (1), neoplasia (1), and

wchich serovar mention

others (6) (Table 5B). Antimicrobial-resistant pathogens were identified in 3 of the 14 deceased participants with true positives (Table 5). In the false positive and no growth groups, other laboratory methods such as PCR and/or serology were used to identify culturable bacterial pathogens including *S.* Typhi (2), *A. baumanii* (1), *E. avium* (1), *E. coli* (1), *M. catarrhalis* (1), and *S. pneumoniae* (1) (Table 5B).

Table 5. Participant characteristics, clinical diagnoses, and identified pathogens from fatal cases in the study.

(A) Characteristics of deceased participants categorized by blood culture growth result.

	**True Positive (14)**		**False Positive and No Growth (69)**	**Total (83)**
	***Salmonella* spp. (3)**	**Non-*Salmonella spp.* (11)**		
Male, N (%)	3 (100)	7 (63.6)	36 (52.2)	46 (55.4)
Distribution of cases by age group, N (%)				
1-5 years	0 (0.0)	2 (18.2)	4 (5.8)	6 (7.2)
>5-18 years	1 (33.3)	1 (9.1)	7 (10.1)	9 (10.8)
>18-45 years	1 (33.3)	4 (36.4)	24 (34.8)	29 (34.9)
>45-65 years	1 (33.3)	4 (36.4)	25 (36.2)	30 (36.1)
>65 years	0 (0.0)	0 (0.0)	9 (13)	9 (10.8)
Received intravenous antibiotics prior to blood collection, N (%)	1 (33.3)	1 (9.1)	34 (49.3)	36 (43.4)
Length of hospitalization, median (range, IQR)	4 (2-38)	12 (2-17)	8 (2-54)	8 (2-54)
Comorbidities, N (%)	2 (66.6)	10 (90.9)	60 (86.9)	72 (86.7)

mention serovar name

(B) Pathogens from fatal cases confirmed by blood culture or other lab methods and the accompanying clinical diagnoses, participant comorbidities, and AMR observations.

**True Positive (14)**	**Clinical Diagnosis at Death**	**Comorbidities**	**Antimicrobial Resistance**
Salmonella spp. (3)	Typhoid fever	Hepatitis B, HIV, TB	None
	Acute limb ischemia	Acute Limb Ischemia	None
	Sepsis, typhoid fever	Transfusion-Related Acute Lung Injury (TRALI)	None
Escherichia coli (2)	Cholangitis	Diabetes, Hepatitis B	Yes
	Sepsis	Anemia	Yes
Klebsiella pneumoniae (2)	UTI, diabetic ketoacidosis	Diabetes	None
	UTI	Stroke	None
Staphylococcus aureus (2)	UTI	Diabetes	None
	Sepsis	Diabetes, Chronic Kidney Disease	None
Streptococcus pneumoniae (2)	Aseptic meningitis, acute otitis media	Epilepsy	Yes
		Myelodysplasia, Hepatitis B (Cirrhosis)	None
Pseudomonas aeruginosa (1)	Stevens-Johnson syndrome	HIV, TB, Toxoplasmosis	No data
Enterobacter aerogenes (1)	Cholangitis, Sepsis	None	No data
Staphylococcus hominis ssp hominis (1)		Craniopharyngioma	None

mention serovar name

**False Positive and No Growth (69) [Confirmatory Methods]**	**Clinical Diagnosis at Death**
Mycobacterium tuberculosis (8) [GeneXpert (2), Microscopy (6)]	Pulmonary TB (3), Colitis TB and Spondylitis TB, Millar TB, HIV, Community-acquired Pneumonia, Sepsis
Rickettsia typhi (6) [PCR (6)]	Sepsis (3), Community-acquired Pneumonia, Meningoencephalitis, Diabetic Neuropathy
Influenza (3) [PCR (2), Serology (1)]	Bronchiectasis, Community-acquired Pneumonia, Sepsis

Salmonella Typhi (2)<br>[Serology (2)]	Hirschsprung's disease, HIV
Acinetobacter baumanii (1)<br>[Sputum PCR]	Community-acquired Pneumonia
Ascaris lumbricoides (1)<br>[Microscopy]	Typhoid Fever
Enterococcus avium (1)<br>[Pus culture]	Diabetic Ulcer
Escherichia coli (1)<br>[Urine culture]	UTI
HIV (1)<br>[PCR]	Sepsis
Leptospira spp. (1)<br>[PCR]	Dengue Hemorrhagic Fever I
Moraxella catarrhalis and Influenza B (1)<br>[Sputum culture and sputum PCR]	Community-acquired Pneumonia
RSV (1)<br>[Serology]	TB Pleuritis
Streptococcus pneumoniae (1)<br>[Sputum PCR]	Community-acquired Pneumonia
Unknown (41)<br>[None]	HIV (6), Sepsis (6), Community-acquired Pneumonia (9), Cellulitis (2), Cholangitis (2), Lung Abscess, Acute Leukemia, Bacterial Meningitis, Bronchitis, Cholecystitis, Chronic Myelocytic Leukemia, COPD, Diarrhea, Extrapulmonary TB, GEA, Hepatitis B, Pancytopenia, SLE, Typhoid Fever, UTI, Unknown

Discussion

BSI causes a high burden of morbidity and mortality worldwide, particularly in low- and middle-income countries (LMICs). Exact figures for BSI incidence and associated mortality in LMICs are challenging to find due to the lack of bacteriological laboratories and routine surveillance systems [38,39]. In Indonesia, very few acute febrile patients undergo aerobic blood culture testing since it is not standard practice in the healthcare system,

largely due to resource and capacity restrictions [17]. The AFIRE study presents a unique opportunity to improve our understanding of BSIs in the country since aerobic blood cultures were performed on nearly all participants, regardless of clinical suspicion of bacteremia.

Microbial growth was observed in 10.3% of all participants, with bacteremia being ultimately confirmed in 6.3% of all participants (Fig 1). These proportions are similar to previous reports, where positivity rates ranged from 10.0 - 11.4% [17]. The high prevalence of dengue fever in Indonesia often complicates the clinical assessment of acute febrile illness [25], so specimens from all participants in the AFIRE study were retrospectively tested for dengue NS1 antigen to exclude dengue as a cause of illness [19]. Data on co-infections with dengue virus and bacteremia is limited. A literature review of published case reports and studies from January 1943 to March 2016 found 3 studies in Singapore and Taiwan reporting concurrent bacteremia in 0.18-7% of dengue fever cases [40–42]. A concurrent dengue virus and *S.* Typhi case was also reported from Bandung, Indonesia [43]. In all of these studies, blood was collected for bacterial culture because patients did not improve clinically a few days to a week after dengue fever was diagnosed. Furthermore, in the majority of cases, dengue virus infection was confirmed by serology only (IgM detected or four-fold IgG increase). These reports support our finding that simultaneous infection with bacteria and dengue virus is rare. In our study, bacterial growth observed in 14 participants with positive dengue NS1 antigen tests were considered false positive blood cultures (5 *Staphylococcus hominis*, 4 *Staphylococcus epidermidis*, 1 *Kocuria rosea*, 1 *Micrococcus aureus*, 1 *Staphylococcus arlettae*, 1 coagulase-negative *Staphylococcus spp.*, and 1 *Staphylococcus waneri*).

Among dengue-negative participants with any microbial growth, 97.8% had blood cultures performed from two sides of collection. One-sided blood culture lacks sufficient sensitivity for BSI detection [44], and two-sided cultures make it easier to distinguish true bacteremia and contamination [44,45]. It has been demonstrated that collecting two or more blood culture sets, each comprising two bottles, over twenty-four hours will detect over 94% of bacteremia episodes, compared to a detection rate of only 73% with the first blood culture [44]. In many developing countries, collecting multiple blood culture sets is generally not feasible, but the minimum practice of a single, one-sided blood culture still has value if clinical care teams understand its limitations. Our data suggest that, in situations where a single, one-sided blood culture is performed, the likelihood of missing a case of bacteremia is 39% (35/89) (8.9% (89/1000) vs 5.4% (54/1000) (Fig 1). Indonesian clinicians should consider this reduced sensitivity when acting on culture results.

The reliability and interpretation of blood culture results is significantly affected by both contamination rates and the use of antibiotics prior to blood collection. General target rates for culture contamination have been set at 3% [45], and in our study we observed an overall contamination rate of 3.6%. These findings are consistent with previous reports, including a 2010-2013 study at Sardjito Hospital in Yogyakarta that found a contamination rate of 4.1% in children at the pediatric ICU and in pediatric wards [46]. Additional reports from rural Thailand and Taiwan found contamination rates ranging from 4.1-6.1% and 2.6%, respectively [47,48]. The proportion of participants who were given intravenous antibiotics prior to blood collection in our study was high (40.2%), and this may alter the blood culture results considerably [49,50]. In Indonesia, antibiotic therapy is often initiated preemptively and without confirmatory testing in an attempt to maximize positive clinical outcomes [51]. This broad use of antibiotics likely masks the true prevalence of bacteremia and may have

negative consequences for patients who subsequently appear to have no infection. Among participants with false positives or no growth, 111 had culturable microbes confirmed by other methods (Table 4), 7 of which died (Table 5). 56.8% of these overall participants received antibiotics prior to blood collection. The expansion of molecular methods would significantly help to tackle this problem, as nucleic acid probe and amplification tests have been shown to significantly improve the speed and accuracy of results in blood stream infections even after antibiotic use [52,53].

White blood cell counts, particularly leukopenia and leukocytosis, have been used to predict blood culture results. However, the accuracy of systemic inflammatory response syndrome (SIRS) criteria [54], Shapiro criteria [55], and the quick Sequential Organ Failure Assessment (qSOFA) score [56] could not be confirmed in our study. This is primarily due to the significant difference in leukocyte profiles between participants with *Salmonella spp.* versus non-*Salmonella spp*. infections. Our study suggests, as proposed by Ombelet [57] and Seigel [58] that leukocytosis should not be used as a predictor for positive blood cultures in *S. enterica*-endemic areas.

We found that *Salmonella spp.* infection was the most common community-acquired BSI (Table 1) at 55.4% of cases, which al[illegible]udies conducted in limited-resource environments [46,47]. The majority of *Salmonella* bacteremia was in pediatrics, which is consistent with a previous report from a blood culture study in Jakarta where the incidence rate of typhoid fever was higher in the 2-15 year age group, with a mean age of onset of 10.2 years [59]. This commonly observed age association may be due to poor hygiene practices or the consumption of foods, particularly street food, outside of the home [60]. Though over half of bacteremia cases were due to *Salmonella spp.* infection, only 21.4% of bacteremia deaths were due to the pathogen. Among these fatal cases, all had

mention serovar name

significant comorbidities, suggesting that patients with multiple comorbidities would benefit from prioritization of blood culture diagnostics.

Despite the high prevalence of *Salmonella spp.* among participants with bacteremia, previous reports have found the overall sensitivity of blood cultures to be only 66% (95% CI 56–75%) when compared to more sensitive tests such as bone marrow cultures [61]. Though bone marrow cultures were not performed as part of our study, further molecular and serological testing as part of the AFIRE study identified an additional 51 cases in the false positive and no growth groups (Table 4), 2 of which were fatal. Most participants with negative blood cultures and false positive results (41.5%) had already received IV antibiotics prior to blood collection, which may have substantially diminished the yield of blood cultures [49,50]. While blood collection prior to antibiotic administration is ideal, an environment like Indonesia, where preemptive antibiotic use is common, would significantly benefit from supplementing blood culture testing with molecular and serological tests. These tests do have drawbacks, as molecular diagnostics can have poor sensitivity due to the low organism burden in bodily fluids [62], and serological diagnostics require increasing titers in convalescent specimens compared to acute specimens given high background antibody levels in endemic regions [63]. Further research on combining a clinical prediction algorithm with disease-specific blood cultures for patients with febrile illnesses in typhoid-endemic areas could be a potential route to improve patient outcomes in a community-based setting while waiting for the wider adoption of molecular and serological testing. Among cases of *Salmonella spp*. bacteremia, the prevalence of antimicrobial resistance to the antibiotic of choice was only 3.9% (Fig 2), which is similar to previous studies in Indonesia [64–66]. In the 2011–2015 period, rates of resistance against most antimicrobials for *S.* Typhi and *S.* Paratyphi were low, indicating that there is a distinct epidemiological

dynamic of enteric fever in Indonesia compared to the rest of the world [64,67]. This could be due to different strains of *S.* Typhi and *S.* Paratyphi which may possess different genes that contribute to resistance [64,65], though we did not perform genotyping or sequencing as part of our study.

In addition to *Salmonella spp.* bacteremia, we identified cases of bacteremia caused by other WHO GLASS and non-GLASS pathogens. *E. coli* was the second most common cause of BSI, with over half of isolates possessing some form of antimicrobial resistance. Both fatal cases were found to possess third-generation cephalosporin (3GC) and fluoroquinolone resistance. The global incidence of community-acquired BSI due to *E. coli* is relatively high, with an estimated 50-60 cases per 100,000 population [68–70], and the proportion of 3GC resistance has reached levels >60% in some parts of the world [71,72]. We found 3GC-resistance rates of 35.7% in our study, which is consistent with the WHO GLASS report of 36.6% (interquartile range [IQR] 17.5-58.3) [37]. The fluoroquinolone-resistance rates of 22% that we observed were high but consistent with previous reports from Indonesia [73,74].

Bacteremia from *S. aureus* infection was found in 10.9% cases in our study, and the observed mortality rate of 20% was consistent with a previous report [75]. Both participants who died were diabetic and contracted oxacillin-sensitive infections, suggesting that the cause of death may have been due more to the timing of diagnosis and treatment. It is well-known that diabetics are at high risk for infections with *S. aureus* [76], so comorbidities should be strongly considered when prioritizing blood culture testing. Two participants with systemic lupus erythematosus (SLE) developed *S. aureus* BSIs, which has been associated with classic hyper-IgE syndrome [77]. The colonization of *S. aureus* in the body often increases in patients with SLE and may predispose them to BSI, worsening the SLE itself and

leading to a feedback loop with the potential to reinforce autoimmune symptoms [78,79]. The proportion of MRSA in our study (10%) was lower than the WHO GLASS report (24.9% (IQR 11.4-42.7)) [37], though this is understandable given that our study was not a systematic surveillance of *S. aureus* infections across the country. Geographic variation of CAI with MRSA has been observed in the Asia-Pacific region, including Taiwan, the Philippines, Vietnam, and Sri Lanka (30-39%); Korea and Japan (15- 20%); and Thailand, India, and Hong Kong (3-9%) [80,81]. Data from Indonesia remains limited, but a recent study has shown that the carriage rate of MRSA in the nose and throat of patients admitted to surgery and internal medical wards at Dr. Soetomo Hospital in Surabaya was 8.1% among 643 patients [82]. Additionally, a report on 259 *S. aureus* isolates collected from clinical cultures of patients at four tertiary care hospitals in Denpasar, Malang, Padang, and Semarang found that 6.6% and 18.5% were MRSA and PVL-positive methicillin-susceptible *S. aureus*, respectively [83].

Besides *E. coli* and *S. aureus*, we observed the other WHO GLASS pathogens *K. pneumonia, S. pneumonia*, and *Acinetobacter spp.* in our study. *K. pneumonia* was mostly found in patients with UTI and respiratory illnesses. The two fatal cases were most likely associated with the participants' chronic illnesses (stroke and kidney failure), as none of the isolates were 3GC, fluroquinolone, or co-trimoxazole resistant. Both cases of *S. pneumonia* bacteremia were found in pediatric participants, and both were fatal. The participant with a penicillin-sensitive infection had myelodysplasia syndrome, and the participant with a ceftriaxone-resistant infection had clinical meningitis. *S. pneumonia* was also found by molecular methods in 8 participants whose blood cultures were negative, supporting a previous report that successful diagnostic approaches using blood cultures alone are difficult because of reduced sensitivity [84]. *Acinetobacter lwoffii* was identified in two

participants, both having gastro-intestinal symptoms and receiving an initial diagnosis of typhoid fever. Treatment with cefixime resolved the infections. A similar case with fever, abdominal pain, and diarrhea has been reported in a 64 year-old man in Texas, USA [85].

Our study found the most frequent BSI pathogens to be *S.* Typhi and *E. coli*, though multidrug-resistant *E. coli* was the most problematic. The challenges of AMR in Indonesia are similar to those of many other low and middle-income countries in the region and globally [20]. Misuse and overuse of antibiotics in humans, livestock, and aquaculture may be the key drivers of resistance in the country [86]. Despite current policies related to antimicrobial use in Indonesia, frequent and unnecessary prescription of antibiotics by physicians, high rates of self-medication, and over-the-counter access to antibiotics remain common [87]. Since 2016, the Indonesia Ministry of Health has boosted their AMR stewardship program to tackle this growing challenge, directing substantial funding to the national AMR control committee [20]. Further support for AMR prevention and the alignment of national policies with global policies and standards will substantially improve the growing challenge of AMR infections in Indonesia.

Our study has several limitations. First, the blood specimens analyzed as part of this study were collected only from a limited number of extremely ill patients admitted to tertiary hospitals. Blood culture positivity rates, AMR patterns, and clinical outcomes may not be generalizable to the Indonesian population at-large, though better understanding this critically ill population will hopefully lead to a reduction in mortality from BSIs. Second, only aerobic blood cultures were performed, which may have resulted in missed BSIs caused by anaerobic bacterial. The generally low yield of anaerobic bacteria combined with increasing costs and volumes of blood drawn [13,88,89] make anaerobic cultures impractical for many hospitals in Indonesia. In the future, rationally targeting the use of anaerobic

culture bottles based on careful clinical assessment may result in substantial savings and facilitate the broader adoption of the diagnostic in the country [90]. Lastly, AMR susceptibility testing in this study was performed and reported according to general practice in Indonesia, as our study was not initially designed as an AMR study. Consequently, our data has substantial gaps and missing information. A standardized approach and electronic results reporting system in Indonesia would significantly improve the accuracy and utility of AMR susceptibility testing.

Conclusion

We presented aerobic blood culture findings from a multi-centre study of patients with acute febrile illness admitted to eight major hospitals across Indonesia. Our universal use of aerobic blood cultures is unique in Indonesia, the results of which help clarify the epidemiology and burden of BSI, rates of contamination among CAI, and common AMR patterns in Indonesia. Bacteremia was observed in 8.9% participants, with the most frequent pathogens being *Salmonella spp.*, *E. coli*, and *S. aureus*. Two *Salmonella spp.* cases had evidence of AMR, and several *E. coli* cas[illegible]esistant (42.9%) or monoresistant (14.3%). Culture contamination was observed in 3.6% cases. Our data suggest that blood cultures should be included as a routine diagnostic test, and pre-screening patients for the most common viral infections, such as dengue, influenza and chikungunya viruses, would conserve scarce resources without negatively impacting patient benefit. The routine practice of AMR susceptibility testing on positive blood cultures in Indonesia is encouraging and should be continued to inform clinical decisions on patient treatment in real-time. The country could benefit from clear guidance at the national level,

mention serovar name whether Typhi or Paratyphi A

particularly regarding the timing of blood collection prior to antibiotic administration, the prioritization of patients with comorbidities, blood collection practices to reduce environmental contamination, and the supplementation of blood cultures with molecular assays to combat false-negative results. Additionally, Indonesia could greatly benefit from a nationwide program for the systematic collection and dissemination of blood culture and AMR results.

Acknowledgements

We would like to thank all of the patients who participated in this study, the site study teams and investigators, US-NIAID and Indonesia NIHRD, the Indonesian Ministry of Health, the INA-RESPOND Network Steering Committee, and the sample repository team.

References

1. David A. Smith, Sara M. Nehring. Bacteremia. Treasure Island (FL): StatPearls Publishing; 2022. Available: https://www.ncbi.nlm.nih.gov/books/NBK441979/

2. Goto M, Al-Hasan MN. Overall burden of bloodstream infection and nosocomial bloodstream infection in North America and Europe. Clin Microbiol Infect. 2013;19: 501–509. doi:10.1111/1469-0691.12195

3. McNamara JF, Righi E, Wright H, Hartel GF, Harris PNA, Paterson DL. Long-term morbidity and mortality following bloodstream infection: A systematic literature review. J Infect. 2018;77: 1–8. doi:10.1016/j.jinf.2018.03.005

4. Peters RPH, Zijlstra EE, Schijffelen MJ, Walsh AL, Joaki G, Kumwenda JJ, et al. A prospective study of bloodstream infections as cause of fever in Malawi: clinical predictors and implications for management. Trop Med Int Health. 2004;9: 928–934. doi:10.1111/j.1365-3156.2004.01288.x

5. Gaibani P, Rossini G, Ambretti S, Gelsomino F, Pierro AM, Varani S, et al. Blood culture systems: rapid detection – how and why? Int J Antimicrob Agents. 2009;34: S13–S15. doi:10.1016/S0924-8579(09)70559-X

6. William A. Blood Culture Systems: From Patient to Result. In: Azevedo L, editor. Sepsis - An Ongoing and Significant Challenge. InTech; 2012. doi:10.5772/50139

7. Lamy B, Dargère S, Arendrup MC, Parienti J-J, Tattevin P. How to Optimize the Use of Blood Cultures for the Diagnosis of Bloodstream Infections? A State-of-the Art. Front Microbiol. 2016;7. doi:10.3389/fmicb.2016.00697

8. Prestinaci F, Pezzotti P, Pantosti A. Antimicrobial resistance: a global multifaceted phenomenon. Pathog Glob Health. 2015;109: 309–318. doi:10.1179/2047773215Y.0000000030

9. Hu B, Tao Y, Shao Z, Zheng Y, Zhang R, Yang X, et al. A Comparison of Blood Pathogen Detection Among Droplet Digital PCR, Metagenomic Next-Generation Sequencing, and Blood Culture in Critically Ill Patients With Suspected Bloodstream Infections. Front Microbiol. 2021;12: 641202. doi:10.3389/fmicb.2021.641202

10. Trung NT, Thau NS, Bang MH, Song LH. PCR-based Sepsis@Quick test is superior in comparison with blood culture for identification of sepsis-causative pathogens. Sci Rep. 2019;9: 13663. doi:10.1038/s41598-019-50150-y

11. Weinstein MP. Blood Culture Contamination: Persisting Problems and Partial Progress. J Clin Microbiol. 2003;41: 2275–2278. doi:10.1128/JCM.41.6.2275-2278.2003

12. Hall KK, Lyman JA. Updated Review of Blood Culture Contamination. Clin Microbiol Rev. 2006;19: 788–802. doi:10.1128/CMR.00062-05

13. Dargère S, Parienti J-J, Roupie E, Gancel P-E, Wiel E, Smaiti N, et al. Unique blood culture for diagnosis of bloodstream infections in emergency departments: a prospective multicentre study. Clin Microbiol Infect. 2014;20: O920–O927. doi:10.1111/1469-0691.12656

14. Bates DW. Predicting Bacteremia in Hospitalized Patients: A Prospectively Validated Model. Ann Intern Med. 1990;113: 495. doi:10.7326/0003-4819-113-7-495

15. Salluzzo R, Reilly K. The Rational Ordering of Blood Cultures in the Emergency Department. Qual Assur Util Rev. 1991;6: 28–31. doi:10.1177/0885713x9100600106

16. Little JR, Trovillion E, Fraser V. High Frequency of Pseudobacteremia at a University Hospital. Infect Control Hosp Epidemiol. 1997;18: 200–202. doi:10.1086/647588

17. Teerawattanasook N, Tauran PM, Teparrukkul P, Wuthiekanun V, Dance DAB, Arif M, et al. Capacity and Utilization of Blood Culture in Two Referral Hospitals in Indonesia and Thailand. Am J Trop Med Hyg. 2017;97: 1257–1261. doi:10.4269/ajtmh.17-0193

18. Doern GV, Carroll KC, Diekema DJ, Garey KW, Rupp ME, Weinstein MP, et al. Practical Guidance for Clinical Microbiology Laboratories: A Comprehensive Update on the Problem of Blood Culture Contamination and a Discussion of Methods for Addressing the Problem. Clin Microbiol Rev. 2019;33. doi:10.1128/CMR.00009-19

19. Gasem MH, Kosasih H, Tjitra E, Alisjahbana B, Karyana M, Lokida D, et al. An observational prospective cohort study of the epidemiology of hospitalized patients with acute febrile illness in Indonesia. PLoS Negl Trop Dis. 2020;14: e0007927. doi:10.1371/journal.pntd.0007927

20. Parathon H, Kuntaman K, Widiastoety TH, Muliawan BT, Karuniawati A, Qibtiyah M, et al. Progress towards antimicrobial resistance containment and control in Indonesia. BMJ. 2017; j3808. doi:10.1136/bmj.j3808

21. Gasem MH, Wagenaar JFP, Goris MGA, Adi MS, Isbandrio BB, Hartskeerl RA, et al. Murine typhus and leptospirosis as causes of acute undifferentiated fever, Indonesia. Emerg Infect Dis. 2009;15: 975–977. doi:10.3201/eid1506.081405

22. Capeding MR, Chua MN, Hadinegoro SR, Hussain IIHM, Nallusamy R, Pitisuttithum P, et al. Dengue and other common causes of acute febrile illness in Asia: an active surveillance study in children. PLoS Negl Trop Dis. 2013;7: e2331. doi:10.1371/journal.pntd.0002331

23. Punjabi NH, Taylor WRJ, Murphy GS, Purwaningsih S, Picarima H, Sisson J, et al. Etiology of acute, non-malaria, febrile illnesses in Jayapura, northeastern Papua, Indonesia. Am J Trop Med Hyg. 2012;86: 46–51. doi:10.4269/ajtmh.2012.10-0497

24. Kosasih H, de Mast Q, Widjaja S, Sudjana P, Antonjaya U, Ma'roef C, et al. Evidence for endemic chikungunya virus infections in Bandung, Indonesia. PLoS Negl Trop Dis. 2013;7: e2483. doi:10.1371/journal.pntd.0002483

25. Utama IMS, Lukman N, Sukmawati DD, Alisjahbana B, Alam A, Murniati D, et al. Dengue viral infection in Indonesia: Epidemiology, diagnostic challenges, and mutations from an observational cohort study. Messer WB, editor. PLoS Negl Trop Dis. 2019;13: e0007785. doi:10.1371/journal.pntd.0007785

26. Kosasih H, Alisjahbana B, Nurhayati null, de Mast Q, Rudiman IF, Widjaja S, et al. The Epidemiology, Virology and Clinical Findings of Dengue Virus Infections in a Cohort of Indonesian Adults in Western Java. PLoS Negl Trop Dis. 2016;10: e0004390. doi:10.1371/journal.pntd.0004390

27. Pranata IWA, Diana A, Heryanto MR, Lukman N, Kosasih H, Djauhari H, et al. Persistence of anti-Salmonella O9 IgM as measured by Tubex® TF may contribute to the over-diagnosis of typhoid fever in endemic areas. Bali Med J. 2022;11: 11. doi:10.15562/bmj.v11i1.3035

28. Zeng Z, Zhan J, Chen L, Chen H, Cheng S. Global, regional, and national dengue burden from 1990 to 2017: A systematic analysis based on the global burden of disease study 2017. EClinicalMedicine. 2021;32: 100712. doi:10.1016/j.eclinm.2020.100712

29. Harapan H, Michie A, Yohan B, Shu P-Y, Mudatsir M, Sasmono RT, et al. Dengue viruses circulating in Indonesia: A systematic review and phylogenetic analysis of data from five decades. Rev Med Virol. 2019;29: e2037. doi:10.1002/rmv.2037

30. Harapan H, Michie A, Mudatsir M, Sasmono RT, Imrie A. Epidemiology of dengue hemorrhagic fever in Indonesia: analysis of five decades data from the National Disease Surveillance. BMC Res Notes. 2019;12: 350. doi:10.1186/s13104-019-4379-9

31. Kosasih H, Roselinda null, Nurhayati null, Klimov A, Xiyan X, Lindstrom S, et al. Surveillance of influenza in Indonesia, 2003–2007. Influenza Other Respir Viruses. 2013;7: 312–320. doi:10.1111/j.1750-2659.2012.00403.x

32. Kosasih H, Widjaja S, Surya E, Hadiwijaya SH, Butarbutar DPR, Jaya UA, et al. Evaluation of two IgM rapid immunochromatographic tests during circulation of Asian lineage Chikungunya virus. Southeast Asian J Trop Med Public Health. 2012;43: 55–61.

33. Centers for Disease Control and Prevention. Influenza (Flu). Guidance: Use of Rapid Diagnostic Test. 2016. Available: https://www.cdc.gov/flu/professionals/diagnosis/clinician_guidance_ridt.htm#:~:text=Sensitivities%20of%20RIDTs%20are%20generally,commonly%20than%20false%20positive%20results.

34. Chaterji S, Allen JC, Chow A, Leo Y-S, Ooi E-E. Evaluation of the NS1 rapid test and the WHO dengue classification schemes for use as bedside diagnosis of acute dengue fever in adults. Am J Trop Med Hyg. 2011;84: 224–228. doi:10.4269/ajtmh.2011.10-0316

35. Gonzalez MD, Chao T, Pettengill MA. Modern Blood Culture: Management Decisions and Method Options. Clin Lab Med. 2020;40: 379–392. doi:10.1016/j.cll.2020.07.001

36. Brisse S, Stefani S, Verhoef J, Van Belkum A, Vandamme P, Goessens W. Comparative evaluation of the BD Phoenix and VITEK 2 automated instruments for identification of isolates of the Burkholderia cepacia complex. J Clin Microbiol. 2002;40: 1743–1748. doi:10.1128/JCM.40.5.1743-1748.2002

37. World Health Organization. Global antimicrobial resistance surveillance system: manual for early implementation. 2015. Available: http://www.who.int/antimicrobial-resistance/publications/surveillance-system-manual/en/

38. Vincent J-L, Marshall JC, Namendys-Silva SA, François B, Martin-Loeches I, Lipman J, et al. Assessment of the worldwide burden of critical illness: the intensive care over nations (ICON) audit. Lancet Respir Med. 2014;2: 380–386. doi:10.1016/S2213-2600(14)70061-X

39. Fleischmann C, Scherag A, Adhikari NKJ, Hartog CS, Tsaganos T, Schlattmann P, et al. Assessment of Global Incidence and Mortality of Hospital-treated Sepsis. Current Estimates and Limitations. Am J Respir Crit Care Med. 2016;193: 259–272. doi:10.1164/rccm.201504-0781OC

40. Trunfio M, Savoldi A, Viganò O, d'Arminio Monforte A. Bacterial coinfections in dengue virus disease: what we know and what is still obscure about an emerging concern. Infection. 2017;45: 1–10. doi:10.1007/s15010-016-0927-6

41. Thein T-L, Ng E-L, Yeang MS, Leo Y-S, Lye DC. Risk factors for concurrent bacteremia in adult patients with dengue. J Microbiol Immunol Infect. 2017;50: 314–320. doi:10.1016/j.jmii.2015.06.008

42. See KC, Phua J, Yip HS, Yeo LL, Lim TK. Identification of concurrent bacterial infection in adult patients with dengue. Am J Trop Med Hyg. 2013;89: 804–810. doi:10.4269/ajtmh.13-0197

43. Sudjana P, Jusuf H. Concurrent dengue hemorrhagic fever and typhoid fever infection in adult: case report. Southeast Asian J Trop Med Public Health. 1998;29: 370–372.

44. Lee A, Mirrett S, Reller LB, Weinstein MP. Detection of Bloodstream Infections in Adults: How Many Blood Cultures Are Needed? J Clin Microbiol. 2007;45: 3546–3548. doi:10.1128/JCM.01555-07

45. Wilson ML, Clinical and Laboratory Standards Institute. Principles and procedures for blood cultures: approved guideline. Wayne, Pa.: Clinical and Laboratory Standards Institute; 2007.

46. Murni IK, Duke T, Daley AJ, Kinney S, Soenarto Y. True Pathogen or Contamination: Validation of Blood Cultures for the Diagnosis of Nosocomial Infections in a Developing Country. J Trop Pediatr. 2018;64: 389–394. doi:10.1093/tropej/fmx081

47. Rhodes J, Jorakate P, Makprasert S, Sangwichian O, Kaewpan A, Akarachotpong T, et al. Population-based bloodstream infection surveillance in rural Thailand, 2007–2014. BMC Public Health. 2019;19: 521. doi:10.1186/s12889-019-6775-4

48. Chang C-J, Wu C-J, Hsu H-C, Wu C-H, Shih F-Y, Wang S-W, et al. Factors Associated with Blood Culture Contamination in the Emergency Department: Critical Illness, End-Stage Renal Disease, and Old Age. Lazzeri C, editor. PLOS ONE. 2015;10: e0137653. doi:10.1371/journal.pone.0137653

49. Scheer CS, Fuchs C, Gründling M, Vollmer M, Bast J, Bohnert JA, et al. Impact of antibiotic administration on blood culture positivity at the beginning of sepsis: a prospective clinical cohort study. Clin Microbiol Infect. 2019;25: 326–331. doi:10.1016/j.cmi.2018.05.016

50. Rand KH, Beal SG, Rivera K, Allen B, Payton T, Lipori GP. Hourly Effect of Pretreatment With IV Antibiotics on Blood Culture Positivity Rate in Emergency Department Patients. Open Forum Infect Dis. 2019;6: ofz179. doi:10.1093/ofid/ofz179

51. Limato R, Nelwan EJ, Mudia M, de Brabander J, Guterres H, Enty E, et al. A multicentre point prevalence survey of patterns and quality of antibiotic prescribing in Indonesian hospitals. JAC-Antimicrob Resist. 2021;3: dlab047. doi:10.1093/jacamr/dlab047

52. She RC, Bender JM. Advances in Rapid Molecular Blood Culture Diagnostics: Healthcare Impact, Laboratory Implications, and Multiplex Technologies. J Appl Lab Med. 2019;3: 617–630. doi:10.1373/jalm.2018.027409

53. Harris AM, Bramley AM, Jain S, Arnold SR, Ampofo K, Self WH, et al. Influence of Antibiotics on the Detection of Bacteria by Culture-Based and Culture-Independent Diagnostic Tests in Patients Hospitalized With Community-Acquired Pneumonia. Open Forum Infect Dis. 2017;4: ofx014. doi:10.1093/ofid/ofx014

54. Jones GR, Lowes JA. The systemic inflammatory response syndrome as a predictor of bacteraemia and outcome from sepsis. QJM. 1996;89: 515–522. doi:10.1093/qjmed/89.7.515

55. Shapiro NI, Wolfe RE, Wright SB, Moore R, Bates DW. Who needs a blood culture? A prospectively derived and validated prediction rule. J Emerg Med. 2008;35: 255–264. doi:10.1016/j.jemermed.2008.04.001

56. Seymour CW, Liu VX, Iwashyna TJ, Brunkhorst FM, Rea TD, Scherag A, et al. Assessment of Clinical Criteria for Sepsis: For the Third International Consensus Definitions for Sepsis and Septic Shock (Sepsis-3). JAMA. 2016;315: 762. doi:10.1001/jama.2016.0288

57. Ombelet S, Barbé B, Affolabi D, Ronat J-B, Lompo P, Lunguya O, et al. Best Practices of Blood Cultures in Low- and Middle-Income Countries. Front Med. 2019;6: 131. doi:10.3389/fmed.2019.00131

58. Seigel TA, Cocchi MN, Salciccioli J, Shapiro NI, Howell M, Tang A, et al. Inadequacy of Temperature and White Blood Cell Count in Predicting Bacteremia in Patients with Suspected Infection. J Emerg Med. 2012;42: 254–259. doi:10.1016/j.jemermed.2010.05.038

59. Ochiai RL. a study of typhoid fever in five Asian countries: disease burden and implications for controls. Bull World Health Organ. 2008;86: 260–268. doi:10.2471/BLT.06.039818

60. Nuruzzaman H, Syahrul F. Risk Analysis of Typhoid Fever Based on Personal Hygiene and Street Food Consumption Habit at Home. J Berk Epidemiol. 2016;4: 74–86.

61. Mogasale V, Ramani E, Mogasale VV, Park J. What proportion of Salmonella Typhi cases are detected by blood culture? A systematic literature review. Ann Clin Microbiol Antimicrob. 2016;15: 32. doi:10.1186/s12941-016-0147-z

62. Andrews JR, Ryan ET. Diagnostics for invasive Salmonella infections: Current challenges and future directions. Vaccine. 2015;33: C8–C15. doi:10.1016/j.vaccine.2015.02.030

63. Keddy K, Sooka A, Letsoalo M, Hoyland G, Chaignat CL, Morrissey A, et al. Sensitivity and specificity of typhoid fever rapid antibody tests for laboratory diagnosis at two sub-Saharan African sites. Bull World Health Organ. 2011;89: 640–647. doi:10.2471/BLT.11.087627

64. Hardjo Lugito NP, Cucunawangsih. Antimicrobial Resistance of *Salmonella enterica* Serovars Typhi and Paratyphi Isolates from a General Hospital in Karawaci, Tangerang, Indonesia: A Five-Year Review. Int J Microbiol. 2017;2017: 1–7. doi:10.1155/2017/6215136

65. Chiou C-S, Lauderdale T-L, Phung DC, Watanabe H, Kuo J-C, Wang P-J, et al. Antimicrobial Resistance in Salmonella enterica Serovar Typhi Isolates from Bangladesh, Indonesia, Taiwan, and Vietnam. Antimicrob Agents Chemother. 2014;58: 6501–6507. doi:10.1128/AAC.03608-14

66. Punjabi NH, Agtini MD, Ochiai RL, Simanjuntak CH, Lesmana M, Subekti D, et al. Enteric fever burden in North Jakarta, Indonesia: a prospective, community-based study. J Infect Dev Ctries. 2013;7: 781–787. doi:10.3855/jidc.2629

67. Crump JA, Sjölund-Karlsson M, Gordon MA, Parry CM. Epidemiology, Clinical Presentation, Laboratory Diagnosis, Antimicrobial Resistance, and Antimicrobial Management of Invasive Salmonella Infections. Clin Microbiol Rev. 2015;28: 901–937. doi:10.1128/CMR.00002-15

68. Abernethy J, Guy R, Sheridan EA, Hopkins S, Kiernan M, Wilcox MH, et al. Epidemiology of Escherichia coli bacteraemia in England: results of an enhanced sentinel surveillance programme. J Hosp Infect. 2017;95: 365–375. doi:10.1016/j.jhin.2016.12.008

69. Bou-Antoun S, Davies J, Guy R, Johnson AP, Sheridan EA, Hope RJ. Descriptive epidemiology of Escherichia coli bacteraemia in England, April 2012 to March 2014. Eurosurveillance. 2016;21. doi:10.2807/1560-7917.ES.2016.21.35.30329

70. Williamson DA, Lim A, Wiles S, Roberts SA, Freeman JT. Population-based incidence and comparative demographics of community-associated and healthcare-associated Escherichia coli bloodstream infection in Auckland, New Zealand, 2005 – 2011. BMC Infect Dis. 2013;13: 385. doi:10.1186/1471-2334-13-385

71. Diekema DJ, Hsueh P-R, Mendes RE, Pfaller MA, Rolston KV, Sader HS, et al. The Microbiology of Bloodstream Infection: 20-Year Trends from the SENTRY Antimicrobial Surveillance Program. Antimicrob Agents Chemother. 2019;63. doi:10.1128/AAC.00355-19

72. Peirano G, Pitout JDD. Extended-Spectrum β-Lactamase-Producing Enterobacteriaceae: Update on Molecular Epidemiology and Treatment Options. Drugs. 2019;79: 1529–1541. doi:10.1007/s40265-019-01180-3

73. Dahesihdewi A, Sugianli AK, Parwati I. The surveillance of antibiotics resistance in Indonesia: a current reports. Bali Med J. 2019;8: 565. doi:10.15562/bmj.v8i2.1386

74. Lestari ES, Severin JA, Filius PMG, Kuntaman K, Duerink DO, Hadi U, et al. Antimicrobial resistance among commensal isolates of Escherichia coli and Staphylococcus aureus in the Indonesian population inside and outside hospitals. Eur J Clin Microbiol Infect Dis. 2007;27: 45–51. doi:10.1007/s10096-007-0396-z

75. van Hal SJ, Jensen SO, Vaska VL, Espedido BA, Paterson DL, Gosbell IB. Predictors of Mortality in Staphylococcus aureus Bacteremia. Clin Microbiol Rev. 2012;25: 362–386. doi:10.1128/CMR.05022-11

76. Hansen M-LU, Gotland N, Mejer N, Petersen A, Larsen AR, Benfield T, et al. Diabetes increases the risk of disease and death due to *Staphylococcus aureus* bacteremia. A matched case-control and cohort study. Infect Dis. 2017;49: 689–697. doi:10.1080/23744235.2017.1331463

77. Schopfer K, Feldges A, Baerlocher K, Parisot RF, Wilhelm JA, Matter L. Systemic lupus erythematosus in Staphylococcus aureus hyperimmunoglobulinaemia E syndrome. BMJ. 1983;287: 524–526. doi:10.1136/bmj.287.6391.524

78. Battaglia M, Garrett-Sinha LA. Bacterial infections in lupus: Roles in promoting immune activation and in pathogenesis of the disease. J Transl Autoimmun. 2021;4: 100078. doi:10.1016/j.jtauto.2020.100078

79. Sirobhushanam S, Parsa N, Reed TJ, Berthier CC, Sarkar MK, Hile GA, et al. Staphylococcus aureus Colonization Is Increased on Lupus Skin Lesions and Is Promoted by IFN-Mediated Barrier Disruption. J Invest Dermatol. 2020;140: 1066-1074.e4. doi:10.1016/j.jid.2019.11.016

80. Song J-H, Hsueh P-R, Chung DR, Ko KS, Kang C-I, Peck KR, et al. Spread of methicillin-resistant Staphylococcus aureus between the community and the hospitals in Asian countries: an ANSORP study. J Antimicrob Chemother. 2011;66: 1061–1069. doi:10.1093/jac/dkr024

81. Chuang Y-Y, Huang Y-C. Molecular epidemiology of community-associated meticillin-resistant Staphylococcus aureus in Asia. Lancet Infect Dis. 2013;13: 698–708. doi:10.1016/S1473-3099(13)70136-1

82. Kuntaman K, Hadi U, Setiawan F, Koendori EB, Rusli M, Santosaningsih D, et al. Prevalence of methicillin resistant *Staphylococcus aureus* from nose and throat of patients on admission to medical wards of Dr Soetomo Hospital, Surabaya, Indonesia. Southeast Asian J Trop Med Public Health. 2016;47: 66–70.

83. Santosaningsih D, Santoso S, Budayanti NS, Suata K, Lestari ES, Wahjono H, et al. Characterisation of clinical *Staphylococcus aureus* isolates harbouring *mecA* or Panton-Valentine leukocidin genes from four tertiary care hospitals in Indonesia. Trop Med Int Health. 2016;21: 610–618. doi:10.1111/tmi.12692

84. Korsgaard J, Møller JK, Kilian M. Antibiotic treatment and the diagnosis of Streptococcus pneumoniae in lower respiratory tract infections in adults. Int J Infect Dis. 2005;9: 274–279. doi:10.1016/j.ijid.2004.07.013

85. Regalado NG, Martin G, Antony SJ. Acinetobacter lwoffii: Bacteremia associated with acute gastroenteritis. Travel Med Infect Dis. 2009;7: 316–317. doi:10.1016/j.tmaid.2009.06.001

86. Van Boeckel TP, Brower C, Gilbert M, Grenfell BT, Levin SA, Robinson TP, et al. Global trends in antimicrobial use in food animals. Proc Natl Acad Sci. 2015;112: 5649–5654. doi:10.1073/pnas.1503141112

87. Hadi U, Duerink DO, Lestari ES, Nagelkerke NJ, Werter S, Keuter M, et al. Survey of antibiotic use of individuals visiting public healthcare facilities in Indonesia. Int J Infect Dis. 2008;12: 622–629. doi:10.1016/j.ijid.2008.01.002

88. Kirn TJ, Weinstein MP. Update on blood cultures: how to obtain, process, report, and interpret. Clin Microbiol Infect. 2013;19: 513–520. doi:10.1111/1469-0691.12180

89. Bartlett JG, Dick J. The controversy regarding routine anaerobic blood cultures. Am J Med. 2000;108: 505–506. doi:10.1016/S0002-9343(00)00321-1

90. Lafaurie M, d'Anglejan E, Donay JL, Glotz D, Sarfati E, Mimoun M, et al. Utility of anaerobic bottles for the diagnosis of bloodstream infections. BMC Infect Dis. 2020;20: 142. doi:10.1186/s12879-020-4854-x

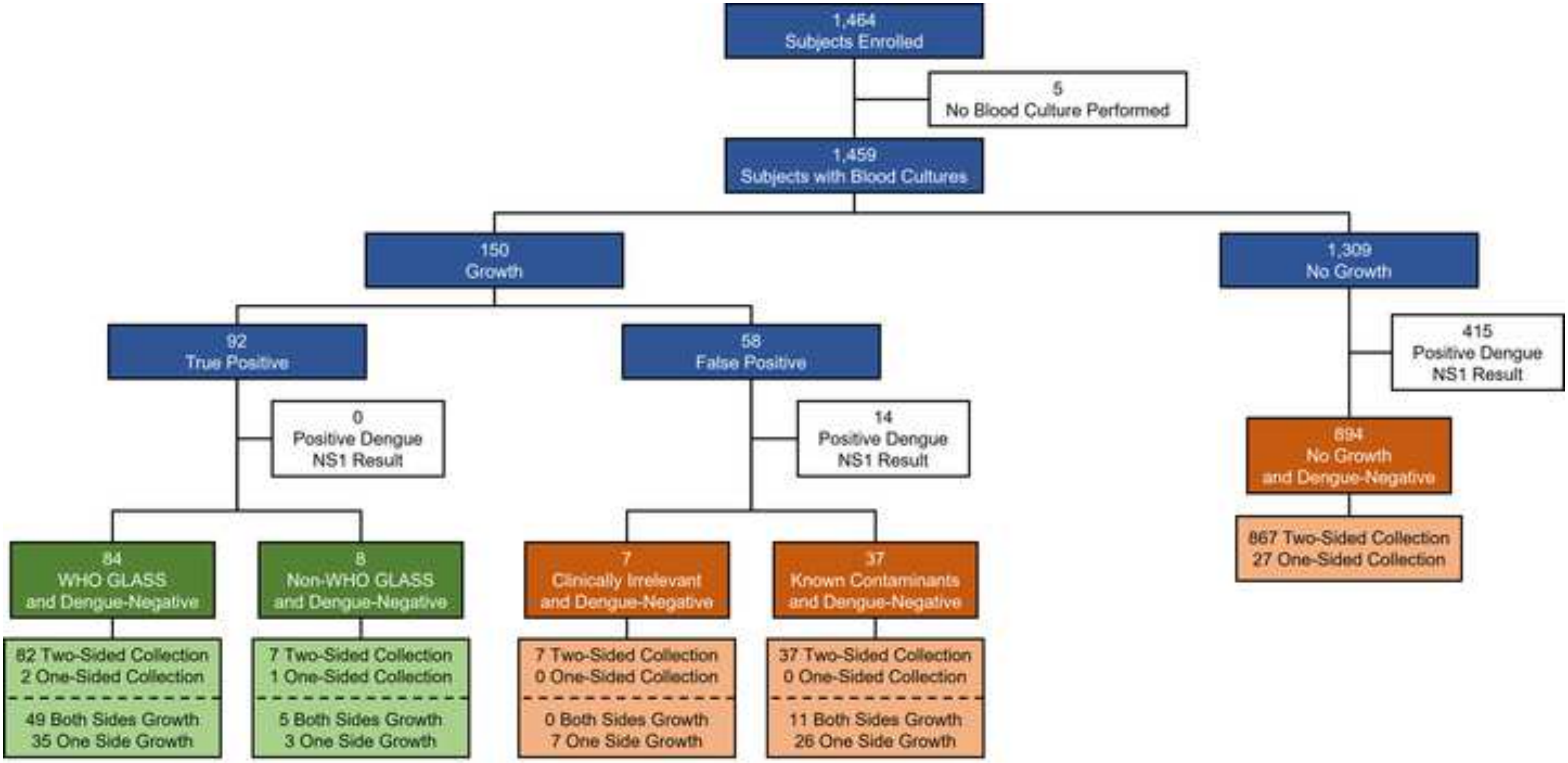
1,464
Subjects Enrolled
5
No Blood Culture Performed
1,459
Subjects with Blood Cultures
150
Growth
1,309
No Growth
92
True Positive
58
False Positive
415
Positive Dengue
NS1 Result
0
Positive Dengue
NS1 Result
14
Positive Dengue
NS1 Result
894
No Growth
and Dengue-Negative
867 Two-Sided Collection
27 One-Sided Collection
84
WHO GLASS
and Dengue-Negative
8
Non-WHO GLASS
and Dengue-Negative
7
Clinically Irrelevant
and Dengue-Negative
37
Known Contaminants
and Dengue-Negative
82 Two-Sided Collection
2 One-Sided Collection
49 Both Sides Growth
35 One Side Growth
7 Two-Sided Collection
1 One-Sided Collection
5 Both Sides Growth
3 One Side Growth
7 Two-Sided Collection
0 One-Sided Collection
0 Both Sides Growth
7 One Side Growth
37 Two-Sided Collection
0 One-Sided Collection
11 Both Sides Growth
26 One Side Growth

Figure 2

Click here to access/download;Figure;Figure 2 - PLOS ONE.tiff

WHO GLASS Pathogens (Cases)	Participant	WHO Recommended Antimicrobial Susceptibility Test												Outcome
Salmonella spp. (51)		**Ciprofloxacin**	**Levofloxacin**	**Ceftriaxone**	**Cefotaxime**	**Ceftazidime**	**Imipenem**	**Meropenem**	**Ertapenem**	**Doripenem**				
	SUB-A	R	R	R	R	S	S	S	S	-				Alive
	SUB-B	-	-	R	S	S	S	S	S	-				Alive
	Others (49)	S (37), ND (12)	S (35), ND (14)	S (42), ND (7)	S (25), ND (24)	S (48), ND (1)	S (9), ND (40)	S (46), ND (3)	S (22), ND (27)	S (1), ND (48)				Alive (46/49)
Escherichia coli (14)		**Ciprofloxacin**	**Levofloxacin**	**Ceftriaxone**	**Cefotaxime**	**Ceftazidime**	**Imipenem**	**Meropenem**	**Ertapenem**	**Doripenem**	**Cotrimoxazole**	**Cefepime**	**Ampicillin**	
	SUB-C	R	R	S	S	S	S	S	S	-	R	-	R	Alive
	BDG-A	R	-	R	-	R	-	S	-	-	R	R	-	Alive
	JOG-A	R	R	R	R	R	-	S	S	-	-	R	R	Dead
	DPS-A	-	I	R	R	R	S	S	-	S	-	-	R	Dead
	JOG-B	S	-	R	-	R	-	-	S	-	-	-	R	Alive
	SUB-D	-	-	R	R	R	S	S	S	-	S	-	R	Alive
	DPS-B	S	-	-	S	S	S	S	S	-	-	S	R	Alive
	SUB-E	S	S	S	S	S	S	S	S	-	S	-	R	Alive
	Others (4)	S (4)	S (4)	S (4)	S (2), ND (2)	S (4)	S (1), ND (3)	S (4)	S (1), ND (3)	ND (4)	S (2), ND (2)	S (3), ND (1)	S (1), ND (3)	Alive (4/4)
	No Data (2)	-	-	-	-	-	-	-	-	-	-	-	-	Alive (2/2)
Staphylococcus aureus (10)		**Oxacillin**												
	SUB-F	R												Alive
	Others (6)	S (6)												Alive (4/6)
	No Data (3)	-												Alive (3/3)
Streptococcus pneumoniae (2)		**Oxacillin**	**Penicillin G**	**Ceftriaxone**	**Cefotaxime**	**Cotrimoxazole**								
	BDG-B	-	-	R	S	R								Dead
	Other (1)	-	-	S	S	-								Dead
Acinetobacter spp. (2)		**Tigecyclin**	**Gentamycin**	**Amikacin**	**Imipenem**	**Meropenem**	**Doripenem**							
	Other (1)	S	S	S	S	S	S							Alive
	No Data (1)	-	-	-	-	-	-							Alive
Klebsiella pneumoniae (5)		**Ciprofloxacin**	**Levofloxacin**	**Ceftriaxone**	**Cefotaxime**	**Ceftazidime**	**Imipenem**	**Meropenem**	**Ertapenem**	**Doripenem**	**Cotrimoxazole**	**Cefepime**		
	Others (5)	S (4), ND (1)	S (4), ND (1)	S (3), ND (2)	S (3), ND (2)	S (5)	-	S (3), ND (2)	S (2), ND (3)	-	S (2), ND (3)	S (4), ND (1)		Alive (3/5)

Click here to access/download
Supporting Information
BLOOD CULTURE DATASET_13MAR2022.xlsx

The Characteristics of Bacteremia among Patients with Acute Febrile Illness Requiring Hospitalization in Indonesia

Pratiwi Soedarmono[1], Aly Diana[2,3], Patricia Tauran[4], Dewi Lokida[5], Abu Tholib Aman[6], Bachti Alisjahbana[7], Dona Arlinda[8], Emiliana Tjitra[8], Herman Kosasih[2*], Ketut Tuti Parwati Merati[9], Mansyur Arif[4], Muhammad Hussein Gasem[10], Nugroho Harry Susanto[2], Nurhayati Lukman[2], Retna ~~Mustika~~ Indah Sugiyono[2], Usman Hadi[11], Vivi Lisdawati[12], Karine G Fouth Tchos[13], Aaron Neal[13], Muhammad Karyana[2,8]

[1] Faculty of Medicine, Universitas Indonesia/ Dr. Cipto Mangunkusumo Hospital, Jakarta, Indonesia

[2] Indonesia Research Partnership on Infectious Disease (INA-RESPOND), Jakarta, Indonesia

[3] Department of Public Health, Faculty of Medicine, Universitas Padjadjaran, Sumedang, Indonesia

[4] Faculty of Medicine, Universitas Hasanuddin/ Dr. Wahidin Sudirohusodo Hospital, Makassar, Indonesia

[5] Tangerang District Hospital, Tangerang, Banten, Indonesia

[6] Faculty of Medicine, Public Heath, and Nursing, Universitas Gadjah Mada/ Dr. Sardjito Hospital, Yogyakarta, Indonesia

[7] Department of Internal Medicine, Faculty of Medicine, Universitas Padjadjaran/ Dr Hasan Sadikin Hospital, Bandung, Indonesia

[8] National Institute of Health Research and Development (NIHRD), Ministry of Health Republic of Indonesia, Jakarta, Indonesia.

[9] Faculty of Medicine, Udayana University, Sanglah General Hospital, Denpasar, Bali, Indonesia

[10] Faculty of Medicine, Diponegoro University/ Dr. Kariadi Hospital, Semarang, Indonesia

[11] Faculty of Medicine, Universitas Airlangga/ Dr. Soetomo Hospital, Surabaya, Indonesia

[12] Sulianti Saroso Infectious Disease Hospital, Jakarta, Indonesia

[13] National Institute of Allergy and Infectious Disease (NIAID), National Institutes of Health, Bethesda, Maryland, United States of America

* Corresponding author

E-mail: hkosasih@ina-respond.net (HK)

Abstract

Blood culturing remains the “gold standard” for bloodstream infection (BSI) diagnosis, but the method is inaccessible to many developing countries due to high costs and insufficient resources. To better understand the utility of blood cultures among patients in Indonesia, a country where blood cultures are not routinely performed, we evaluated data from a previous cohort study that included blood cultures for all participants. An acute febrile illness study was conducted from July 2013 to June 2016 at eight major hospitals in seven provincial capitals in Indonesia. All participants presented with a fever, and two-sided aerobic blood cultures were performed within 48 hours of hospital admission. Positive cultures were further assessed for antimicrobial resistance (AMR) patterns. Specimens from participants with negative culture results were screened by advanced molecular and serological methods for evidence of causal pathogens. Blood cultures were performed for 1,459 of 1,464 participants, and the 70.6% (1,030~~ (70.6%)~~) participants that were negative by dengue NS1 antigen test were included in further analysis. Bacteremia was observed in ~~92 (~~8.9~~%)~~% (92) participants, with the most frequent pathogens being *Salmonella spp.* (51), *Escherichia coli* (14), and *Staphylococcus aureus* (10). Two *Salmonella spp.* cases had evidence of AMR, and several *E. coli* cases were multidrug resistant (42.9%, 6/14~~, 42.9%)~~) or monoresistant (14.3%, 2/14~~, 14.3%).~~). Culture contamination was observed in ~~37 (~~3.6~~%)~~% (37) cases. Advanced laboratory assays identified culturable pathogens in participants having negative cultures, with 23.1% to 90% of cases being missed by blood cultures. Blood cultures are a valuable diagnostic tool for hospitalized patients presenting with fever. In Indonesia, pre-screening patients for the most common viral infections, such as dengue, influenza, and chikungunya viruses, would maximize the benefit to the patient while also

Formatted: Pattern: Clear

conserving resources. Blood cultures should also be supplemented with advanced laboratory tests when available.

Introduction

Bloodstream infections (BSI) [1] are a significant cause of morbidity and mortality in both developing and developed countries [2–4]. The “gold standard” method for BSI diagnosis remains blood culturing [5–7], a straightforward laboratory technique that is inaccessible to many developing countries due to high costs and insufficient resources. Blood cultures provide both definitive microbiological evidence of infection and serve as a crucial tool to monitor the serious global health threat of antimicrobial resistance (AMR) [8]. The threat of AMR further exacerbates the burden felt in countries without routine access to this diagnostic method, including in Indonesia, and allows AMR to continue threatening populations worldwide. The early and accurate identification of causative microorganisms and their susceptibility to antibiotics is essential to improve patient survival and prevent emerging AMR pathogens.

Even with access to routine blood cultures, the interpretation of results can be challenging and should align with clinical observations. Bacterial growth is a consequence of the initial quantity of bacteria in the specimen, the quality of the specimen, the timing of specimen collection with clinical treatment, and the biological nature of the bacteria. Negative blood cultures alone are not definitive for diagnosis, as advanced laboratory methods often detect missed culturable organisms from the same specimen types [9,10]. Routine analysis of specimens can be impacted by contamination from the environment of

the patient [11,12]. In most settings, only 5 to 13% of blood cultures will become positive, and of those, 20–56% result from contamination [7,13–16].

In Indonesia, acute febrile illness resulting from BSIs remains a common cause of hospitalization, morbidity, and mortality. Although infectious diseases are the primary cause of hospitalization in the country, clinicians do not routinely perform blood cultures as part of standard clinical care [17]. When clinicians perform blood cultures, generally in severely ill patients referred to tertiary care, they do not consistently use best laboratory practices [18]. Data on blood culture use, performance, and contamination rates in Indonesia remain very limited [17,19,20]. Consequently, data on the emergence and spread of AMR pathogens in the country is unreliable and incomplete, complicating antibiotic stewardship efforts in the region.

The epidemiology of pathogens associated with fever in Indonesia is not well understood, as public health surveillance data is limited and only a few local studies have been conducted [19,21–26]. Among published studies, dengue virus, chikungunya virus, influenza virus, *Salmonella* Typhi, *Rickettsia spp.*, and *Leptospira spp.* are consistently the most common causes of acute febrile illness hospitalizations. A study in Papua from November 1997 to February 2000 enrolled 226 hospitalized patients that were negative for malaria, the majority of whom were determined to have typhoid fever (18%), leptospirosis (12%), rickettsioses (8%), and dengue fever (7%) [23]. An observational fever study in Bandung identified dengue virus in 12.4% of fever episodes, followed by *S.* Typhi (7.4%), and chikungunya virus (7.1%) [24,26,27]. A 2005-2006 study in Semarang found rickettsioses and leptospirosis in 7% and 10%, respectively, of 137 acute undifferentiated fever cases [21]. The parent study of the research presented here found the most prevalent pathogens among participants at eight hospitals in 7 major cities in Indonesia to be dengue virus (27-

52%), *Rickettsia spp.* (2-12%), *S.* Typhi (0.9-13%), influenza virus (2-6%), *Leptospira spp.* (0-5%), and chikungunya virus (0-4%) [19].

To better understand the utility of blood cultures among patients with acute febrile illness in Indonesia, we evaluated data from a previously published multicenter observational prospective cohort study conducted across the country [19]. Gaining insight into pathogens commonly identified by blood culture, contamination rates, AMR patterns, and disease outcomes will provide actionable evidence to support decision making for Indonesia's national blood culture testing policy.

Methods

Study design and sample collection

A prospective observational study enrolling febrile patients ~~(temperature ≥38°C), aged ≥1 year~~ who required hospitalization was conducted by the Indonesia Research Partnership on Infectious Disease (INA-RESPOND) from July 2013 to June 2016 at eight major hospitals in seven provincial capitals in Indonesia~~.~~: Dr. Cipto Mangunkusumo Hospital in Jakarta, Sulianti Saroso Infectious Disease Hospital in Jakarta, Dr. Wahidin Sudirohusodo Hospital in Makassar, Dr. Sardjito Hospital in Yogyakarta, Dr. Hasan Sadikin Hospital in Bandung, Sanglah General Hospital in Denpasar, Dr. Soetomo Hospital in Surabaya, and Dr. Kariadi Hospital, in Semarang. The full details of this study, known as AFIRE, were published previously ~~[19]~~[19]. Briefly, ~~patients with an unexplained fever for <14 days who were hospitalized~~inclusion criteria consisted of axillary body temperature ≥38˚C, ≥1 year of age, and hospitalization within the past 24 hours ~~and~~. Patients were excluded from the study if they had ~~no history of hospitalization~~subjective fever for ≥14 days or were hospitalized in

Formatted: English (United Kingdom)

Formatted: English (United Kingdom)

Formatted: English (United Kingdom)

the preceding threelast 3 months were enrolled. Demographic, clinical, and laboratory data, including hematology results, were collected at baseline, once during days 14–28, and three months after enrollment. Blood and other biological specimens were collected at each study visit.

During the baseline visit, blood was collected for cultures and for, clinically relevant rapid diagnostic test to detect dengue virus and *Salmonella* Typhi infection based on clinical judgement. All other specimens were stored and tested retrospectively fortests when available, and dengue virus rapid diagnostic tests. Dengue virus infection remains a significant burden across Indonesia [28,29], with disease incidence increasing in recent years [30]. Though other viral agents are present in Indonesia, none are as prevalent as dengue virus [24,31], and most are challenging to diagnose due to limitations with available rapid diagnostic tests [32,33]. Given the widespread prevalence of dengue virus infection, and the very high specificity (almost 100%) and good sensitivity (70-87%) of NS1 antigen rapid diagnostic tests [34], we employed universal dengue virus screening to rapidly resolve the unknown etiologies of study participants. Participants with negative NS1 antigen tests were further pathogen identificationconsidered for BSIs through blood culture tests and other etiologies, as determined through advanced testing at the INA-RESPOND reference laboratory.

Laboratory tests

Blood culture tests for aerob bacterialAerobic blood cultures were performed within 48 hours of a participant being admitted to the emergency department of a study site. Blood volumes of approximately 5-8 mL for adults and 1-3 mL for pediatrics were collected

Formatted: English (United Kingdom)

Formatted: English (United Kingdom)

Formatted: English (Indonesia)

Formatted: English (Indonesia)

from each arm, whenever possible, directly into separate aerobic blood culture bottles. If blood could not be collected from each arm due to clinical reasons, blood was collected from a single arm for a single aerobic blood culture ~~bottles~~bottle. Study physicians were advised to delay the administration of IV antibiotics until blood specimens were collected, provided that there were no immediate risks to the participant. Each hospital performed a complete blood count (CBC) as part of standard-of-care procedures during enrollment.

~~Inoculated aerobic blood culture bottles were incubated using a continuous-monitoring blood culture system, either BACTEC (Becton-Dickinson, Sparks, Maryland) or BacT/Alert (bioMérieux, Inc., Durham, North Carolina) [21]. Manufacturer guidelines were followed for all bacterial cultures, and automated growth identification systems, either BD Phoenix (Becton Dickinson) or VITEK 2 (bioMérieux, Inc., Durham, North Carolina), were used for bacterial identification and antibiotic susceptibility testing. Organism identification is acceptable when the confidence level in automated growth identification system is ≥95% probability [22].~~

Inoculated aerobic blood culture bottles were incubated using a continuous-monitoring blood culture system, either BACTEC (Becton-Dickinson, Sparks, Maryland) or BacT/Alert (bioMérieux, Inc., Durham, North Carolina) [35]. Manufacturer guidelines were followed for all bacterial cultures, and automated growth identification systems, either BD Phoenix (Becton Dickinson) or VITEK 2 (bioMérieux, Inc., Durham, North Carolina), were used for bacterial identification and antibiotic susceptibility testing. Blood cultures were performed and analyzed at the hospitals' nationally accredited clinical laboratories by trained, certified staff. All instruments and standards were calibrated appropriately according to manufacturer guidelines, and all tests were run alongside appropriate positive and negative control to ensure the integrity and accuracy of the results. Organism

identification was considered acceptable when the confidence level in the automated growth identification system was ≥95% probability [36]. Quality control tests were performed weekly at all site laboratories, and each new lot of ID cards was tested using validated stocks of culture organisms.

Growth observed in blood cultures was classified as either “true positive” or “false positive.” True positives included pathogenic bacterial species, particularly those identified as priority pathogens by the World Health Organization Global Antimicrobial Resistance and Use Surveillance System (WHO GLASS) ~~[23], observed in at least one blood culture.~~[37], observed in at least one blood culture. Additionally, non-WHO GLASS pathogens found in either one or both cultures and being consistent with clinical manifestations were also considered to be true positives. False positives included growth of bacteria and fungi which were not clinically relevant and growth of known culture contaminants. Bacterial culture contamination was defined as any culture growing viridans group streptococci, *Corynebacterium spp.*, *Bacillus spp.*, *Diphtheroid spp.*, *Micrococcus spp.*, *Propionibacterium spp.*, and coagulase-negative staphylococci ~~[12]~~[12].

At the INA-RESPOND reference laboratory, specimens from all participants were screened for dengue using NS1 antigen ELISA, dengue RT-PCR, and dengue IgM and IgG. Molecular tests in acute specimens and serological tests in acute and convalescent specimens were performed to detect bacterial infections such as *S.* Typhi, *S*. Paratyphi, *Leptospira spp.*, and *Rickettsia typhi*, and viruses such as influenza, chikungunya, and measles. Details of diagnostic assays for this study were previously described [19].

Field Code Changed

Statistical analysis

Data were collected in ~~OpenClinca~~OpenClinica (OpenClinica LLC, MA, USA) and analyzed using STATA v.15.1 (StataCorp LLC, TX, USA). Proportions were compared between categorical variables using Pearson's chi-squared test. The student's t-test was used to assess continuous variables. All p-values were two-sided with a significance level set to p<0.05.

Ethical clearance

Ethical approvals for the AFIRE study were granted by the Institutional Review Boards of the National Institute of Health Research and Development (NIHRD), Indonesia Ministry of Health (KE.01.05/EC/407/2012, dated 23 May 2012), the Faculty of Medicine at the University of Indonesia and RSUPN Dr. Cipto Mangunkusumo Hospital (451/PT02.FK/ETIK/2012, dated 23 July 2012), and RSUD Dr. Soetomo Hospital (192/Panke.KKE/VIII/2012, dated 13 August 2012). All eligible patients who agreed to participate in the study provided written informed consent before enrollment.

Results

A total of 1,464 participants were enrolled in the AFIRE study, and aerobic blood cultures ~~for aerob bacterial~~ were performed for 1,459 participants (Fig 1). The remaining 5 participants had insufficient blood specimens for following reasons: 1 adult was in a severe condition (decreased of consciousness), 2 participants (1 child and 1 adult) self-discharged against medical advice, and the guardians of 2 children refused to allow more blood to be drawn. Bacterial growth was observed for ~~150 (~~10.3~~%)~~% (150) participants, including ~~84~~ ~~(~~56.0~~%)~~% (84) with WHO GLASS pathogens, ~~8 (~~5.3~~%)~~% (8) with other non-WHO GLASS

pathogens, and ~~58 (~~38.7~~%)~~% (58) with false positives. No growth was observed for 89.7% (1,309~~ (89.7%)~~) participants. All participants were screened for dengue virus by NS1 antigen and dengue IgM/IgG antibody tests, resulting in ~~429 (~~29.4~~%)~~% (429) positive results, 415 from "No Growth" and 14 from the "False Positive" group. The remaining 70.6% (1,030 ~~(70.6%)~~) dengue-negative participants were included in this analysis.

Formatted: Pattern: Clear

Formatted: English (Indonesia), Pattern: Clear

Fig 1. General blood culture results observed among study participants. Participants provided blood from either one or both arms for aerobic blood cultures, and bacterial growth was observed from either one or both sides. All participants providing blood underwent screening for dengue virus infection by NS1 antigen test.

Results of blood cultures: community-acquired infection (CAI)

Bacteremia was observed in ~~92 (~~8.9~~%)~~% (92) of the 1,030 dengue-negative participants, with the most frequent pathogens being *Salmonella spp.* in 51 participants, *Escherichia coli* in 14 participants, and *Staphylococcus aureus* in 10 participants (Table 1). Dengue-negative false positive results were observed in ~~44 (~~4.3~~%)~~% (44) participants, with the most frequent microorganism being contaminating coagulase-negative *Staphylococcus spp.* in 32 participants. From the 136 dengue-negative participants with any microbial growth, ~~133 (~~97.8~~%)~~% (133) had blood collected from two sides of the body (Fig 1). Growth from both sides was observed in 58.7% of participants with true positive results and 25.0% of participants with false positive results.

Table 1. Specific blood culture results among dengue-negative study participants.

	Pathogen	Positive Results	Percent of Positive Results Within Group
WHO GLASS Priority Pathogens (N = 84)	*Salmonella spp.*	51	60.7
	Escherichia coli	14	16.7
	Staphylococcus aureus	10	11.9
	Klebsiella pneumoniae	5	6.0
	Acinetobacter spp.	2	2.4
	Streptococcus pneumoniae	2	2.4
Non-WHO GLASS Pathogens (N = 8)	*Pseudomonas aeruginosa*	2	25.0
	Staphylococcus hominis ssp. hominis	1	12.5
	Enterobacter aerogenes	1	12.5
	Enterococcus faecalis	1	12.5
	Pseudomonas cepacea	1	12.5
	Pseudomonas spp.	1	12.5
	Streptococcus pyogenes	1	12.5
Clinically Irrelevant Growth (N = 7)	*Pantoea spp.*	2	28.6
	Sphingomonas paucimobilis	2	28.6
	Alcaligenes faecalis	1	14.3
	Candida pelliculosa	1	14.3
	Rhizobium radiobacter	1	14.3
Contaminants (N = 37)	*Coagulase-Negative Staphylococcus*	32	86.5
	Bacillus spp.	2	5.4
	Micrococcus luteus	1	2.7

	Kocuria spp.	1	2.7
	Streptococcus viridans	1	2.7
No Growth (N = 894)	*None*	0	0.0

Since *Salmonella spp.* were found in over half (55.4%) of true positives (Table 1), participants with true positive results were analyzed in either *Salmonella spp.* or non-*Salmonella spp.* groups (Table 2). Participant demographics revealed nearly equal numbers of male and female participants in the study, with equal numbers of true positive cases in the two groups. Participants in the *Salmonella spp.* group were significantly younger, with a median age of 14 years old, compared to non-*Salmonella spp.* and false positive groups, with median ages of 44 years old and 24.6 years old, respectively. Over 62.7% of *Salmonella spp.* cases were in participants ≤18 years old, while only 26.8% of non-*Salmonella spp.* cases were in this same age range. There were no significant differences between all groups in the days of onset before hospitalization or the length of hospitalization.

Table 2. Participant characteristics, hematology results, and mortality.

	**True Positive (92)**		**False Positive and No Growth (938)**	**Total (1,030)**
	***Salmonella spp.* (51)**	**Non-*Salmonella spp.* (41)**		
Male, N (%)	29 (56.9)	17 (41.5)	502 (53.9)	553 (53.7)
Median age, years (range, IQR)	14 (2.5-54, 14.7)	44 (1-84, 40.0)	24.6 (1-92, 36.5)	24 (1-92, 36.2)
Mean age, years (SD)	16.2 (11.1)[D,E]	39.6 (24.0)[D,F]	28.6 (21.4)[E,F]	28.5 (21.4)
Distribution of cases by age group, N (%)				
1-5 years	4 (7.8)	5 (12.2)	154 (16.4)	163 (15.8)

>5-18 years	28 (54.9)[D,E]	6 (14.6)[D]	184 (19.6)[E]	218 (21.2)
>18-45 years	18 (35.3)	11 (26.8)	365 (38.9)	394 (38.3)
>45-65 years	1 (2.0)[D,E]	13 (31.7)[C,D]	179 (19.1)[C,E]	193 (18.7)
>65 years	0 (0.0)[B,D]	6 (14.6)[C,D]	56 (6.0)[B,C]	62 (6.0)
Days of onset before hospitalization, median (range, IQR)	7 (1-13, 4)	4 (1-15, 4)	4 (1-15, 4)	4 (1-15, 4)
Length of hospitalization, median (range, IQR)	7 (2-38, 4)	8 (2-40, 7)	6 (1-55, 3.3)	6 (1-55, 4)
Received intravenous antibiotics prior to blood collection, N (%)	9 (17.6)[A,E]	16 (39.0)[A]	389 (41.5)[E]	414 (40.2)
Received any antibiotics following blood collection, N (%)	31/31 (100)[E]	18/18 (100)[A,C]	199/269 (74.0)[A,C,E]	248/318 (77.9)
Hematology at enrollment, N (%)				
Leukopenia	13/51 (25.5)[E]	5/41 (12.2)	120/937 (12.8)[E]	138/1029 (13.4)
Normal Leukocyte	35/51 (68.6)[A,E]	19/41 (46.3)[A]	462/937 (49.3)[E]	516/1029 (50.1)
Leukocytosis	3/51 (5.9)[D,E]	17/41 (41.5)[D]	355/937 (37.9)[E]	375/1029 (36.4)
Lymphopenia	16/44 (36.4)[B,D]	26/38 (68.4)[D]	442/810 (54.6)[B]	484/892 (54.3)
Normal Lymphocyte	17/44 (38.6)[A]	7/38 (18.4)[A,C]	285/810 (35.2)[C]	309/892 (34.6)
Lymphocytosis	11/44 (25.0)[E]	5/38 (13.2)	83/810 (10.2)[E]	99/892 (11.1)
Outcome, N (%)				
Died	3 (5.9)[D]	11 (26.8)[D,F]	69 (7.4)[F]	83 (8.1)

Study participants with true positive culture results were sub-categorized into *Salmonella spp.* and non-*Salmonella spp.* groups to better resolve analyses. Comparisons for significance occur across column groups only.

A,B,C indicates p-value <0.05

D,E,F indicates p-value <0.01

Formatted: English (United States)

Formatted: English (United States)

Intravenous antibiotics were administered prior to blood collection significantly less frequently in the *Salmonella spp*. group (17.6%, 9/51~~, 17.6%)~~) compared to other groups (Table 2). All participants with true positive results were administered antibiotics following blood collection, and 74% of participants with false positive results received antibiotics. Hematology profiles at enrollment differed significantly between the *Salmonella spp.* and non-*Salmonella spp.* groups. Leukopenia and normal leukocyte counts were observed in ~~48 (~~94.1~~%)~~% (48) of *Salmonella spp.* cases compared to ~~24 (~~58.5~~%)~~% (24) of non-*Salmonella spp.* cases and ~~582 (~~62.0~~%)~~% (582) of false positive and no growth cases. Similarly, leukocytosis was significantly lower in the *Salmonella spp.* group compared to the other groups. Lymphopenia was observed in ~~16 (~~36.4~~%)~~% (16) of the *Salmonella spp.* cases, which is significantly lower than the ~~26 (~~68.4~~%)~~% (26) non-*Salmonella spp.* cases and the ~~442 (~~54.6~~%)~~% (442) false positive and no growth cases. Mortality was significantly higher in the non-*Salmonella spp.* group compared to the other groups.

Cases of true positives were distributed across age groups and study sites (Table 3). While *Salmonella spp.* were most frequently found in pediatrics (62.7% of cases), *E. coli*, *S. aureus*, and *K. pneumoniae* were most frequently found in adults (85.7%, 80.0%, and 80.0% of cases, respectively). Most *Salmonella spp.* cases were seen in Bandung (BDG, 41.2%), Semarang (SMG, 23.5%), and Surabaya (SUB, 21.6%). This differed significantly from cases seen in Makassar (MKS, 9.8%), Yogyakarta (YOG, 2.0%), Denpasar (DPS, 2.0%), and Jakarta (JKT, 0.0%). Other than *Salmonella spp.*, there were no significant differences in the distribution of pathogens across study sites, likely due to the low numbers of cases.

Table 3. Positive blood culture pathogens by participant age group and study location.

Pathogen Identified	Age group (years old)					Location							Total
	≥1-5	>5-18	>18-45	>45-65	>65	Bdg	Sby	Smr	Dps	Mks	Yog	Jkt	
Salmonella spp.	4	28 (1†)	18 (1†)	1 (1†)	0	21	11	12	1	5	1	0	51
Escherichia coli	1	1	3 (1†)	5 (1†)	4	3	3	0	4	0	3	1	14
Staphylococcus aureus	0	2	4 (1†)	4 (1†)	0	1	1	3	2	1	0	2	10
Klebsiella pneumoniae	0	1	0	3 (2†)	1	0	1	0	2	2	0	0	5
Acinetobacter spp.	0	1	1	0	0	0	0	0	1	0	0	1	2
Enterobacter aerogenes	0	0	1 (1†)	0	0	0	0	0	1	0	0	0	1
Enterococcus faecalis	1	0	0	0	0	0	0	0	0	0	0	1	1
Pseudomonas aeruginosa	1	0	1 (1†)	0	0	0	0	0	0	0	2	0	2
Pseudomonas cepacea	0	0	0	0	1	1	0	0	0	0	0	0	1
Pseudomonas species	0	0	1	0	0	0	1	0	0	0	0	0	1
Streptococcus pneumoniae	1 (1†)	1 (1†)	0	0	0	1	0	0	0	1	0	0	2
Streptococcus pyogenes	0	0	0	1	0	0	0	0	1	0	0	0	1
Staphylococcus hominis ssp hominis	1 (1†)	0	0	0	0	0	0	0	0	0	1	0	1
**Total**	9 (2†)	34 (2†)	29 (5†)	14 (5†)	6	27	17	15	12	9	7	5	92

† Indicates study participants who died

Bdg: Bandung; Sby: Surabaya; Smr: Semarang; Dps: Denpasar; Mks: Makassar; Yog: Yogyakarta; Jkt: Jakarta

The 938 participants in the false positive and no growth groups had specimens screened by other laboratory methods to determine potential etiologies (Table 4). PCR on blood specimens identified etiologies in 168 participants, serology identified etiologies in

220 participants, and other methods identified etiologies in 94 participants. Among the culturable bacterial pathogens identified in these groups were the WHO GLASS pathogens *S.* Typhi (51), *S. pneumoniae* (18), *K. pneumoniae* (8), *A. baumanii* (7), *E. coli* (7), and *S. aureus* (3). When combined with the culture results from the WHO GLASS priority pathogens group in Table 1, 50% of *S.* Typhi cases, 33.3% of *E. coli* cases, 23.1% of *S. aureus* cases, 61.5% of *K. pneumoniae* cases, 77.8% of *Acinetobacter spp.* cases, and 90% of *S.* ~~*pneumoniae* cases in the AFIRE study [19]~~*pneumoniae* cases in the AFIRE study [19] were not identified by blood cultures.

Table 4. Pathogens detected by molecular, serological, or other laboratory methods from participants with false positive and no growth blood cultures.

False Positive and No Growth (N=938)		Confirmatory Methods		
**Pathogen**	**N**	**Blood PCR**	**Serology**	**Other Methods**
Rickettsia typhi	101	65	36	
Influenza	66	0	59	7: Sputum PCR
Salmonella Typhi	51	3	48	
Leptospira spp.	44	31	13	
Chikungunya	38	30	8	
Dengue	35	0	35	
Mycobacterium tuberculosis	20	0	0	20: Sputum Microscopy
Streptococcus pneumoniae	18	10	0	8: Sputum PCR
Measles	14	9	5	
Amoeba	11	0	0	11: Feces Microscopy
RSV	11	0	9	2: Swab PCR
HHV-6	9	9	0	
Klebsiella pneumoniae	8	1	0	5: Sputum Culture

				2: Swab Culture
Acinetobacter baumanii	7	1	0	4: Sputum PCR<br>1: Swab PCR<br>1: Urine PCR
Escherichia coli	7	1	0	4: Urine Culture<br>2: Pus Culture
Hepatitis A	6	0	6	
Pseudomonas aeruginosa	6	0	0	4: Sputum Culture<br>2: Urine Culture
Enterococcus faecalis	3	0	0	2: Pus Culture<br>1: Urine Culture
Staphylococcus aureus	3	0	0	3: Pus Culture
Mycobacterium leprae	2	0	0	2: Skin Microscopy
Plasmodium spp.	2	0	0	2: Rapid Antigen Test
Seoul virus	2	2	0	
Adenovirus	1	1	0	
Ascaris lumbricoides	1	0	0	1: Feces Microscopy
Ascaris lumbricoides and *Trichuris Trichiura*	1	0	0	1: Feces Microscopy
Bordetella pertussis and *Streptococcus pneumoniae*	1	0	0	1: Sputum PCR
HCoV-OC43	1	1	0	
Enterobacter aerogenes	1	0	0	1: Sputum Culture
Enterobacter cloacae	1	0	0	1: Sputum Culture and PCR
Enterococcus avium	1	0	0	1: Pus Culture
Enterovirus	1	1	0	
EPEC	1	0	0	1: Feces Culture
HIV	1	1	0	
Metapneumovirus	1	0	0	1: Swab PCR
Moraxella catarrhalis and Influenza B	1	0	0	1: Sputum Culture and PCR
Mycoplasma pneumoniae	1	0	0	1: Sputum PCR
Norovirus II	1	1	0	
Rickettsia felis	1	1	0	
Rubella	1	0	1	
Streptococcus faecalis	1	0	0	1: Urine Culture

Unknown	456	0	0	
**Total**	**938**	**168**	**220**	**94**

Plasma, serum, and clinically relevant specimens were collected from all study participants and tested in a central lab for culturable and non-culturable pathogens based on a standard study algorithm and clinical suspicion.

Antimicrobial resistance patterns

Antimicrobial resistance patterns were observed in several participants with blood cultures positive for WHO GLASS priority pathogens (Fig 2). Among the 51 *Salmonella spp.* cases, evidence of multidrug resistance was observed in one participant and monoresistance in one participant. In contrast, *E. coli* cases were mostly multidrug resistant (42.9%, 6/14~~, 42.9%)~~) or monoresistant (14.3%, 2/14~~, 14.3%),~~), with observed resistances to ampicillin (~~7/8, ~~87.5~~%),~~%, 7/8), co-trimoxazole (~~3/5, ~~60.0~~%),~~%, 3/5), ceftriaxone (45.4%, 5/11~~, 45.4%),~~), ceftazidime (41.6%, 5/12~~, 41.6%),~~), cefotaxime (~~3/8, ~~37.5~~%),~~%, 3/8), cefepime (~~2/6, ~~33.3~~%),~~%, 2/6), ciprofloxacin (30.0%, 3/10~~, 30.0%),~~), and levofloxacin (~~2/8, ~~25.0~~%).~~%, 2/8). Two participants (JOG-A and DPS-A) receiving ceftriaxone died before their antimicrobial resistance test results, and one participant (JOG-B) survived when switched from ceftazidime to ciprofloxacin based on their test results.

Figure 2. Antimicrobial resistance patterns observed in WHO GLASS priority pathogens from true positive blood cultures. Participants with resistant (R) infections are identified by study location, and participants with sensitive (S) infections or infections with no testing data (ND) are grouped into Other or No Data categories.

Methicillin-resistant *S. aureus* (MRSA) was observed in one participant based on oxacillin susceptibility testing, and two participants with oxacillin-sensitive *S. aureus*

infections died. Both participants with *S. pneumoniae* bacteremia died, though antimicrobial resistance was only observed in one of the participants. All cases of *Acinetobacter spp.* and *K. pneumoniae* that underwent drug sensitivity testing were sensitive to antibiotics.

Disease outcomes

Characteristics and laboratory findings of participants who died during hospitalization are shown in Table 5. A total of 83 participants in this analysis died during hospitalization. Among these, ~~14 (~~16.9~~%)~~% (14) had true positive blood cultures (Table 5A), resulting in 15.2% mortality in the true positive group. This mortality rate is twofold higher than the 7.4% mortality observed in the false positive and no growth groups. Overall mortality in the *Salmonella spp.* group (5.9%) was significantly lower than the non-*Salmonella spp.* group (26.8%). Among deceased participants, there were no significant differences in demographics between the true positive group and false positive and no growth groups. Most deceased participants had comorbidities including diabetes mellitus (4), hepatitis B (3), HIV (2), tuberculosis (2), brain tumor (1), TRALI (1), neoplasia (1), and others (6) (Table 5B). Antimicrobial-resistant pathogens were identified in 3 of the 14 deceased participants with true positives (Table 5). In the false positive and no growth groups, other laboratory methods such as PCR and/or serology were used to identify culturable bacterial pathogens including *S.* Typhi (2), *A. baumanii* (1), *E. avium* (1), *E. coli* (1), *M. catarrhalis* (1), and *S. pneumoniae* (1) (Table 5B).

Table 5. Participant characteristics, clinical diagnoses, and identified pathogens from fatal cases in the study.

(A) Characteristics of deceased participants categorized by blood culture growth result.

	**True Positive (14)**		**False Positive and No Growth (69)**	**Total (83)**
	***Salmonella spp.* (3)**	**Non-*Salmonella spp.* (11)**		
Male, N (%)	3 (100)	7 (63.6)	36 (52.2)	46 (55.4)
Distribution of cases by age group, N (%)				
1-5 years	0 (0.0)	2 (18.2)	4 (5.8)	6 (7.2)
>5-18 years	1 (33.3)	1 (9.1)	7 (10.1)	9 (10.8)
>18-45 years	1 (33.3)	4 (36.4)	24 (34.8)	29 (34.9)
>45-65 years	1 (33.3)	4 (36.4)	25 (36.2)	30 (36.1)
>65 years	0 (0.0)	0 (0.0)	9 (13)	9 (10.8)
Received intravenous antibiotics prior to blood collection, N (%)	1 (33.3)	1 (9.1)	34 (49.3)	36 (43.4)
Length of hospitalization, median (range, IQR)	4 (2-38)	12 (2-17)	8 (2-54)	8 (2-54)
Comorbidities, N (%)	2 (66.6)	10 (90.9)	60 (86.9)	72 (86.7)

(B) Pathogens from fatal cases confirmed by blood culture or other lab methods and the accompanying clinical diagnoses, participant comorbidities, and AMR observations.

**True Positive (14)**	**Clinical Diagnosis at Death**	**Comorbidities**	**Antimicrobial Resistance**
Salmonella spp. (3)	Typhoid fever	Hepatitis B, HIV, TB	None
	Acute limb ischemia	Acute Limb Ischemia	None
	Sepsis, typhoid fever	Transfusion-Related Acute Lung Injury (TRALI)	None
Escherichia coli (2)	Cholangitis	Diabetes, Hepatitis B	Yes
	Sepsis	Anemia	Yes

Klebsiella pneumoniae (2)	UTI, diabetic ketoacidosis	Diabetes	None
	UTI	Stroke	None
Staphylococcus aureus (2)	UTI	Diabetes	None
	Sepsis	Diabetes, Chronic Kidney Disease	None
Streptococcus pneumoniae (2)	Aseptic meningitis, acute otitis media	Epilepsy	Yes
		Myelodysplasia, Hepatitis B (Cirrhosis)	None
Pseudomonas aeruginosa (1)	Stevens-Johnson syndrome	HIV, TB, Toxoplasmosis	No data
Enterobacter aerogenes (1)	Cholangitis, Sepsis	None	No data
Staphylococcus hominis ssp hominis (1)		Craniopharyngioma	None

**False Positive and No Growth (69) [Confirmatory Methods]**	**Clinical Diagnosis at Death**
Mycobacterium tuberculosis (8) [GeneXpert (2), Microscopy (6)]	Pulmonary TB (3), Colitis TB and Spondylitis TB, Millar TB, HIV, Community-acquired Pneumonia, Sepsis
Rickettsia typhi (6) [PCR (6)]	Sepsis (3), Community-acquired Pneumonia, Meningoencephalitis, Diabetic Neuropathy
Influenza (3) [PCR (2), Serology (1)]	Bronchiectasis, Community-acquired Pneumonia, Sepsis
Salmonella Typhi (2) [Serology (2)]	Hirschsprung's disease, HIV
Acinetobacter baumanii (1) [Sputum PCR]	Community-acquired Pneumonia
Ascaris lumbricoides (1) [Microscopy]	Typhoid Fever
Enterococcus avium (1) [Pus culture]	Diabetic Ulcer
Escherichia coli (1) [Urine culture]	UTI
HIV (1) [PCR]	Sepsis
Leptospira spp. (1) [PCR]	Dengue Hemorrhagic Fever I

Moraxella catarrhalis and Influenza B (1) [Sputum culture and sputum PCR]	Community-acquired Pneumonia
RSV (1) [Serology]	TB Pleuritis
Streptococcus pneumoniae (1) [Sputum PCR]	Community-acquired Pneumonia
Unknown (41) [None]	HIV (6), Sepsis (6), Community-acquired Pneumonia (9), Cellulitis (2), Cholangitis (2), Lung Abscess, Acute Leukemia, Bacterial Meningitis, Bronchitis, Cholecystitis, Chronic Myelocytic Leukemia, COPD, Diarrhea, Extrapulmonary TB, GEA, Hepatitis B, Pancytopenia, SLE, Typhoid Fever, UTI, Unknown

Discussion

BSI causes a high burden of morbidity and mortality worldwide, particularly in low- and middle-income countries (LMICs). Exact figures for BSI incidence and associated mortality in LMICs are challenging to find due to the lack of bacteriological laboratories and routine surveillance systems [38,39]. In Indonesia, very few acute febrile patients undergo aerobic blood culture testing since it is not standard practice in the healthcare system, largely due to resource and capacity restrictions [17]. The AFIRE study presents a unique opportunity to improve our understanding of BSIs in the country since aerobic blood cultures were performed on nearly all participants, regardless of clinical suspicion of bacteremia.

~~Microbial growth was observed in 10.3% of all participants, with bacteremia being ultimately confirmed in 6.3% of all participants (Fig 1). These proportions are similar to previous reports, where positivity rates ranged from 10.0 - 11.4% [17]. The high prevalence of dengue fever in Indonesia often complicates the clinical assessment of acute febrile~~

illness [26], so specimens from all participants in the AFIRE study were retrospectively tested for dengue NS1 antigen to exclude dengue as a cause of illness [19]. Data on co-infections with dengue virus and bacteremia is limited [27], though no participants in our study with confirmed bacteremia, or "True Positives," were found to be co-infected. The 14 participants with positive dengue NS1 antigen results showed false positive blood cultures (5 *Staphylococcus hominis*, 4 *Staphylococcus epidermidis*, 1 *Kocuria rosea*, 1 *Micrococcus aureus*, 1 *Staphylococcus arlettae*, 1 coagulase-negative *Staphylococcus spp.*, and 1 *Staphylococcus waneri*)

Among dengue-negative participants with any microbial growth, 97.8% had blood cultures performed from two sides of collection. One-sided blood culture lacks sufficient sensitivity for BSI detection [28], and two-sided cultures make it easier to distinguish true bacteremia and contamination [28,29]. It has been demonstrated that collecting two or more blood culture sets, each comprising two bottles, over twenty-four hours will detect over 94% of bacteremia episodes, compared to a detection rate of only 73% with the first blood culture [28]. In many developing countries, collecting multiple blood culture sets is generally not feasible, but the minimum practice of a single, one-sided blood culture still has value if clinical care teams understand its limitations. Our data suggest that, in situations where a single, one-sided blood culture is performed, the likelihood of missing a case of bacteremia is 39% (35/89) (8.9% (89/1000) vs 5.4% (54/1000) (Fig 1). Indonesian clinicians should consider this reduced sensitivity when acting on culture results.

The reliability and interpretation of blood culture results is significantly affected by both contamination rates and the use of antibiotics prior to blood collection. General target rates for culture contamination have been set at 3% [29], and in our study we observed an overall contamination rate of 3.6%. These findings are consistent with previous reports,

including a 2010-2013 study at Sardjito Hospital in Yogyakarta that found a contamination rate of 4.1% in children at the pediatric ICU and in pediatric wards [30]. Additional reports from rural Thailand and Taiwan found contamination rates ranging from 4.1-6.1% and 2.6%, respectively [31,32]. The proportion of participants who were given intravenous antibiotics prior to blood collection in our study was high (40.2%), and this may alter the blood culture results considerably [33,34]. In Indonesia, antibiotic therapy is often initiated preemptively and without confirmatory testing in an attempt to maximize positive clinical outcomes [35]. This broad use of antibiotics likely masks the true prevalence of bacteremia and may have negative consequences for patients who subsequently appear to have no infection. Among participants with false positives or no growth, 111 had culturable microbes confirmed by other methods (Table 4), 7 of which died (Table 5). 56.8% of these overall participants received antibiotics prior to blood collection. The expansion of molecular methods would significantly help to tackle this problem, as nucleic acid probe and amplification tests have been shown to significantly improve the speed and accuracy of results in blood stream infections even after antibiotic use (33,34).

White blood cell counts, particularly leukopenia and leukocytosis, have been used to predict blood culture results (35–37). However, the accuracy of systemic inflammatory response syndrome (SIRS) criteria [38], Shapiro criteria [39], and the quick Sequential Organ Failure Assessment (qSOFA) score [40] could not be confirmed in our study. This is primarily due to the significant difference in leukocyte profiles between participants with *Salmonella spp.* versus non-*Salmonella spp.* infections. Our study suggests, as proposed by Ombelet [41] and Seigel [42] that leukocytosis should not be used as a predictor for positive blood cultures in *S. enterica*-endemic areas.

We found that *Salmonella spp.* infection was the most common community-acquired BSI (Table 1) at 55.4% of cases, which aligns with previous studies conducted in limited-resource environments [30,31]. The majority of *Salmonella* bacteremia was in pediatrics, which is consistent with a previous report from a blood culture study in Jakarta where the incidence rate of typhoid fever was higher in the 2-15 year age group, with a mean age of onset of 10.2 years [43]. This commonly observed age association may be due to poor hygiene practices or the consumption of foods, particularly street food, outside of the home [44]. Though over half of bacteremia cases were due to *Salmonella spp.* infection, only 21.4% of bacteremia deaths were due to the pathogen. Among these fatal cases, all had significant comorbidities, suggesting that patients with multiple comorbidities would benefit from prioritization of blood culture diagnostics.

Despite the high prevalence of *Salmonella spp.* among participants with bacteremia, previous reports have found the overall sensitivity of blood cultures to be only 66% (95% CI 56–75%) when compared to more sensitive tests such as bone marrow cultures [45]. Though bone marrow cultures were not performed as part of our study, further molecular and serological testing as part of the AFIRE study identified an additional 51 cases in the false positive and no growth groups (Table 4), 2 of which were fatal. Most participants with negative blood cultures and false positive results (41.5%) had already received IV antibiotics prior to blood collection, which may have substantially diminished the yield of blood cultures [33,34]. While blood collection prior to antibiotic administration is ideal, an environment like Indonesia, where preemptive antibiotic use is common, would significantly benefit from supplementing blood culture testing with molecular and serological tests. These tests do have drawbacks, as molecular diagnostics can have poor sensitivity due to the low organism burden in bodily fluids [46], and serological diagnostics require increasing

titers in convalescent specimens compared to acute specimens given high background antibody levels in endemic regions [47]. Further research on combining a clinical prediction algorithm with disease-specific blood cultures for patients with febrile illnesses in typhoid-endemic areas could be a potential route to improve patient outcomes in a community-based setting while waiting for the wider adoption of molecular and serological testing.

Among cases of *Salmonella spp.* bacteremia, the prevalence of antimicrobial resistance to the antibiotic of choice was only 3.9% (Fig 2), which is similar to previous studies in Indonesia [48–50]. In the 2011–2015 period, rates of resistance against most antimicrobials for *S.* Typhi and *S.* Paratyphi were low, indicating that there is a distinct epidemiological dynamic of enteric fever in Indonesia compared to the rest of the world [48,51]. This could be due to different strains of *S.* Typhi and *S.* Paratyphi which may possess different genes that contribute to resistance [48,50], though we did not perform genotyping or sequencing as part of our study.

In addition to *Salmonella spp.* bacteremia, we identified cases of bacteremia caused by other WHO GLASS and non-GLASS pathogens. *E. coli* was the second most common cause of BSI, with over half of isolates possessing some form of antimicrobial resistance. Both fatal cases were found to possess third-generation cephalosporin (3GC) and fluoroquinolone resistance. The global incidence of community-acquired BSI due to *E. coli* is relatively high, with an estimated 50-60 cases per 100,000 population [52–54], and the proportion of 3GC resistance has reached levels >60% in some parts of the world [55,56]. We found 3GC-resistance rates of 35.7% in our study, which is consistent with the WHO GLASS report of 36.6% (interquartile range [IQR] 17.5-58.3) [23]. The fluoroquinolone-resistance rates of 22% that we observed were high but consistent with previous reports from Indonesia [57,58].

Bacteremia from *S. aureus* infection was found in 10.9% cases in our study, and the observed mortality rate of 20% was consistent with a previous report [59]. Both participants who died were diabetic and contracted oxacillin-sensitive infections, suggesting that the cause of death may have been due more to the timing of diagnosis and treatment. It is well-known that diabetics are at high risk for infections with *S. aureus* [60], so comorbidities should be strongly considered when prioritizing blood culture testing. Two participants with systemic lupus erythematosus (SLE) developed *S. aureus* BSIs, which has been associated with classic hyper-IgE syndrome [61]. The colonization of *S. aureus* in the body often increases in patients with SLE and may predispose them to BSI, worsening the SLE itself and leading to a feedback loop with the potential to reinforce autoimmune symptoms [62,63]. The proportion of MRSA in our study (10%) was lower than the WHO GLASS report (24.9% (IQR 11.4–42.7)) [23], though this is understandable given that our study was not a systematic surveillance of *S. aureus* infections across the country. Geographic variation of CAI with MRSA has been observed in the Asia-Pacific region, including Taiwan, the Philippines, Vietnam, and Sri Lanka (30-39%); Korea and Japan (15- 20%); and Thailand, India, and Hong Kong (3-9%) [64,65]. Data from Indonesia remains limited, but a recent study has shown that the carriage rate of MRSA in the nose and throat of patients admitted to surgery and internal medical wards at Dr. Soetomo Hospital in Surabaya was 8.1% among 643 patients [66]. Additionally, a report on 259 *S. aureus* isolates collected from clinical cultures of patients at four tertiary care hospitals in Denpasar, Malang, Padang, and Semarang found that 6.6% and 18.5% were MRSA and PVL-positive methicillin-susceptible *S. aureus*, respectively [67].

Microbial growth was observed in 10.3% of all participants, with bacteremia being ultimately confirmed in 6.3% of all participants (Fig 1). These proportions are similar to

previous reports, where positivity rates ranged from 10.0 - 11.4% [17]. The high prevalence of dengue fever in Indonesia often complicates the clinical assessment of acute febrile illness [25], so specimens from all participants in the AFIRE study were retrospectively tested for dengue NS1 antigen to exclude dengue as a cause of illness [19]. Data on co-infections with dengue virus and bacteremia is limited. A literature review of published case reports and studies from January 1943 to March 2016 found 3 studies in Singapore and Taiwan reporting concurrent bacteremia in 0.18-7% of dengue fever cases [40–42]. A concurrent dengue virus and *S.* Typhi case was also reported from Bandung, Indonesia [43]. In all of these studies, blood was collected for bacterial culture because patients did not improve clinically a few days to a week after dengue fever was diagnosed. Furthermore, in the majority of cases, dengue virus infection was confirmed by serology only (IgM detected or four-fold IgG increase). These reports support our finding that simultaneous infection with bacteria and dengue virus is rare. In our study, bacterial growth observed in 14 participants with positive dengue NS1 antigen tests were considered false positive blood cultures (5 *Staphylococcus hominis*, 4 *Staphylococcus epidermidis*, 1 *Kocuria rosea*, 1 *Micrococcus aureus*, 1 *Staphylococcus arlettae*, 1 coagulase-negative *Staphylococcus spp.*, and 1 *Staphylococcus waneri*).

Among dengue-negative participants with any microbial growth, 97.8% had blood cultures performed from two sides of collection. One-sided blood culture lacks sufficient sensitivity for BSI detection [44], and two-sided cultures make it easier to distinguish true bacteremia and contamination [44,45]. It has been demonstrated that collecting two or more blood culture sets, each comprising two bottles, over twenty-four hours will detect over 94% of bacteremia episodes, compared to a detection rate of only 73% with the first blood culture [44]. In many developing countries, collecting multiple blood culture sets is

generally not feasible, but the minimum practice of a single, one-sided blood culture still has value if clinical care teams understand its limitations. Our data suggest that, in situations where a single, one-sided blood culture is performed, the likelihood of missing a case of bacteremia is 39% (35/89) (8.9% (89/1000) vs 5.4% (54/1000) (Fig 1). Indonesian clinicians should consider this reduced sensitivity when acting on culture results.

The reliability and interpretation of blood culture results is significantly affected by both contamination rates and the use of antibiotics prior to blood collection. General target rates for culture contamination have been set at 3% [45], and in our study we observed an overall contamination rate of 3.6%. These findings are consistent with previous reports, including a 2010-2013 study at Sardjito Hospital in Yogyakarta that found a contamination rate of 4.1% in children at the pediatric ICU and in pediatric wards [46]. Additional reports from rural Thailand and Taiwan found contamination rates ranging from 4.1-6.1% and 2.6%, respectively [47,48]. The proportion of participants who were given intravenous antibiotics prior to blood collection in our study was high (40.2%), and this may alter the blood culture results considerably [49,50]. In Indonesia, antibiotic therapy is often initiated preemptively and without confirmatory testing in an attempt to maximize positive clinical outcomes [51]. This broad use of antibiotics likely masks the true prevalence of bacteremia and may have negative consequences for patients who subsequently appear to have no infection. Among participants with false positives or no growth, 111 had culturable microbes confirmed by other methods (Table 4), 7 of which died (Table 5). 56.8% of these overall participants received antibiotics prior to blood collection. The expansion of molecular methods would significantly help to tackle this problem, as nucleic acid probe and amplification tests have been shown to significantly improve the speed and accuracy of results in blood stream infections even after antibiotic use [52,53].

White blood cell counts, particularly leukopenia and leukocytosis, have been used to predict blood culture results. However, the accuracy of systemic inflammatory response syndrome (SIRS) criteria [54], Shapiro criteria [55], and the quick Sequential Organ Failure Assessment (qSOFA) score [56] could not be confirmed in our study. This is primarily due to the significant difference in leukocyte profiles between participants with *Salmonella spp.* versus non-*Salmonella spp*. infections. Our study suggests, as proposed by Ombelet [57] and Seigel [58] that leukocytosis should not be used as a predictor for positive blood cultures in *S. enterica*-endemic areas.

We found that *Salmonella spp.* infection was the most common community-acquired BSI (Table 1) at 55.4% of cases, which aligns with previous studies conducted in limited-resource environments [46,47]. The majority of *Salmonella* bacteremia was in pediatrics, which is consistent with a previous report from a blood culture study in Jakarta where the incidence rate of typhoid fever was higher in the 2-15 year age group, with a mean age of onset of 10.2 years [59]. This commonly observed age association may be due to poor hygiene practices or the consumption of foods, particularly street food, outside of the home [60]. Though over half of bacteremia cases were due to *Salmonella spp.* infection, only 21.4% of bacteremia deaths were due to the pathogen. Among these fatal cases, all had significant comorbidities, suggesting that patients with multiple comorbidities would benefit from prioritization of blood culture diagnostics.

Despite the high prevalence of *Salmonella spp.* among participants with bacteremia, previous reports have found the overall sensitivity of blood cultures to be only 66% (95% CI 56–75%) when compared to more sensitive tests such as bone marrow cultures [61]. Though bone marrow cultures were not performed as part of our study, further molecular and serological testing as part of the AFIRE study identified an additional 51 cases in the

false positive and no growth groups (Table 4), 2 of which were fatal. Most participants with negative blood cultures and false positive results (41.5%) had already received IV antibiotics prior to blood collection, which may have substantially diminished the yield of blood cultures [49,50]. While blood collection prior to antibiotic administration is ideal, an environment like Indonesia, where preemptive antibiotic use is common, would significantly benefit from supplementing blood culture testing with molecular and serological tests. These tests do have drawbacks, as molecular diagnostics can have poor sensitivity due to the low organism burden in bodily fluids [62], and serological diagnostics require increasing titers in convalescent specimens compared to acute specimens given high background antibody levels in endemic regions [63]. Further research on combining a clinical prediction algorithm with disease-specific blood cultures for patients with febrile illnesses in typhoid-endemic areas could be a potential route to improve patient outcomes in a community-based setting while waiting for the wider adoption of molecular and serological testing. Among cases of *Salmonella spp*. bacteremia, the prevalence of antimicrobial resistance to the antibiotic of choice was only 3.9% (Fig 2), which is similar to previous studies in Indonesia [64–66]. In the 2011–2015 period, rates of resistance against most antimicrobials for *S.* Typhi and *S.* Paratyphi were low, indicating that there is a distinct epidemiological dynamic of enteric fever in Indonesia compared to the rest of the world [64,67]. This could be due to different strains of *S.* Typhi and *S.* Paratyphi which may possess different genes that contribute to resistance [64,65], though we did not perform genotyping or sequencing as part of our study.

In addition to *Salmonella spp.* bacteremia, we identified cases of bacteremia caused by other WHO GLASS and non-GLASS pathogens. *E. coli* was the second most common cause of BSI, with over half of isolates possessing some form of antimicrobial resistance. Both fatal

cases were found to possess third-generation cephalosporin (3GC) and fluoroquinolone resistance. The global incidence of community-acquired BSI due to *E. coli* is relatively high, with an estimated 50-60 cases per 100,000 population [68–70], and the proportion of 3GC resistance has reached levels >60% in some parts of the world [71,72]. We found 3GC-resistance rates of 35.7% in our study, which is consistent with the WHO GLASS report of 36.6% (interquartile range [IQR] 17.5-58.3) [37]. The fluoroquinolone-resistance rates of 22% that we observed were high but consistent with previous reports from Indonesia [73,74].

Bacteremia from *S. aureus* infection was found in 10.9% cases in our study, and the observed mortality rate of 20% was consistent with a previous report [75]. Both participants who died were diabetic and contracted oxacillin-sensitive infections, suggesting that the cause of death may have been due more to the timing of diagnosis and treatment. It is well-known that diabetics are at high risk for infections with *S. aureus* [76], so comorbidities should be strongly considered when prioritizing blood culture testing. Two participants with systemic lupus erythematosus (SLE) developed *S. aureus* BSIs, which has been associated with classic hyper-IgE syndrome [77]. The colonization of *S. aureus* in the body often increases in patients with SLE and may predispose them to BSI, worsening the SLE itself and leading to a feedback loop with the potential to reinforce autoimmune symptoms [78,79]. The proportion of MRSA in our study (10%) was lower than the WHO GLASS report (24.9% (IQR 11.4-42.7)) [37], though this is understandable given that our study was not a systematic surveillance of *S. aureus* infections across the country. Geographic variation of CAI with MRSA has been observed in the Asia-Pacific region, including Taiwan, the Philippines, Vietnam, and Sri Lanka (30-39%); Korea and Japan (15- 20%); and Thailand, India, and Hong Kong (3-9%) [80,81]. Data from Indonesia remains limited, but a recent

study has shown that the carriage rate of MRSA in the nose and throat of patients admitted to surgery and internal medical wards at Dr. Soetomo Hospital in Surabaya was 8.1% among 643 patients [82]. Additionally, a report on 259 *S. aureus* isolates collected from clinical cultures of patients at four tertiary care hospitals in Denpasar, Malang, Padang, and Semarang found that 6.6% and 18.5% were MRSA and PVL-positive methicillin-susceptible *S. aureus*, respectively [83].

Besides *E. coli* and *S. aureus*, we observed the other WHO GLASS pathogens *K. pneumonia, S. pneumonia*, and *Acinetobacter spp.* in our study. *K. pneumonia* was mostly found in patients with UTI and respiratory illnesses. The two fatal cases were most likely associated with the participants' chronic illnesses (stroke and kidney failure), as none of the isolates were 3GC, fluroquinolone, or co-trimoxazole resistant. Both cases of *S. pneumonia* bacteremia were found in pediatric participants, and both were fatal. The participant with a penicillin-sensitive infection had myelodysplasia syndrome, and the participant with a ceftriaxone-resistant infection had clinical meningitis. *S. pneumonia* was also found by molecular methods in 8 participants whose blood cultures were negative, supporting a previous report that successful diagnostic approaches using blood cultures alone are difficult because of reduced sensitivity[68]. [84]. *Acinetobacter lwoffii* was identified in two participants, both having gastro-intestinal symptoms and receiving an initial diagnosis of typhoid fever. Treatment with cefixime resolved the infections. A similar case with fever, abdominal pain, and diarrhea has been reported in a 64 year-old man in Texas, USA [6985].

Field Code Changed

Our study found the most frequent BSI pathogens to be *S.* Typhi and *E. coli*, though multidrug-resistant *E. coli* was the most problematic. The challenges of AMR in Indonesia are similar to those of many other low and middle-income countries in the region and globally [20]. Misuse and overuse of antibiotics in humans, livestock, and aquaculture may

be the key drivers of resistance in the country [86]. Despite current policies related to antimicrobial use in Indonesia, frequent and unnecessary prescription of antibiotics by physicians, high rates of self-medication, and over-the-counter access to antibiotics remain common [87]. Since 2016, the Indonesia Ministry of Health has boosted their AMR stewardship program to tackle this growing challenge, directing substantial funding to the national AMR control committee [20]. Further support for AMR prevention and the alignment of national policies with global policies and standards will substantially improve the growing challenge of AMR infections in Indonesia.

Our study has several limitations. First, the blood specimens analyzed as part of this study were collected only from a limited number of extremely ill patients admitted to tertiary hospitals. Blood culture positivity rates, AMR patterns, and clinical outcomes may not be generalizable to the Indonesian population at-large, though better understanding this critically ill population will hopefully lead to a reduction in mortality from BSIs. Second, only aerobic blood cultures were performed, which may have resulted in missed BSIs caused by anaerobic bacterial. The generally low yield of anaerobic bacteria combined with increasing costs and volumes of blood drawn ~~[13,72,73]~~[13,88,89] make anaerobic cultures impractical for many hospitals in Indonesia. In the future, rationally targeting the use of anaerobic culture bottles based on careful clinical assessment may result in substantial savings and facilitate the broader adoption of the diagnostic in the country ~~[74].~~[90]. Lastly, AMR susceptibility testing in this study was performed and reported according to general practice in Indonesia, as our study was not initially designed as an AMR study. Consequently, our data has substantial gaps and missing information. A standardized approach and electronic results reporting system in Indonesia would significantly improve the accuracy and utility of AMR susceptibility testing.

Conclusion

We presented aerobic blood culture findings from a multi-centre study of patients with acute febrile illness admitted to eight major hospitals across Indonesia. Our universal use of aerobic blood cultures is unique in Indonesia, the results of which help clarify the epidemiology and burden of BSI, rates of contamination among CAI, and common AMR patterns in Indonesia. Bacteremia was observed in 8.9% participants, with the most frequent pathogens being *Salmonella spp.*, *E. coli*, and *S. aureus*. Two *Salmonella spp.* cases had evidence of AMR, and several *E. coli* cases were multidrug resistant (42.9%) or monoresistant (14.3%). Culture contamination was observed in 3.6% cases. Our data suggest that blood cultures should be included as a routine diagnostic test, and pre-screening patients for the most common viral infections, such as dengue, influenza and chikungunya viruses, would conserve scarce resources without negatively impacting patient benefit. The routine practice of AMR susceptibility testing on positive blood cultures in Indonesia is encouraging and should be continued to inform clinical decisions on patient treatment in real-time. The country could benefit from clear guidance at the national level, particularly regarding the timing of blood collection prior to antibiotic administration, the prioritization of patients with comorbidities, blood collection practices to reduce environmental contamination, and the supplementation of blood cultures with molecular assays to combat false-negative results. Additionally, Indonesia could greatly benefit from a nationwide program for the systematic collection and dissemination of blood culture and AMR results.

Acknowledgements

We would like to thank all of the patients who participated in this study, the site study teams and investigators, US-NIAID and Indonesia NIHRD, the Indonesian Ministry of Health, the INA-RESPOND Network Steering Committee, and the sample repository team.

References

~~1~~1. David A. Smith, Sara M. Nehring. Bacteremia. Treasure Island (FL): StatPearls Publishing; 2022. Available: https://www.ncbi.nlm.nih.gov/books/NBK441979/

Formatted: Font: Calibri, English (United Kingdom)

2. Goto M, Al-Hasan MN. Overall burden of bloodstream infection and nosocomial bloodstream infection in North America and Europe. Clin Microbiol Infect. 2013;19: 501–509. doi:10.1111/1469-0691.12195

Formatted: Font: Calibri, English (United Kingdom)

3. McNamara JF, Righi E, Wright H, Hartel GF, Harris PNA, Paterson DL. Long-term morbidity and mortality following bloodstream infection: A systematic literature review. J Infect. 2018;77: 1–8. doi:10.1016/j.jinf.2018.03.005

Formatted: Font: Calibri, English (United Kingdom)

4. Peters RPH, Zijlstra EE, Schijffelen MJ, Walsh AL, Joaki G, Kumwenda JJ, et al. A prospective study of bloodstream infections as cause of fever in Malawi: clinical predictors and implications for management. Trop Med Int Health. 2004;9: 928–934. doi:10.1111/j.1365-3156.2004.01288.x

Formatted: Font: Calibri, English (United Kingdom)

~~2. David A. Smith, Sara M. Nehring. Bacteremia. Treasure Island (FL): StatPearls Publishing; 2022. Available: https://www.ncbi.nlm.nih.gov/books/NBK441979/~~

Formatted: Font: Calibri, English (United Kingdom)

~~3. Goto M, Al-Hasan MN. Overall burden of bloodstream infection and nosocomial bloodstream infection in North America and Europe. Clin Microbiol Infect. 2013;19: 501–509. doi:10.1111/1469-0691.12195~~

Formatted: Font: Calibri, English (United Kingdom)

~~4. McNamara JF, Righi E, Wright H, Hartel GF, Harris PNA, Paterson DL. Long-term morbidity and mortality following bloodstream infection: A systematic literature review. J Infect. 2018;77: 1–8. doi:10.1016/j.jinf.2018.03.005~~

Formatted: Font: Calibri, English (United Kingdom)

5. Gaibani P, Rossini G, Ambretti S, Gelsomino F, Pierro AM, Varani S, et al. Blood culture systems: rapid detection – how and why? Int J Antimicrob Agents. 2009;34: S13–S15. doi:10.1016/S0924-8579(09)70559-X

6. William A. Blood Culture Systems: From Patient to Result. In: Azevedo L, editor. Sepsis - An Ongoing and Significant Challenge. InTech; 2012. doi:10.5772/50139

7. Lamy B, Dargère S, Arendrup MC, Parienti J-J, Tattevin P. How to Optimize the Use of Blood Cultures for the Diagnosis of Bloodstream Infections? A State-of-the Art. Front Microbiol. 2016;7. doi:10.3389/fmicb.2016.00697

8. Prestinaci F, Pezzotti P, Pantosti A. Antimicrobial resistance: a global multifaceted phenomenon. Pathog Glob Health. 2015;109: 309–318. doi:10.1179/2047773215Y.0000000030

9. Hu B, Tao Y, Shao Z, Zheng Y, Zhang R, Yang X, et al. A Comparison of Blood Pathogen Detection Among Droplet Digital PCR, Metagenomic Next-Generation Sequencing, and Blood Culture in Critically Ill Patients With Suspected Bloodstream Infections. Front Microbiol. 2021;12: 641202. doi:10.3389/fmicb.2021.641202

10. Trung NT, Thau NS, Bang MH, Song LH. PCR-based Sepsis@Quick test is superior in comparison with blood culture for identification of sepsis-causative pathogens. Sci Rep. 2019;9: 13663. doi:10.1038/s41598-019-50150-y

11. Weinstein MP. Blood Culture Contamination: Persisting Problems and Partial Progress. J Clin Microbiol. 2003;41: 2275–2278. doi:10.1128/JCM.41.6.2275-2278.2003

12. Hall KK, Lyman JA. Updated Review of Blood Culture Contamination. Clin Microbiol Rev. 2006;19: 788–802. doi:10.1128/CMR.00062-05

13. Dargère S, Parienti J-J, Roupie E, Gancel P-E, Wiel E, Smaiti N, et al. Unique blood culture for diagnosis of bloodstream infections in emergency departments: a prospective multicentre study. Clin Microbiol Infect. 2014;20: O920–O927. doi:10.1111/1469-0691.12656

14. Bates DW. Predicting Bacteremia in Hospitalized Patients: A Prospectively Validated Model. Ann Intern Med. 1990;113: 495. doi:10.7326/0003-4819-113-7-495

15. Salluzzo R, Reilly K. The Rational Ordering of Blood Cultures in the Emergency Department. Qual Assur Util Rev. 1991;6: 28–31. doi:10.1177/0885713x9100600106

16. Little JR, Trovillion E, Fraser V. High Frequency of Pseudobacteremia at a University Hospital. Infect Control Hosp Epidemiol. 1997;18: 200–202. doi:10.1086/647588

17. Teerawattanasook N, Tauran PM, Teparrukkul P, Wuthiekanun V, Dance DAB, Arif M, et al. Capacity and Utilization of Blood Culture in Two Referral Hospitals in Indonesia and Thailand. Am J Trop Med Hyg. 2017;97: 1257–1261. doi:10.4269/ajtmh.17-0193

18. Doern GV, Carroll KC, Diekema DJ, Garey KW, Rupp ME, Weinstein MP, et al. Practical Guidance for Clinical Microbiology Laboratories: A Comprehensive Update on the Problem of Blood Culture Contamination and a Discussion of Methods for Addressing the Problem. Clin Microbiol Rev. 2019;33. doi:10.1128/CMR.00009-19

19. Gasem MH, Kosasih H, Tjitra E, Alisjahbana B, Karyana M, Lokida D, et al. An observational prospective cohort study of the epidemiology of hospitalized patients

with acute febrile illness in Indonesia. PLoS Negl Trop Dis. 2020;14: e0007927. doi:10.1371/journal.pntd.0007927

20. Parathon H, Kuntaman K, Widiastoety TH, Muliawan BT, Karuniawati A, Qibtiyah M, et al. Progress towards antimicrobial resistance containment and control in Indonesia. BMJ. 2017; j3808. doi:10.1136/bmj.j3808

~~21~~21. Gasem MH, Wagenaar JFP, Goris MGA, Adi MS, Isbandrio BB, Hartskeerl RA, et al. Murine typhus and leptospirosis as causes of acute undifferentiated fever, Indonesia. Emerg Infect Dis. 2009;15: 975–977. doi:10.3201/eid1506.081405

22. Capeding MR, Chua MN, Hadinegoro SR, Hussain IIHM, Nallusamy R, Pitisuttithum P, et al. Dengue and other common causes of acute febrile illness in Asia: an active surveillance study in children. PLoS Negl Trop Dis. 2013;7: e2331. doi:10.1371/journal.pntd.0002331

23. Punjabi NH, Taylor WRJ, Murphy GS, Purwaningsih S, Picarima H, Sisson J, et al. Etiology of acute, non-malaria, febrile illnesses in Jayapura, northeastern Papua, Indonesia. Am J Trop Med Hyg. 2012;86: 46–51. doi:10.4269/ajtmh.2012.10-0497

24. Kosasih H, de Mast Q, Widjaja S, Sudjana P, Antonjaya U, Ma'roef C, et al. Evidence for endemic chikungunya virus infections in Bandung, Indonesia. PLoS Negl Trop Dis. 2013;7: e2483. doi:10.1371/journal.pntd.0002483

25. Utama IMS, Lukman N, Sukmawati DD, Alisjahbana B, Alam A, Murniati D, et al. Dengue viral infection in Indonesia: Epidemiology, diagnostic challenges, and mutations from an observational cohort study. Messer WB, editor. PLoS Negl Trop Dis. 2019;13: e0007785. doi:10.1371/journal.pntd.0007785

26. Kosasih H, Alisjahbana B, Nurhayati null, de Mast Q, Rudiman IF, Widjaja S, et al. The Epidemiology, Virology and Clinical Findings of Dengue Virus Infections in a Cohort of Indonesian Adults in Western Java. PLoS Negl Trop Dis. 2016;10: e0004390. doi:10.1371/journal.pntd.0004390

27. Pranata IWA, Diana A, Heryanto MR, Lukman N, Kosasih H, Djauhari H, et al. Persistence of anti-Salmonella O9 IgM as measured by Tubex® TF may contribute to the over-diagnosis of typhoid fever in endemic areas. Bali Med J. 2022;11: 11. doi:10.15562/bmj.v11i1.3035

28. Zeng Z, Zhan J, Chen L, Chen H, Cheng S. Global, regional, and national dengue burden from 1990 to 2017: A systematic analysis based on the global burden of disease study 2017. EClinicalMedicine. 2021;32: 100712. doi:10.1016/j.eclinm.2020.100712

29. Harapan H, Michie A, Yohan B, Shu P-Y, Mudatsir M, Sasmono RT, et al. Dengue viruses circulating in Indonesia: A systematic review and phylogenetic analysis of data from five decades. Rev Med Virol. 2019;29: e2037. doi:10.1002/rmv.2037

Formatted: Font: Calibri, English (United Kingdom)

30. Harapan H, Michie A, Mudatsir M, Sasmono RT, Imrie A. Epidemiology of dengue hemorrhagic fever in Indonesia: analysis of five decades data from the National Disease Surveillance. BMC Res Notes. 2019;12: 350. doi:10.1186/s13104-019-4379-9

31. Kosasih H, Roselinda null, Nurhayati null, Klimov A, Xiyan X, Lindstrom S, et al. Surveillance of influenza in Indonesia, 2003–2007. Influenza Other Respir Viruses. 2013;7: 312–320. doi:10.1111/j.1750-2659.2012.00403.x

32. Kosasih H, Widjaja S, Surya E, Hadiwijaya SH, Butarbutar DPR, Jaya UA, et al. Evaluation of two IgM rapid immunochromatographic tests during circulation of Asian lineage Chikungunya virus. Southeast Asian J Trop Med Public Health. 2012;43: 55–61.

33. Centers for Disease Control and Prevention. Influenza (Flu). Guidance: Use of Rapid Diagnostic Test. 2016. Available: https://www.cdc.gov/flu/professionals/diagnosis/clinician_guidance_ridt.htm#:~:text=Sensitivities%20of%20RIDTs%20are%20generally,commonly%20than%20false%20positive%20results.

34. Chaterji S, Allen JC, Chow A, Leo Y-S, Ooi E-E. Evaluation of the NS1 rapid test and the WHO dengue classification schemes for use as bedside diagnosis of acute dengue fever in adults. Am J Trop Med Hyg. 2011;84: 224–228. doi:10.4269/ajtmh.2011.10-0316

35. Gonzalez MD, Chao T, Pettengill MA. Modern Blood Culture: Management Decisions and Method Options. Clin Lab Med. 2020;40: 379–392. doi:10.1016/j.cll.2020.07.001

~~22~~36. Brisse S, Stefani S, Verhoef J, Van Belkum A, Vandamme P, Goessens W. Comparative evaluation of the BD Phoenix and VITEK 2 automated instruments for identification of isolates of the Burkholderia cepacia complex. J Clin Microbiol. 2002;40: 1743–1748. doi:10.1128/JCM.40.5.1743-1748.2002

~~23~~37. World Health Organization. Global antimicrobial resistance surveillance system: manual for early implementation. 2015. Available: http://www.who.int/antimicrobial-resistance/publications/surveillance-system-manual/en/

~~24~~38. Vincent J-L, Marshall JC, Namendys-Silva SA, François B, Martin-Loeches I, Lipman J, et al. Assessment of the worldwide burden of critical illness: the intensive care over nations (ICON) audit. Lancet Respir Med. 2014;2: 380–386. doi:10.1016/S2213-2600(14)70061-X

~~25~~39. Fleischmann C, Scherag A, Adhikari NKJ, Hartog CS, Tsaganos T, Schlattmann P, et al. Assessment of Global Incidence and Mortality of Hospital-treated Sepsis. Current Estimates and Limitations. Am J Respir Crit Care Med. 2016;193: 259–272. doi:10.1164/rccm.201504-0781OC

~~26. Utama IMS, Lukman N, Sukmawati DD, Alisjahbana B, Alam A, Murniati D, et al.~~ 40~~Dengue viral infection in Indonesia: Epidemiology, diagnostic challenges, and mutations from an observational cohort study. Messer WB, editor. PLoS Negl Trop Dis. 2019;13: e0007785. doi:10.1371/journal.pntd.0007785~~

Formatted: Font: Calibri, English (United Kingdom)

Formatted: Font: Calibri, English (United Kingdom)

Formatted: Font: Calibri, English (United Kingdom)

Formatted: Font: Calibri, English (United Kingdom)

Formatted: Font: Calibri, English (United Kingdom)

Formatted: Font: Calibri, English (United Kingdom)

~~27~~. Trunfio M, Savoldi A, Viganò O, d'Arminio Monforte A. Bacterial coinfections in dengue virus disease: what we know and what is still obscure about an emerging concern. Infection. 2017;45: 1–10. doi:10.1007/s15010-016-0927-6

Formatted: Font: Calibri, English (United Kingdom)

~~28~~41. Thein T-L, Ng E-L, Yeang MS, Leo Y-S, Lye DC. Risk factors for concurrent bacteremia in adult patients with dengue. J Microbiol Immunol Infect. 2017;50: 314–320. doi:10.1016/j.jmii.2015.06.008

42. See KC, Phua J, Yip HS, Yeo LL, Lim TK. Identification of concurrent bacterial infection in adult patients with dengue. Am J Trop Med Hyg. 2013;89: 804–810. doi:10.4269/ajtmh.13-0197

43. Sudjana P, Jusuf H. Concurrent dengue hemorrhagic fever and typhoid fever infection in adult: case report. Southeast Asian J Trop Med Public Health. 1998;29: 370–372.

44. Lee A, Mirrett S, Reller LB, Weinstein MP. Detection of Bloodstream Infections in Adults: How Many Blood Cultures Are Needed? J Clin Microbiol. 2007;45: 3546–3548. doi:10.1128/JCM.01555-07

Formatted: Font: Calibri, English (United Kingdom)

~~29~~45. Wilson ML, Clinical and Laboratory Standards Institute. Principles and procedures for blood cultures: approved guideline. Wayne, Pa.: Clinical and Laboratory Standards Institute; 2007.

Formatted: Font: Calibri, English (United Kingdom)

~~30~~46. Murni IK, Duke T, Daley AJ, Kinney S, Soenarto Y. True Pathogen or Contamination: Validation of Blood Cultures for the Diagnosis of Nosocomial Infections in a Developing Country. J Trop Pediatr. 2018;64: 389–394. doi:10.1093/tropej/fmx081

Formatted: Font: Calibri, English (United Kingdom)

~~31~~47. Rhodes J, Jorakate P, Makprasert S, Sangwichian O, Kaewpan A, Akarachotpong T, et al. Population-based bloodstream infection surveillance in rural Thailand, 2007–2014. BMC Public Health. 2019;19: 521. doi:10.1186/s12889-019-6775-4

Formatted: Font: Calibri, English (United Kingdom)

~~32~~48. Chang C-J, Wu C-J, Hsu H-C, Wu C-H, Shih F-Y, Wang S-W, et al. Factors Associated with Blood Culture Contamination in the Emergency Department: Critical Illness, End-Stage Renal Disease, and Old Age. Lazzeri C, editor. PLOS ONE. 2015;10: e0137653. doi:10.1371/journal.pone.0137653

Formatted: Font: Calibri, English (United Kingdom)

~~33~~49. Scheer CS, Fuchs C, Gründling M, Vollmer M, Bast J, Bohnert JA, et al. Impact of antibiotic administration on blood culture positivity at the beginning of sepsis: a prospective clinical cohort study. Clin Microbiol Infect. 2019;25: 326–331. doi:10.1016/j.cmi.2018.05.016

Formatted: Font: Calibri, English (United Kingdom)

~~34~~50. Rand KH, Beal SG, Rivera K, Allen B, Payton T, Lipori GP. Hourly Effect of Pretreatment With IV Antibiotics on Blood Culture Positivity Rate in Emergency Department Patients. Open Forum Infect Dis. 2019;6: ofz179. doi:10.1093/ofid/ofz179

Formatted: Font: Calibri, English (United Kingdom)

~~35~~51. Limato R, Nelwan EJ, Mudia M, de Brabander J, Guterres H, Enty E, et al. A multicentre point prevalence survey of patterns and quality of antibiotic prescribing in Indonesian hospitals. JAC-Antimicrob Resist. 2021;3: dlab047. doi:10.1093/jacamr/dlab047

Formatted: Font: Calibri, English (United Kingdom)

~~36~~52. She RC, Bender JM. Advances in Rapid Molecular Blood Culture Diagnostics: Healthcare Impact, Laboratory Implications, and Multiplex Technologies. J Appl Lab Med. 2019;3: 617–630. doi:10.1373/jalm.2018.027409

Formatted: Font: Calibri, English (United Kingdom)

~~37~~53. Harris AM, Bramley AM, Jain S, Arnold SR, Ampofo K, Self WH, et al. Influence of Antibiotics on the Detection of Bacteria by Culture-Based and Culture-Independent Diagnostic Tests in Patients Hospitalized With Community-Acquired Pneumonia. Open Forum Infect Dis. 2017;4: ofx014. doi:10.1093/ofid/ofx014

Formatted: Font: Calibri, English (United Kingdom)

~~38~~54. Jones GR, Lowes JA. The systemic inflammatory response syndrome as a predictor of bacteraemia and outcome from sepsis. QJM. 1996;89: 515–522. doi:10.1093/qjmed/89.7.515

Formatted: Font: Calibri, English (United Kingdom)

~~39~~55. Shapiro NI, Wolfe RE, Wright SB, Moore R, Bates DW. Who needs a blood culture? A prospectively derived and validated prediction rule. J Emerg Med. 2008;35: 255–264. doi:10.1016/j.jemermed.2008.04.001

Formatted: Font: Calibri, English (United Kingdom)

~~40~~56. Seymour CW, Liu VX, Iwashyna TJ, Brunkhorst FM, Rea TD, Scherag A, et al. Assessment of Clinical Criteria for Sepsis: For the Third International Consensus Definitions for Sepsis and Septic Shock (Sepsis-3). JAMA. 2016;315: 762. doi:10.1001/jama.2016.0288

Formatted: Font: Calibri, English (United Kingdom)

~~41~~57. Ombelet S, Barbé B, Affolabi D, Ronat J-B, Lompo P, Lunguya O, et al. Best Practices of Blood Cultures in Low- and Middle-Income Countries. Front Med. 2019;6: 131. doi:10.3389/fmed.2019.00131

Formatted: Font: Calibri, English (United Kingdom)

~~42~~58. Seigel TA, Cocchi MN, Salciccioli J, Shapiro NI, Howell M, Tang A, et al. Inadequacy of Temperature and White Blood Cell Count in Predicting Bacteremia in Patients with Suspected Infection. J Emerg Med. 2012;42: 254–259. doi:10.1016/j.jemermed.2010.05.038

Formatted: Font: Calibri, English (United Kingdom)

~~43~~59. Ochiai RL. a study of typhoid fever in five Asian countries: disease burden and implications for controls. Bull World Health Organ. 2008;86: 260–268. doi:10.2471/BLT.06.039818

Formatted: Font: Calibri, English (United Kingdom)

~~44~~60. Nuruzzaman H, Syahrul F. Risk Analysis of Typhoid Fever Based on Personal Hygiene and Street Food Consumption Habit at Home. J Berk Epidemiol. 2016;4: 74–86.

Formatted: Font: Calibri, English (United Kingdom)

~~45~~61. Mogasale V, Ramani E, Mogasale VV, Park J. What proportion of Salmonella Typhi cases are detected by blood culture? A systematic literature review. Ann Clin Microbiol Antimicrob. 2016;15: 32. doi:10.1186/s12941-016-0147-z

Formatted: Font: Calibri, English (United Kingdom)

~~46~~62. Andrews JR, Ryan ET. Diagnostics for invasive Salmonella infections: Current challenges and future directions. Vaccine. 2015;33: C8–C15. doi:10.1016/j.vaccine.2015.02.030

Formatted: Font: Calibri, English (United Kingdom)

~~47~~63. Keddy K, Sooka A, Letsoalo M, Hoyland G, Chaignat CL, Morrissey A, et al. Sensitivity and specificity of typhoid fever rapid antibody tests for laboratory diagnosis at two

Formatted: Font: Calibri, English (United Kingdom)

sub-Saharan African sites. Bull World Health Organ. 2011;89: 640–647. doi:10.2471/BLT.11.087627

~~48~~64. Hardjo Lugito NP, Cucunawangsih. Antimicrobial Resistance of *Salmonella enterica* Serovars Typhi and Paratyphi Isolates from a General Hospital in Karawaci, Tangerang, Indonesia: A Five-Year Review. Int J Microbiol. 2017;2017: 1–7. doi:10.1155/2017/6215136

~~49~~65. Chiou C-S, Lauderdale T-L, Phung DC, Watanabe H, Kuo J-C, Wang P-J, et al. Antimicrobial Resistance in Salmonella enterica Serovar Typhi Isolates from Bangladesh, Indonesia, Taiwan, and Vietnam. Antimicrob Agents Chemother. 2014;58: 6501–6507. doi:10.1128/AAC.03608-14

66. Punjabi NH, Agtini MD, Ochiai RL, Simanjuntak CH, Lesmana M, Subekti D, et al. Enteric fever burden in North Jakarta, Indonesia: a prospective, community-based study. J Infect Dev Ctries. 2013;7: 781–787. doi:10.3855/jidc.2629

~~50~~67. ~~Chiou C-S, Lauderdale T-L, Phung DC, Watanabe H, Kuo J-C, Wang P-J, et al. Antimicrobial Resistance in Salmonella enterica Serovar Typhi Isolates from Bangladesh, Indonesia, Taiwan, and Vietnam. Antimicrob Agents Chemother. 2014;58: 6501–6507. doi:10.1128/AAC.03608-14~~

~~51~~. Crump JA, Sjölund-Karlsson M, Gordon MA, Parry CM. Epidemiology, Clinical Presentation, Laboratory Diagnosis, Antimicrobial Resistance, and Antimicrobial Management of Invasive Salmonella Infections. Clin Microbiol Rev. 2015;28: 901–937. doi:10.1128/CMR.00002-15

~~52~~68. Abernethy J, Guy R, Sheridan EA, Hopkins S, Kiernan M, Wilcox MH, et al. Epidemiology of Escherichia coli bacteraemia in England: results of an enhanced sentinel surveillance programme. J Hosp Infect. 2017;95: 365–375. doi:10.1016/j.jhin.2016.12.008

~~53~~69. Bou-Antoun S, Davies J, Guy R, Johnson AP, Sheridan EA, Hope RJ. Descriptive epidemiology of Escherichia coli bacteraemia in England, April 2012 to March 2014. ~~Euro Surveill Bull Eur Sur Mal Transm Eur Commun Dis Bull.~~Eurosurveillance. 2016;21. doi:10.2807/1560-7917.ES.2016.21.35.30329

~~54~~70. Williamson DA, Lim A, Wiles S, Roberts SA, Freeman JT. Population-based incidence and comparative demographics of community-associated and healthcare-associated Escherichia coli bloodstream infection in Auckland, New Zealand, 2005~~-~~–2011. BMC Infect Dis. 2013;13: 385. doi:10.1186/1471-2334-13-385

~~55~~71. Diekema DJ, Hsueh P-R, Mendes RE, Pfaller MA, Rolston KV, Sader HS, et al. The Microbiology of Bloodstream Infection: 20-Year Trends from the SENTRY Antimicrobial Surveillance Program. Antimicrob Agents Chemother. 2019;63. doi:10.1128/AAC.00355-19

Formatted: Font: Calibri, English (United Kingdom)

Formatted: Font: Calibri, English (United Kingdom)

Formatted: Font: Calibri, English (United Kingdom)

Formatted: Font: Calibri, English (United Kingdom)

Formatted: Font: Calibri, English (United Kingdom)

Formatted: Font: Calibri, English (United Kingdom)

Formatted: Font: Calibri, English (United Kingdom)

Formatted: Font: Calibri, English (United Kingdom)

Formatted: Font: Calibri, English (United Kingdom)

Formatted: Font: Calibri, English (United Kingdom)

Formatted: Font: Calibri, English (United Kingdom)

~~56~~72. Peirano G, Pitout JDD. Extended-Spectrum β-Lactamase-Producing Enterobacteriaceae: Update on Molecular Epidemiology and Treatment Options. Drugs. 2019;79: 1529–1541. doi:10.1007/s40265-019-01180-3

Formatted: Font: Calibri, English (United Kingdom)

~~57~~73. Dahesihdewi A, Sugianli AK, Parwati I. The surveillance of antibiotics resistance in Indonesia: a current reports. Bali Med J. 2019;8: 565. doi:10.15562/bmj.v8i2.1386

Formatted: Font: Calibri, English (United Kingdom)

~~58~~74. Lestari ES, Severin JA, Filius PMG, Kuntaman K, Duerink DO, Hadi U, et al. Antimicrobial resistance among commensal isolates of Escherichia coli and Staphylococcus aureus in the Indonesian population inside and outside hospitals. Eur J Clin Microbiol Infect Dis. 2007;27: 45–51. doi:10.1007/s10096-007-0396-z

Formatted: Font: Calibri, English (United Kingdom)

~~59~~75. van Hal SJ, Jensen SO, Vaska VL, Espedido BA, Paterson DL, Gosbell IB. Predictors of Mortality in Staphylococcus aureus Bacteremia. Clin Microbiol Rev. 2012;25: 362–386. doi:10.1128/CMR.05022-11

Formatted: Font: Calibri, English (United Kingdom)

~~60~~76. Hansen M-LU, Gotland N, Mejer N, Petersen A, Larsen AR, Benfield T, et al. Diabetes increases the risk of disease and death due to *Staphylococcus aureus* bacteremia. A matched case-control and cohort study. Infect Dis. 2017;49: 689–697. doi:10.1080/23744235.2017.1331463

Formatted: Font: Calibri, English (United Kingdom)

~~61~~77. Schopfer K, Feldges A, Baerlocher K, Parisot RF, Wilhelm JA, Matter L. Systemic lupus erythematosus in Staphylococcus aureus hyperimmunoglobulinaemia E syndrome. BMJ. 1983;287: 524–526. doi:10.1136/bmj.287.6391.524

Formatted: Font: Calibri, English (United Kingdom)

~~62~~78. Battaglia M, Garrett-Sinha LA. Bacterial infections in lupus: Roles in promoting immune activation and in pathogenesis of the disease. J Transl Autoimmun. 2021;4: 100078. doi:10.1016/j.jtauto.2020.100078

Formatted: Font: Calibri, English (United Kingdom)

~~63~~79. Sirobhushanam S, Parsa N, Reed TJ, Berthier CC, Sarkar MK, Hile GA, et al. Staphylococcus aureus Colonization Is Increased on Lupus Skin Lesions and Is Promoted by IFN-Mediated Barrier Disruption. J Invest Dermatol. 2020;140: 1066-1074.e4. doi:10.1016/j.jid.2019.11.016

Formatted: Font: Calibri, English (United Kingdom)

~~64~~80. Song J-H, Hsueh P-R, Chung DR, Ko KS, Kang C-I, Peck KR, et al. Spread of methicillin-resistant Staphylococcus aureus between the community and the hospitals in Asian countries: an ANSORP study. J Antimicrob Chemother. 2011;66: 1061–1069. doi:10.1093/jac/dkr024

Formatted: Font: Calibri, English (United Kingdom)

~~65~~81. Chuang Y-Y, Huang Y-C. Molecular epidemiology of community-associated meticillin-resistant Staphylococcus aureus in Asia. Lancet Infect Dis. 2013;13: 698–708. doi:10.1016/S1473-3099(13)70136-1

Formatted: Font: Calibri, English (United Kingdom)

~~66~~82. Kuntaman K, Hadi U, Setiawan F, Koendori EB, Rusli M, Santosaningsih D, et al. ~~PREVALENCE OF METHICILLIN RESISTANT STAPHYLOCOCCUS AUREUS FROM NOSE AND THROAT OF PATIENTS ON ADMISSION TO MEDICAL WARDS OF DR SOETOMO HOSPITAL, SURABAYA, INDONESIA.~~Prevalence of methicillin resistant *Staphylococcus aureus* from nose and throat of patients on admission to medical wards of Dr Soetomo

Formatted: Font: Calibri, English (United Kingdom)

Hospital, Surabaya, Indonesia. Southeast Asian J Trop Med Public Health. 2016;47: 66–70.

Formatted: Font: Calibri, English (United Kingdom)

~~67~~83. Santosaningsih D, Santoso S, Budayanti NS, Suata K, Lestari ES, Wahjono H, et al. Characterisation of clinical *Staphylococcus aureus* isolates harbouring *mecA* or Panton-Valentine leukocidin genes from four tertiary care hospitals in Indonesia. Trop Med Int Health. 2016;21: 610–618. doi:10.1111/tmi.12692

Formatted: Font: Calibri, English (United Kingdom)

~~68~~84. Korsgaard J, Møller JK, Kilian M. Antibiotic treatment and the diagnosis of Streptococcus pneumoniae in lower respiratory tract infections in adults. Int J Infect Dis. 2005;9: 274–279. doi:10.1016/j.ijid.2004.07.013

Formatted: Font: Calibri, English (United Kingdom)

~~69~~85. Regalado NG, Martin G, Antony SJ. Acinetobacter lwoffii: Bacteremia associated with acute gastroenteritis. Travel Med Infect Dis. 2009;7: 316–317. doi:10.1016/j.tmaid.2009.06.001

Formatted: Font: Calibri, English (United Kingdom)

~~70~~86. Van Boeckel TP, Brower C, Gilbert M, Grenfell BT, Levin SA, Robinson TP, et al. Global trends in antimicrobial use in food animals. Proc Natl Acad Sci. 2015;112: 5649–5654. doi:10.1073/pnas.1503141112

Formatted: Font: Calibri, English (United Kingdom)

~~71~~87. Hadi U, Duerink DO, Lestari ES, Nagelkerke NJ, Werter S, Keuter M, et al. Survey of antibiotic use of individuals visiting public healthcare facilities in Indonesia. Int J Infect Dis. 2008;12: 622–629. doi:10.1016/j.ijid.2008.01.002

Formatted: Font: Calibri, English (United Kingdom)

~~72~~88. Kirn TJ, Weinstein MP. Update on blood cultures: how to obtain, process, report, and interpret. Clin Microbiol Infect. 2013;19: 513–520. doi:10.1111/1469-0691.12180

Formatted: Font: Calibri, English (United Kingdom)

~~73~~89. Bartlett JG, Dick J. The controversy regarding routine anaerobic blood cultures. Am J Med. 2000;108: 505–506. doi:10.1016/S0002-9343(00)00321-1

Formatted: Font: Calibri, English (United Kingdom)

~~74~~90. Lafaurie M, d'Anglejan E, Donay JL, Glotz D, Sarfati E, Mimoun M, et al. Utility of anaerobic bottles for the diagnosis of bloodstream infections. BMC Infect Dis. 2020;20: 142. doi:10.1186/s12879-020-4854-x

Formatted: Font: Calibri, English (United Kingdom)

Comments to the Author

1. Is the manuscript technically sound, and do the data support the conclusions?

The manuscript must describe a technically sound piece of scientific research with data that supports the conclusions. Experiments must have been conducted rigorously, with appropriate controls, replication, and sample sizes. The conclusions must be drawn appropriately based on the data presented.
Reviewer #1: Yes
Reviewer #2: Yes

____

2. Has the statistical analysis been performed appropriately and rigorously?
Reviewer #1: Yes
Reviewer #2: Yes

____

3. Have the authors made all data underlying the findings in their manuscript fully available?

The PLOS Data policy requires authors to make all data underlying the findings described in their manuscript fully available without restriction, with rare exception (please refer to the Data Availability Statement in the manuscript PDF file). The data should be provided as part of the manuscript or its supporting information, or deposited to a public repository. For example, in addition to summary statistics, the data points behind means, medians and variance measures should be available. If there are restrictions on publicly sharing data—e.g. participant privacy or use of data from a third party—those must be specified.
Reviewer #1: Yes
Reviewer #2: Yes

____

4. Is the manuscript presented in an intelligible fashion and written in standard English?

PLOS ONE does not copyedit accepted manuscripts, so the language in submitted articles must be clear, correct, and unambiguous. Any typographical or grammatical errors should be corrected at revision, so please note any specific errors here.
Reviewer #1: Yes
Reviewer #2: No

____

5. Review Comments to the Author

Please use the space provided to explain your answers to the questions above. You may also include additional comments for the author, including concerns about dual publication, research ethics, or publication ethics. (Please upload your review as an attachment if it exceeds 20,000 characters)

Reviewer #1: This compilation of data from different centers over many years is commendable. This highlights the issues faced in diagnostic microbiology in developing countries. It is an interesting paper with important observations and discussions. Some spellings need review and correction. Recommend to submit after corrections.

Response: Thank you very much for your comments, we really appreciate it.
We have corrected the spelling errors.

Reviewer #2: The Characteristics of Bacteremia among Patients with Acute Febrile Illness Requiring Hospitalization in Indonesia.

Evaluation. This report addresses an important subject in Bacteriemia and Acute Febrile illness; i.e., the worrying trend of antimicrobial resistance in bacterial pathogens (Salmonella and Non Salmonella spp) . It reports the frequency and distribution of bacterial pathogens in blood culture and its susceptibility pattern isolated from various specimens from a seven medical center in Indonesia, from which similar reports are scarce. Though it is better attempt by Soedarmono et al., to know information on bacteremia and other causative agent of Acute Febrile illeness in Indonesia.

Response: Thank you very much for your comments, we really appreciate it.

Comments

1.Give rationale of the study? Why is NS1 antigen screening only performed? What about other viral agents related AFI?

Response: We have added more information regarding this issue in the Methods.

Lines 135-146 now read: During the baseline visit, blood was collected for cultures, clinically relevant rapid diagnostic tests when available, and dengue virus rapid diagnostic tests. Dengue virus infection remains a significant burden across Indonesia [28,29], with disease incidence increasing in recent years [30]. Though other viral agents are present in Indonesia, none are as prevalent as dengue virus [24,31], and most are challenging to diagnose due to limitations with available rapid diagnostic tests [32,33]. Given the widespread prevalence of dengue virus infection, and the very high specificity (almost 100%) and good sensitivity (70-87%) of NS1 antigen rapid diagnostic tests [34], we employed universal dengue virus screening to rapidly resolve the unknown etiologies of study participants. Participants with negative NS1 antigen tests were further considered for BSIs through blood culture tests and other etiologies, as determined through advanced testing at the INA-RESPOND reference laboratory.

2.Why you performed Blood culture 0f 1459 Cases? You have mentioned 1464 were enrolled? What about 5??

Response: We only performed blood culture for 1459 patients, as the remaining 5 subjects did not have enough blood for blood culture test.

Lines 207-210 now read: The remaining 5 participants had insufficient blood specimens for following reasons: 1 adult was in a severe condition (decreased of consciousness), 2 participants (1 child and 1 adult) self-discharged against medical advice, and the guardians of 2 children refused to allow more blood to be drawn.

3.At the end of introduction, please give some update of Acute Febrile illness and their epidemiology in Indonesia.

Response: Thank you very much for the suggestion. We have added some update of acute febrile illness and their epidemiology in Indonesia.

Lines 97-111 now read: The epidemiology of pathogens associated with fever in Indonesia is not well understood, as public health surveillance data is limited and only a few local studies have been conducted [19,21–26]. Among published studies, dengue virus, chikungunya virus, influenza virus, *Salmonella* Typhi, *Rickettsia spp.*, and *Leptospira spp.* are consistently the most common causes of acute febrile illness hospitalizations. A study in Papua from November 1997 to February 2000 enrolled 226 hospitalized patients that were negative for malaria, the majority of whom were determined to have typhoid fever (18%), leptospirosis (12%), rickettsioses (8%), and dengue fever (7%) [23]. An observational fever study in Bandung identified dengue virus in 12.4% of fever episodes, followed by *S.* Typhi (7.4%), and chikungunya virus (7.1%) [24,26,27]. A 2005-2006 study in Semarang found rickettsioses and leptospirosis in 7% and 10%, respectively, of 137 acute undifferentiated fever cases [21]. The parent study of the research presented here found the most prevalent pathogens among participants at eight hospitals in 7 major cities in Indonesia to be dengue virus (27-52%), *Rickettsia spp.* (2-12%), *S.* Typhi (0.9-13%), influenza virus (2-6%), *Leptospira spp.* (0-5%), and chikungunya virus (0-4%) [19].

4.Which are the hospitals included in the study, please mentions the name of hospitals.

Response: We have included the name of hospitals in the Methods.

Lines 121-127 now read: A prospective observational study enrolling febrile patients who required hospitalization was conducted by the Indonesia Research Partnership on Infectious Disease (INA-RESPOND) from July 2013 to June 2016 at eight major hospitals in seven provincial capitals in Indonesia: Dr. Cipto Mangunkusumo Hospital in Jakarta, Sulianti Saroso Infectious Disease Hospital in Jakarta, Dr. Wahidin Sudirohusodo Hospital in Makassar, Dr. Sardjito Hospital in Yogyakarta, Dr. Hasan Sadikin Hospital in Bandung, Sanglah General Hospital in Denpasar, Dr. Soetomo Hospital in Surabaya, and Dr. Kariadi Hospital, in Semarang.

5.How do you calculate sample size? Is it sufficient to draw conclusion regarding bacteremia (causative bacterial pathogens) in Indonesia?

Response: As this study was an observational study to find etiologies of acute febrile illness during a certain period of time (2013-2016), we did not specifically calculate the sample size for drawing the conclusion regarding bacteremia in Indonesia. Since we performed the analysis of blood culture results from almost all participants (>99% participants, approximately 100 adults and 100 children from each hospital), though cannot be generalizable to the Indonesian population at-large, we expected that the data will provide better understanding of the bacteremia in hospitalized population with fever and hopefully will lead to a reduction in mortality from BSIs.

6.What is your inclusion and exclusion criteria? Please mention Clearly.

Response: We have added the inclusion and exclusion criteria.

Lines 128-131 now read: Briefly, inclusion criteria consisted of axillary body temperature ≥38˚C, ≥1 year of age, and hospitalization within the past 24 hours. Patients were excluded from the study if they had subjective fever for ≥14 days or were hospitalized in the last 3 months.

7.Please give the ethical approval committee name and approval number and date.

Response: The name of the ethical approval committee and approval number had already provided under the "Ethical Clearance" (lines 197-203); and we have added the date.

Ethical approvals for the AFIRE study were granted by the Institutional Review Boards of the National Institute of Health Research and Development (NIHRD), Indonesia Ministry of Health (KE.01.05/EC/407/2012) dated 23 May 2012, the Faculty of Medicine at the University of Indonesia and RSUPN Dr. Cipto Mangunkusumo Hospital (451/PT02.FK/ETIK/2012) dated 23 July 2012, and RSUD Dr. Soetomo Hospital (192/Panke.KKE/VIII/2012) dated 13 August 2012.

8.How do assure the Quality controls and quality check of your results, either BD 135 Phoenix (Becton Dickinson) or VITEK 2 (bioMérieux, Inc., Durham, North Carolina), System?

Response: Blood culture tests were performed at the hospital's accredited clinical laboratory, which provides patient diagnostic services. All instruments and standards were calibrated appropriately according to manufacturer guidelines. Every site's laboratory performed quality control (QC) to ensure proper performance and sent the QC report to protocol team to be reviewed. All tests were run alongside appropriate positive and negative control to ensure the integrity and accuracy of the results. For example, QC for VITEK 2 system; each new lot number of ID cards is tested with stock culture organisms. Susceptibility cards are tested weekly against stock culture organisms.

The QC organisms uses as follows:

<u>Weekly:</u>
AST-GP 67 cards
    Enterococcus faecalis ATCC 29212
AST-GN 66 cards
    E. coli ATCC 25922 non fermenter
    PSA ATCC 27853 fermenter
    E. coli ATCC 35218 non fermenter
ID-NH cards
    Elkenella corrodens ATCC BAA-1152
<u>New Lots:</u>
ID-GP cards

Enterococcus casseliflavis ATCC 700327

ID-GN cards

Enterobacter hormechei (*E.cloacae*) ATCC 700323

Lines 163-171 now read: Blood cultures were performed and analyzed at the hospitals' nationally accredited clinical laboratories by trained, certified staff. All instruments and standards were calibrated appropriately according to manufacturer guidelines, and all tests were run alongside appropriate positive and negative control to ensure the integrity and accuracy of the results. Organism identification was considered acceptable when the confidence level in the automated growth identification system was ≥95% probability [34]. Quality control tests were performed weekly at all site laboratories, and each new lot of ID cards was tested using validated stocks of culture organisms.

9.What is the volume of blood sample collected and used in culture from children and adults?

Response: This is already stated in the text. Blood volumes of approximately 5-8 mL for adults and 1-3 mL for children were collected from each arm, whenever possible, directly into separate aerobic blood culture bottles (lines 150-152).

10.It is better to give numerator value after percentage values.

Response: We have changed the presentation throughout the manuscript.

11.Please give the full name of bacteria initially such as Staphylococcus aureus and then short form S. aureus and other bacteria throughout the manuscript.

Response: We have followed your suggestion.

12.Please mention the more information on infections with dengue virus and bacteremia in Indonesia.

Response: We found no dengue virus and bacteremia co-infection in our study, as mentioned in the Discussion. We have added more informations about dengue virus and bacteremia.

Lines 355-368 now read: Data on co-infections with dengue virus and bacteremia is limited. A literature review of published case reports and studies from January 1943 to March 2016 found 3 studies in Singapore and Taiwan reporting concurrent bacteremia in 0.18-7% of dengue fever cases [40–42]. A concurrent dengue virus and *S.* Typhi case was also reported from Bandung, Indonesia [43]. In all of these studies, blood was collected for bacterial culture because patients did not improve clinically a few days to a week after dengue fever was diagnosed. Furthermore, in the majority of cases, dengue virus infection was confirmed by serology only (IgM detected or four-fold IgG increase). These reports support our finding that simultaneous infection with bacteria and dengue virus is rare. In our study, bacterial growth observed in 14 participants with positive dengue NS1 antigen tests were considered false positive blood cultures (5 *Staphylococcus hominis*, 4 *Staphylococcus epidermidis*, 1

Kocuria rosea, 1 *Micrococcus aureus*, 1 *Staphylococcus arlettae*, 1 coagulase-negative *Staphylococcus spp.*, and 1 *Staphylococcus waneri*).

13.Please corelate conclusion with your findings.

Response: Thank you very much, we have correlated our conclusion with our findings.

Lines 522-541 now read: We presented aerobic blood culture findings from a multi-centre study of patients with acute febrile illness admitted to eight major hospitals across Indonesia. Our universal use of aerobic blood cultures is unique in Indonesia, the results of which help clarify the epidemiology and burden of BSI, rates of contamination among CAI, and common AMR patterns in Indonesia. Bacteremia was observed in 8.9% participants, with the most frequent pathogens being *Salmonella spp.*, *E. coli*, and *S. aureus*. Two *Salmonella spp.* cases had evidence of AMR, and several *E. coli* cases were multidrug resistant (42.9%) or monoresistant (14.3%). Culture contamination was observed in 3.6% cases. Our data suggest that blood cultures should be included as a routine diagnostic test, and pre-screening patients for the most common viral infections, such as dengue, influenza and chikungunya viruses, would conserve scarce resources without negatively impacting patient benefit. The routine practice of AMR susceptibility testing on positive blood cultures in Indonesia is encouraging and should be continued to inform clinical decisions on patient treatment in real-time. The country could benefit from clear guidance at the national level, particularly regarding the timing of blood collection prior to antibiotic administration, the prioritization of patients with comorbidities, blood collection practices to reduce environmental contamination, and the supplementation of blood cultures with molecular assays to combat false-negative results. Additionally, Indonesia could greatly benefit from a nationwide program for the systematic collection and dissemination of blood culture and AMR results.

____

6. PLOS authors have the option to publish the peer review history of their article (what does this mean?). If published, this will include your full peer review and any attached files.

If you choose “no”, your identity will remain anonymous but your review may still be made public.

Do you want your identity to be public for this peer review? For information about this choice, including consent withdrawal, please see our Privacy Policy.
Reviewer #1: Yes: Dr Shishir Gokhale
Reviewer #2: No
