# Supplementary material for: The characteristics of bacteremia among patients with acute febrile illness requiring hospitalization in Indonesia
Source: PLoS One. 2022 Sep 8;17(9):e0273414. doi: 10.1371/journal.pone.0273414 (PMC9455855; doi:10.1371/journal.pone.0273414)
Supplement: Attachment — Submitted filename: Response to Reviewer - Blood Culture.docx [file pone.0273414.s002.docx]

Comments to the Author

1. Is the manuscript technically sound, and do the data support the conclusions?

The manuscript must describe a technically sound piece of scientific research with data that supports the conclusions. Experiments must have been conducted rigorously, with appropriate controls, replication, and sample sizes. The conclusions must be drawn appropriately based on the data presented.

Reviewer #1: Yes

Reviewer #2: Yes

______________

2. Has the statistical analysis been performed appropriately and rigorously?

Reviewer #1: Yes

Reviewer #2: Yes

______________

3. Have the authors made all data underlying the findings in their manuscript fully available?

The PLOS Data policy requires authors to make all data underlying the findings described in their manuscript fully available without restriction, with rare exception (please refer to the Data Availability Statement in the manuscript PDF file). The data should be provided as part of the manuscript or its supporting information, or deposited to a public repository. For example, in addition to summary statistics, the data points behind means, medians and variance measures should be available. If there are restrictions on publicly sharing data—e.g. participant privacy or use of data from a third party—those must be specified.

Reviewer #1: Yes

Reviewer #2: Yes

______________

4. Is the manuscript presented in an intelligible fashion and written in standard English?

PLOS ONE does not copyedit accepted manuscripts, so the language in submitted articles must be clear, correct, and unambiguous. Any typographical or grammatical errors should be corrected at revision, so please note any specific errors here.

Reviewer #1: Yes

Reviewer #2: No

______________

5. Review Comments to the Author

Please use the space provided to explain your answers to the questions above. You may also include additional comments for the author, including concerns about dual publication, research ethics, or publication ethics. (Please upload your review as an attachment if it exceeds 20,000 characters)

Reviewer #1: This compilation of data from different centers over many years is commendable. This highlights the issues faced in diagnostic microbiology in developing countries. It is an interesting paper with important observations and discussions. Some spellings need review and correction. Recommend to submit after corrections.

**Response**: Thank you very much for your comments, we really appreciate it.

We have corrected the spelling errors.

Reviewer #2: The Characteristics of Bacteremia among Patients with Acute Febrile Illness Requiring Hospitalization in Indonesia.

Evaluation. This report addresses an important subject in Bacteriemia and Acute Febrile illness; i.e., the worrying trend of antimicrobial resistance in bacterial pathogens (Salmonella and Non Salmonella spp) . It reports the frequency and distribution of bacterial pathogens in blood culture and its susceptibility pattern isolated from various specimens from a seven medical center in Indonesia, from which similar reports are scarce. Though it is better attempt by Soedarmono et al., to know information on bacteremia and other causative agent of Acute Febrile illeness in Indonesia.

**Response**: Thank you very much for your comments, we really appreciate it.

Comments

1.Give rationale of the study? Why is NS1 antigen screening only performed? What about other viral agents related AFI?

**Response**: We have added more information regarding this issue in the Methods.

Lines 135-146 now read: During the baseline visit, blood was collected for cultures, clinically relevant rapid diagnostic tests when available, and dengue virus rapid diagnostic tests. Dengue virus infection remains a significant burden across Indonesia [28,29], with disease incidence increasing in recent years [30]. Though other viral agents are present in Indonesia, none are as prevalent as dengue virus [24,31], and most are challenging to diagnose due to limitations with available rapid diagnostic tests [32,33]. Given the widespread prevalence of dengue virus infection, and the very high specificity (almost 100%) and good sensitivity (70-87%) of NS1 antigen rapid diagnostic tests [34], we employed universal dengue virus screening to rapidly resolve the unknown etiologies of study participants. Participants with negative NS1 antigen tests were further considered for BSIs through blood culture tests and other etiologies, as determined through advanced testing at the INA-RESPOND reference laboratory.

2.Why you performed Blood culture 0f 1459 Cases? You have mentioned 1464 were enrolled? What about 5??

**Response**: We only performed blood culture for 1459 patients, as the remaining 5 subjects did not have enough blood for blood culture test.

Lines 207-210 now read: The remaining 5 participants had insufficient blood specimens for following reasons: 1 adult was in a severe condition (decreased of consciousness), 2 participants (1 child and 1 adult) self-discharged against medical advice, and the guardians of 2 children refused to allow more blood to be drawn.

3.At the end of introduction, please give some update of Acute Febrile illness and their epidemiology in Indonesia.

**Response**: Thank you very much for the suggestion. We have added some update of acute febrile illness and their epidemiology in Indonesia.

Lines 97-111 now read: The epidemiology of pathogens associated with fever in Indonesia is not well understood, as public health surveillance data is limited and only a few local studies have been conducted [19,21–26]. Among published studies, dengue virus, chikungunya virus, influenza virus, *Salmonella* Typhi, *Rickettsia spp.*, and *Leptospira spp.* are consistently the most common causes of acute febrile illness hospitalizations. A study in Papua from November 1997 to February 2000 enrolled 226 hospitalized patients that were negative for malaria, the majority of whom were determined to have typhoid fever (18%), leptospirosis (12%), rickettsioses (8%), and dengue fever (7%) [23]. An observational fever study in Bandung identified dengue virus in 12.4% of fever episodes, followed by *S.* Typhi (7.4%), and chikungunya virus (7.1%) [24,26,27]. A 2005-2006 study in Semarang found rickettsioses and leptospirosis in 7% and 10%, respectively, of 137 acute undifferentiated fever cases [21]. The parent study of the research presented here found the most prevalent pathogens among participants at eight hospitals in 7 major cities in Indonesia to be dengue virus (27-52%), *Rickettsia spp.* (2-12%), *S.* Typhi (0.9-13%), influenza virus (2-6%), *Leptospira spp.* (0-5%), and chikungunya virus (0-4%) [19].

4.Which are the hospitals included in the study, please mentions the name of hospitals.

**Response**: We have included the name of hospitals in the Methods.

Lines 121-127 now read: A prospective observational study enrolling febrile patients who required hospitalization was conducted by the Indonesia Research Partnership on Infectious Disease (INA-RESPOND) from July 2013 to June 2016 at eight major hospitals in seven provincial capitals in Indonesia: Dr. Cipto Mangunkusumo Hospital in Jakarta, Sulianti Saroso Infectious Disease Hospital in Jakarta, Dr. Wahidin Sudirohusodo Hospital in Makassar, Dr. Sardjito Hospital in Yogyakarta, Dr. Hasan Sadikin Hospital in Bandung, Sanglah General Hospital in Denpasar, Dr. Soetomo Hospital in Surabaya, and Dr. Kariadi Hospital, in Semarang.

5.How do you calculate sample size? Is it sufficient to draw conclusion regarding bacteremia (causative bacterial pathogens) in Indonesia?

**Response**: As this study was an observational study to find etiologies of acute febrile illness during a certain period of time (2013-2016), we did not specifically calculate the sample size for drawing the conclusion regarding bacteremia in Indonesia. Since we performed the analysis of blood culture results from almost all participants (>99% participants, approximately 100 adults and 100 children from each hospital), though cannot be generalizable to the Indonesian population at-large, we expected that the data will provide better understanding of the bacteremia in hospitalized population with fever and hopefully will lead to a reduction in mortality from BSIs.

6.What is your inclusion and exclusion criteria? Please mention Clearly.

**Response**: We have added the inclusion and exclusion criteria.

Lines 128-131 now read: Briefly, inclusion criteria consisted of axillary body temperature ≥38˚C, ≥1 year of age, and hospitalization within the past 24 hours. Patients were excluded from the study if they had subjective fever for ≥14 days or were hospitalized in the last 3 months.

7.Please give the ethical approval committee name and approval number and date.

**Response**: The name of the ethical approval committee and approval number had already provided under the “Ethical Clearance” (lines 197-203); and we have added the date.

Ethical approvals for the AFIRE study were granted by the Institutional Review Boards of the National Institute of Health Research and Development (NIHRD), Indonesia Ministry of Health (KE.01.05/EC/407/2012) dated 23 May 2012, the Faculty of Medicine at the University of Indonesia and RSUPN Dr. Cipto Mangunkusumo Hospital (451/PT02.FK/ETIK/2012) dated 23 July 2012, and RSUD Dr. Soetomo Hospital (192/Panke.KKE/VIII/2012) dated 13 August 2012.

8.How do assure the Quality controls and quality check of your results, either BD

135 Phoenix (Becton Dickinson) or VITEK 2 (bioMérieux, Inc., Durham, North Carolina), System?

**Response**: Blood culture tests were performed at the hospital's accredited clinical laboratory, which provides patient diagnostic services. All instruments and standards were calibrated appropriately according to manufacturer guidelines. Every site’s laboratory performed quality control (QC) to ensure proper performance and sent the QC report to protocol team to be reviewed. All tests were run alongside appropriate positive and negative control to ensure the integrity and accuracy of the results. For example, QC for VITEK 2 system; each new lot number of ID cards is tested with stock culture organisms. Susceptibility cards are tested weekly against stock culture organisms.

The QC organisms uses as follows:

Weekly:

AST-GP 67 cards

*Enterococcus faecalis* ATCC 29212

AST-GN 66 cards

*E. coli* ATCC 25922 non fermenter

PSA ATCC 27853 fermenter

*E. coli* ATCC 35218 non fermenter

ID-NH cards

*Elkenella corrodens* ATCC BAA-1152

New Lots:

ID-GP cards

*Enterococcus casseliflavis* ATCC 700327

ID-GN cards

*Enterobacter hormechei* (*E.cloacae*) ATCC 700323

Lines 163-171 now read: Blood cultures were performed and analyzed at the hospitals’ nationally accredited clinical laboratories by trained, certified staff. All instruments and standards were calibrated appropriately according to manufacturer guidelines, and all tests were run alongside appropriate positive and negative control to ensure the integrity and accuracy of the results. Organism identification was considered acceptable when the confidence level in the automated growth identification system was ≥95% probability [34]. Quality control tests were performed weekly at all site laboratories, and each new lot of ID cards was tested using validated stocks of culture organisms.

9.What is the volume of blood sample collected and used in culture from children and adults?

**Response:** This is already stated in the text. Blood volumes of approximately 5-8 mL for adults and 1-3 mL for children were collected from each arm, whenever possible, directly into separate aerobic blood culture bottles (lines 150-152).

10.It is better to give numerator value after percentage values.

**Response**: We have changed the presentation throughout the manuscript.

11.Please give the full name of bacteria initially such as Staphylococcus aureus and then short form S. aureus and other bacteria throughout the manuscript.

**Response**: We have followed your suggestion.

12.Please mention the more information on infections with dengue virus and bacteremia in Indonesia.

**Response**: We found no dengue virus and bacteremia co-infection in our study, as mentioned in the Discussion. We have added more informations about dengue virus and bacteremia.

Lines 355-368 now read: Data on co-infections with dengue virus and bacteremia is limited. A literature review of published case reports and studies from January 1943 to March 2016 found 3 studies in Singapore and Taiwan reporting concurrent bacteremia in 0.18-7% of dengue fever cases [40–42]. A concurrent dengue virus and *S.* Typhi case was also reported from Bandung, Indonesia [43]. In all of these studies, blood was collected for bacterial culture because patients did not improve clinically a few days to a week after dengue fever was diagnosed. Furthermore, in the majority of cases, dengue virus infection was confirmed by serology only (IgM detected or four-fold IgG increase). These reports support our finding that simultaneous infection with bacteria and dengue virus is rare. In our study, bacterial growth observed in 14 participants with positive dengue NS1 antigen tests were considered false positive blood cultures (5 *Staphylococcus hominis*, 4 *Staphylococcus epidermidis*, 1 *Kocuria rosea*, 1 *Micrococcus aureus*, 1 *Staphylococcus arlettae*, 1 coagulase-negative *Staphylococcus spp.*, and 1 *Staphylococcus waneri*).

13.Please corelate conclusion with your findings.

**Response:** Thank you very much, we have correlated our conclusion with our findings.

Lines 522-541 now read: We presented aerobic blood culture findings from a multi-centre study of patients with acute febrile illness admitted to eight major hospitals across Indonesia. Our universal use of aerobic blood cultures is unique in Indonesia, the results of which help clarify the epidemiology and burden of BSI, rates of contamination among CAI, and common AMR patterns in Indonesia. Bacteremia was observed in 8.9% participants, with the most frequent pathogens being *Salmonella spp.*, *E. coli*, and *S. aureus*. Two *Salmonella spp.* cases had evidence of AMR, and several *E. coli* cases were multidrug resistant (42.9%) or monoresistant (14.3%). Culture contamination was observed in 3.6% cases. Our data suggest that blood cultures should be included as a routine diagnostic test, and pre-screening patients for the most common viral infections, such as dengue, influenza and chikungunya viruses, would conserve scarce resources without negatively impacting patient benefit. The routine practice of AMR susceptibility testing on positive blood cultures in Indonesia is encouraging and should be continued to inform clinical decisions on patient treatment in real-time. The country could benefit from clear guidance at the national level, particularly regarding the timing of blood collection prior to antibiotic administration, the prioritization of patients with comorbidities, blood collection practices to reduce environmental contamination, and the supplementation of blood cultures with molecular assays to combat false-negative results. Additionally, Indonesia could greatly benefit from a nationwide program for the systematic collection and dissemination of blood culture and AMR results.

______________

6. PLOS authors have the option to publish the peer review history of their article (what does this mean?). If published, this will include your full peer review and any attached files.

If you choose “no”, your identity will remain anonymous but your review may still be made public.

Do you want your identity to be public for this peer review? For information about this choice, including consent withdrawal, please see our Privacy Policy.

Reviewer #1: Yes: Dr Shishir Gokhale

Reviewer #2: No
