# Supplementary material for: The characteristics of bacteremia among patients with acute febrile illness requiring hospitalization in Indonesia
Source: PLoS One. 2022 Sep 8;17(9):e0273414. doi: 10.1371/journal.pone.0273414 (PMC9455855; doi:10.1371/journal.pone.0273414)
Supplement: Attachment — Submitted filename: Response to Editor.docx [file pone.0273414.s004.docx]

Journal Requirements:

Please review your reference list to ensure that it is complete and correct. If you have cited papers that have been retracted, please include the rationale for doing so in the manuscript text, or remove these references and replace them with relevant current references. Any changes to the reference list should be mentioned in the rebuttal letter that accompanies your revised manuscript. If you need to cite a retracted article, indicate the article’s retracted status in the References list and also include a citation and full reference for the retraction notice.

**Response**: We have reviewed our reference list to ensure that it is complete and correct. As per today (27 July 2022), no cited papers have been retracted. Therefore, no change of the reference list has been made.

Additional Editor Comments:

manuscript requires few corrections in the section abstract method result discussion and conclusion, minor revision required . Please go through the comments made in manuscript. Serovar names of salmonella isolates obtained during present study be mentioned whereever required in the manuscript

**Response**: Thank you very much. We have gone through the comments made in manuscript and revised the manuscript accordingly.

When required/appropriate, we have reported names of salmonella isolates obtained during the present study throughout the manuscript (41 cases of S. Typhi and 10 cases of S. Paratyphi A). However, we have not mentioned the serovar names when we refer the subjects with *Salmonella spp.* and non-*Salmonella spp.* as groups.

Abstract (lines 55-61 now read): Bacteremia was observed in 8.9% (92) participants, with the most frequent pathogens being *Salmonella enterica serovar* Typhi (41) and Paratyphi A (10), *Escherichia coli* (14), and *Staphylococcus aureus* (10). Two *S.* Paratyphi A cases had evidence of AMR, and several *E. coli* cases were multidrug resistant (42.9%, 6/14) or monoresistant (14.3%, 2/14). Culture contamination was observed in 3.6% (37) cases. Molecular and serological assays identified etiological agent in participants having negative cultures, with 23.1% to 90% of cases being missed by blood cultures.

Other changes have been made with “track changes” in the manuscript.
